# Structures of ALG3/9/12 reveal the assembly logic of the *N*-glycan oligomannose core

**DOI:** 10.1038/s41589-026-02164-7

**Published:** 2026-03-10

**Authors:** J. Andrew N. Alexander, Shu-Yu Chen, Somnath Mukherjee, Mario de Capitani, Rossitza N. Irobalieva, Lorenzo Rossi, Parth Agrawal, Julia Kowal, Matheus A. Meirelles, Markus Aebi, Jean-Louis Reymond, Anthony A. Kossiakoff, Sereina Riniker, Kaspar P. Locher

**Affiliations:** 1 Institute of Molecular Biology and Biophysics, Eidgenössische Technische Hochschule (ETH) Zürich, Zurich, Switzerland; 2 Department of Chemistry and Applied Biosciences, Eidgenössische Technische Hochschule (ETH) Zürich, Zurich, Switzerland; 3 Department of Biochemistry and Molecular Biology, University of Chicago, Chicago, IL USA; 4 Department of Chemistry and Biochemistry, University of Bern, Bern, Switzerland; 5 Institute of Microbiology, Eidgenössische Technische Hochschule (ETH) Zürich, Zurich, Switzerland

**Keywords:** Structural biology, Glycobiology

## Abstract

Asparagine-linked glycans are essential for the maturation and function of most eukaryotic secretory proteins. The biosynthesis and transfer of dolichylpyrophosphate-anchored GlcNAc_2_Man_9_Glc_3_ glycan is a highly conserved process occurring in the endoplasmic reticulum (ER) membrane and involving over a dozen membrane proteins whose dysfunction is linked to congenital disorders of glycosylation (CDGs). Three membrane-integral mannosyltransferases, ALG3, ALG9 and ALG12, mediate four consecutive mannosylation reactions that convert GlcNAc_2_Man_5_ to GlcNAc_2_Man_9_. Here, using chemoenzymatically synthesized lipid-linked glycan donor and acceptor analogs, we recapitulated this biosynthetic pathway in vitro. High-resolution cryo-electron microscopy structures of pseudo-Michaelis complexes of each step revealed how the branched glycan is accurately synthesized and unwanted side products are averted. Molecular dynamics simulations and mutagenesis studies uncovered a subtle but precise mechanism selecting the dolichylphosphomannose donor substrate over dolichylphosphoglucose, which is also present in the ER membrane. Our results also provide mechanistic explanations for enzyme dysfunction in CDGs and offer opportunities for *N*-glycan engineering.

**Figure:**
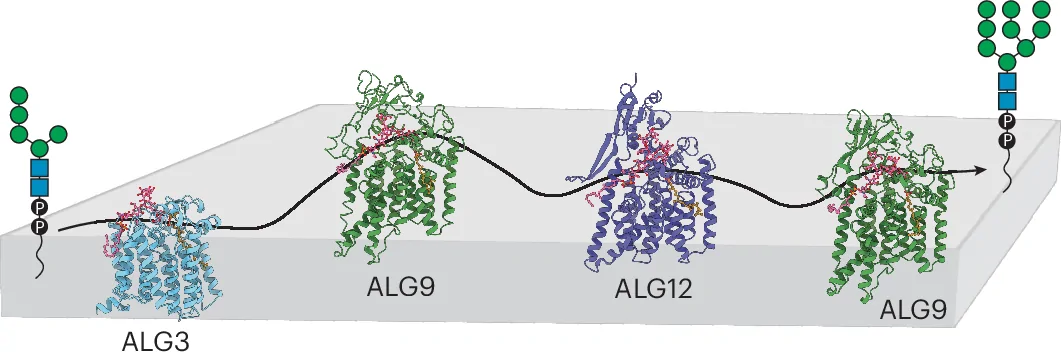

## Abstract (web summary)

Three endoplasmic reticulum luminal mannosyltransferases ALG3, ALG9 and ALG12 catalyze the synthesis of the branched mannose core in *N*-glycans in four distinct steps. Now, cryo-electron microscopy structures and characterization of ALG3, ALG9 and ALG12 reveal the molecular logic of oligomannose core assembly.

## Main

The addition of glycans to protein asparagine residues, termed *N*-linked glycosylation, is found throughout the three domains of life and is one of the most common post-translational modifications^1,2^. Having primarily structural functions at the cell surface in archaea and bacteria, the functional and structural diversity of *N*-linked glycans exploded during eukaryotic evolution. In the endoplasmic reticulum (ER), *N*-linked glycans have pivotal roles in protein folding and stabilization, as well as in directing protein localization, degradation and sorting^3–5^. These processes are highly conserved in eukaryotes and involve unique and precisely defined *N*-glycan structures that serve as covalently attached signals, providing the basis of the generation of highly diverse secretory proteins^6^. Starting from the uniform oligomannose structures exiting the ER, hydrolases and glycosyltransferases in the Golgi compartments generate a large array of distinct glycan structures that are species, protein and glycosylation site specific. Thus, a kinetically controlled pathway that is based on the differential localization of differentially expressed processing enzymes yields a large diversity of *N*-glycan structures that mediate cell–cell and cell–molecule interactions, many of which are essential for immune function and host–pathogen interactions or have roles in cancer metastasis, nervous system development and function and Alzheimer disease^7–12^. In humans, the severe phenotypes associated with the more than 100 recorded congenital errors in this pathway demonstrate the biomedical importance of these glycan-mediated quality control steps^13,14^.

**Fig. 1:**
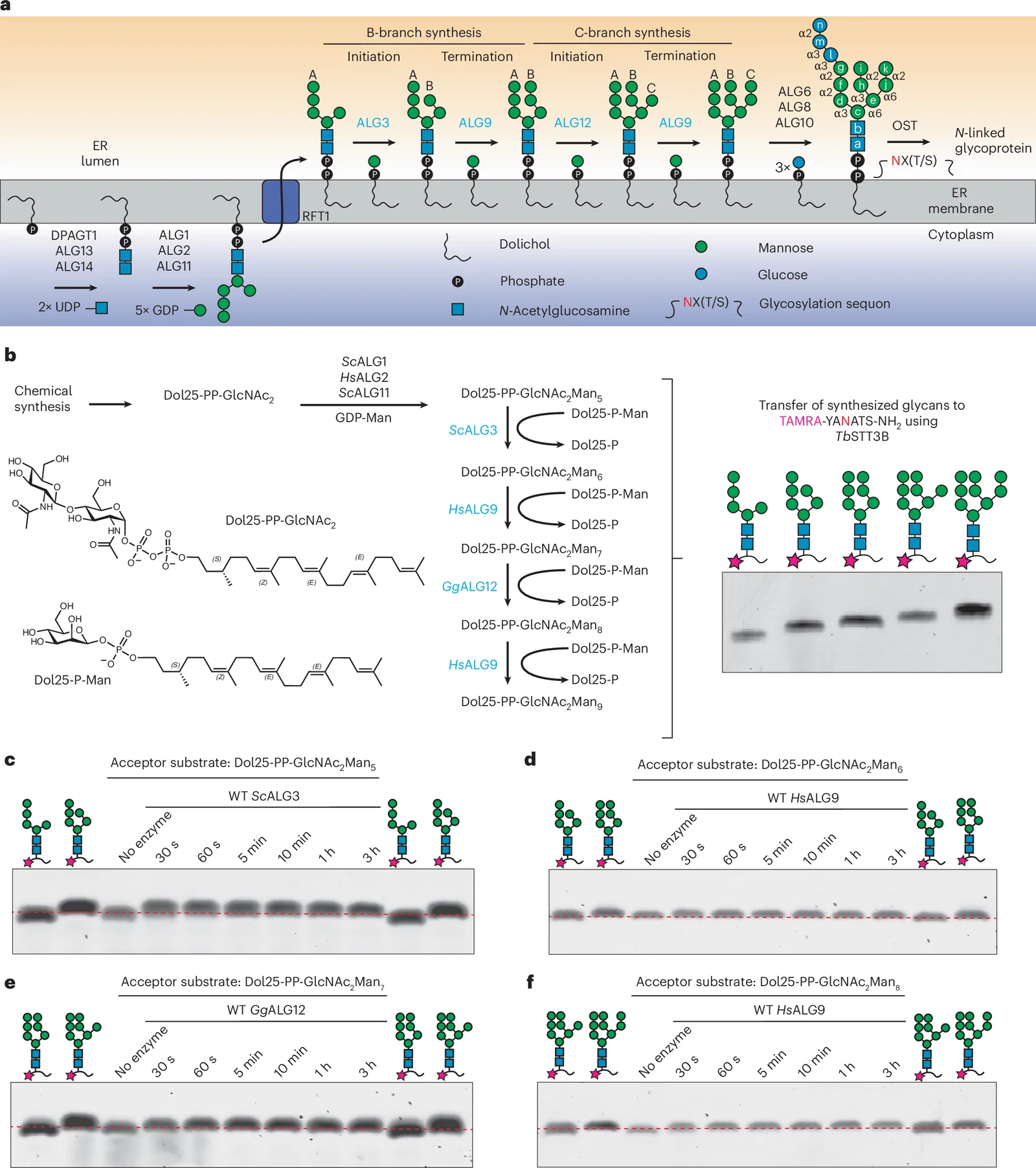
Functional reconstitution of oligomannose synthesis. **a**, Schematic of *N-*glycan synthesis in the human ER. **b**, Strategy for the chemoenzymatic synthesis of the substrates for the luminal ER mannosyltransferases. Structures of the chemically synthesized starting compounds, Dol25-PP-GlcNAc_2_ and Dol25-P-Man, are shown. The obtained glycans were transferred to a fluorescent peptide using *Tb*STT3B and separated on a tricine gel. Structures above the gel lanes are indicated according to the Symbol Nomenclature for Glycans. **c**–**f**, Time courses of enzymatic activity using WT *Sc*ALG3 and Dol25-PP-GlcNAc_2_Man_5_ (**c**), WT *Hs*ALG9 and Dol25-PP-GlcNAc_2_Man_6_ (**d**), WT *Gg*ALG12 and Dol25-PP-GlcNAc_2_Man_7_ (**e**) and WT *Hs*ALG9 and Dol25-PP-GlcNAc_2_Man_8_ (**f**). Incubation times were as indicated. Lanes with glycopeptide cartoons over them serve as glycopeptide standards for the indicated glycan. Activity assays in **b** –**f** were performed at least twice for each enzyme–substrate combination with similar results. Source data

Before acceptor protein glycosylation, *N-*glycans are synthesized as lipid-linked oligosaccharides (LLOs) covalently bound to dolichylpyrophosphate (Dol-PP) and, thus, anchored in the ER membrane^1^. The sequential addition of sugars is catalyzed by the asparagine-linked glycosylation (ALG) enzymes (Fig. 1a). Synthesis begins on the cytosolic face of the ER membrane, where DPAGT1, ALG13 and ALG14 convert dolichylphosphate (Dol-P) to Dol-PP-GlcNAc_2_ (refs. ^15,16^). Subsequently, ALG1, ALG2 and ALG11 sequentially transfer mannoses **c** to **g** (labeling as previously published^6^; legend in Fig. 1a) from GDP-mannose to the growing LLO. The resulting Dol-PP-GlcNAc_2_Man_5_ is then flipped to the ER lumen by RFT1 (refs. ^17,18^). Extension of the LLO in the ER lumen involves the transfer of four mannoses and three glucoses from dolichylphosphomannose (Dol-P-Man) and dolichylphosphoglucose (Dol-P-Glc) donors. The mannosyltransferases ALG3, ALG9 and ALG12 transfer four mannoses, initiating and completing the B and C branches of the growing the *N-*glycan (Fig. 1a)^19^. ALG3 initiates the B branch by adding the **h** mannose in an α1,3 linkage^20,21^. The B branch is then completed with an α1,2-linked addition of the **i** mannose by the activity of ALG9 (refs. ^22,23^). Only when the B branch is complete does ALG12 initiate the C branch with the addition of the α1,6-linked **j** mannose^24^. The C branch is completed by ALG9, which adds the α1,2-linked **k** mannose^22^. The GlcNAc_2_Man_9_ glycan is subsequently processed by the enzymes ALG6, ALG8 and ALG10, which sequentially transfer three glucoses to the A branch^25–27^. The completed GlcNAc_2_Man_9_Glc_3_ oligosaccharide is transferred to target proteins containing an *N*-glycosylation sequon, Asn-Xaa-Ser/Thr, by the oligosaccharyltransferase (OST) complex^1,28–31^.

Here, we recapitulate oligomannose *N-*glycan synthesis in vitro and report structures of ALG3, ALG9 and ALG12 bound to chemoenzymatically generated substrate analogs. In combination with mutagenesis studies and molecular dynamics (MD) simulations, these structures reveal the mechanism and biosynthetic logic of ER luminal *N*-glycan B-branch and C-branch synthesis. Our results also foster a molecular understanding of associated congenital disorders of glycosylation (CDGs) and advance efforts to engineer therapeutics and vaccines bearing *N-*glycans.

## Results and discussion

### Biochemical characterization of ALG3, ALG9 and ALG12

**Extended Data Fig. 1:**
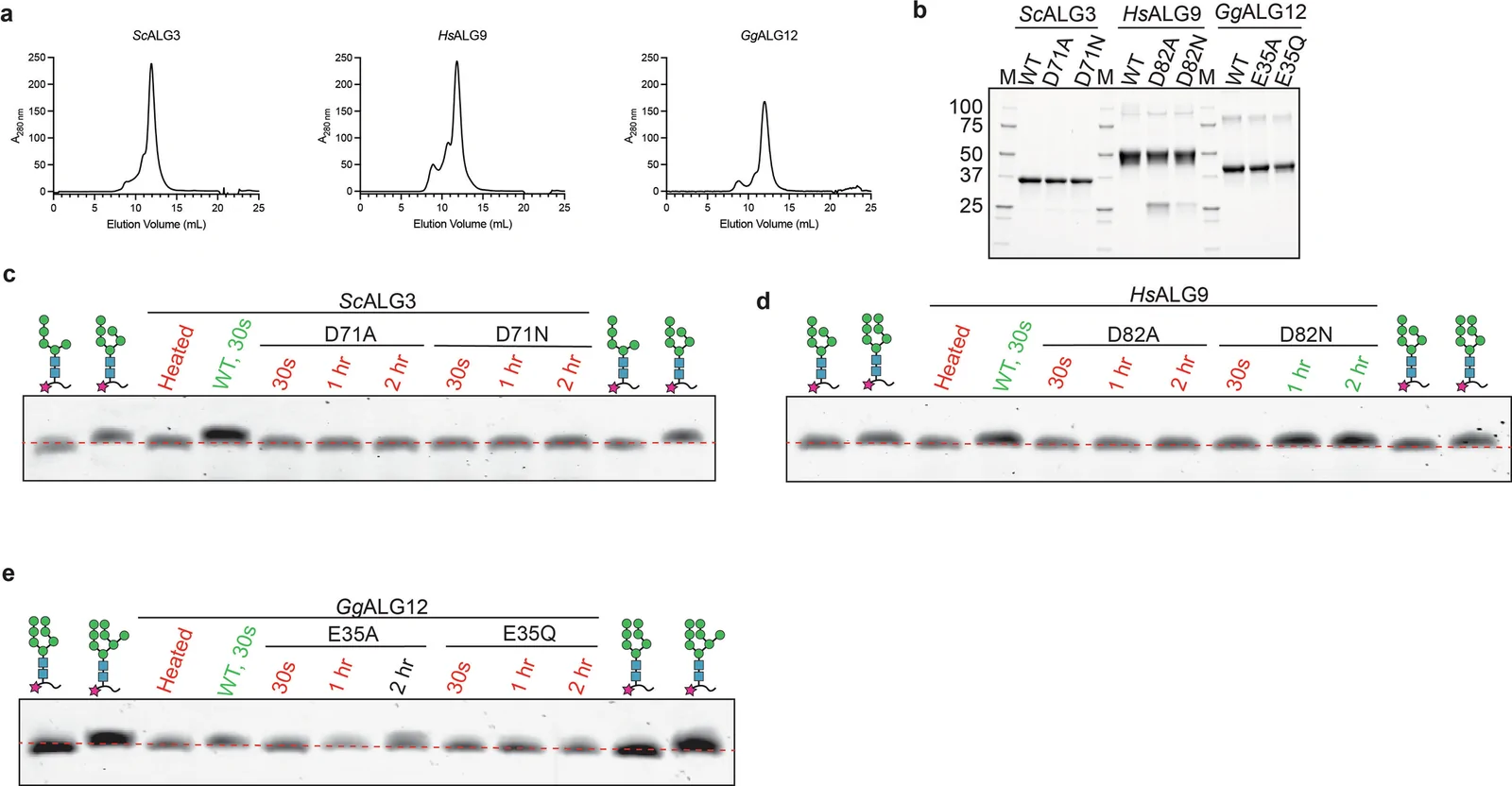
Biochemical and functional characterisation of *Sc*ALG3, *Hs*ALG9 and *Gg*ALG12. **a**, Size exclusion chromatography of *Sc*ALG3, *Hs*ALG9 and *Gg*ALG12 solubilized in LMNG-CHS detergent. **b**, SDS-PAGE analysis of ScALG3, HsALG9 and GgALG12. Representative gel shown from multiple (n > 10) independent purifications of ALG3, ALG9 and ALG12. Molecular weight markers are indicated in kDa. **c-e**, Assays with WT and catalytic mutants of **c**, *Sc*ALG3, **d**, *Hs*ALG9 and **e**, *Gg*ALG12 in LMNG-CHS. The reaction products were transferred to a fluorescent peptide (TAMRA-YA**N**ATS-NH_2_) using *Tb*STT3B and separated by tricine gel electrophoresis. The reaction times for each sample are indicated above the lanes and are coloured red for inactive, green for active and black for partially active. Lanes with glycopeptide cartoons over them serve as glycopeptide standards for the indicated glycan. Activity assays for each construct were performed at least twice with similar results. Source data

Gene homologs encoding ALG3, ALG9 and ALG12 from several species were expressed in HEK293 cells and screened using florescence size-exclusion chromatography (SEC) to assess protein expression and monodispersity^32^. On the basis of these results, we selected ALG3 from *Saccharomyces* *cerevisiae* (*Sc*ALG3), ALG9 from *Homo* *sapiens* (*Hs*ALG9) and ALG12 from *Gallus* *gallus* (*Gg*ALG12) for structural and functional studies (Extended Data Fig. 1a,b). Because the native dolichol contains 14–19 isoprenoid units, Dol-P-linked or Dol-PP-linked monosaccharides or glycans are poorly soluble and challenging to synthesize^19,33^. We, therefore, used synthetic or chemoenzymatically generated substrate analogs with a 25-carbon farnesylcitronellyl moiety, here referred to as Dol25, which increases their solubility^34,35^(Fig. 1b). The various acceptor substrates were chemoenzymatically generated using synthetic Dol25-PP-GlcNAc_2_, which was enzymatically extended to GlcNAc_2_Man_5_ using purified ALG1, ALG2 and ALG11 enzymes and GDP-Man as the mannose donor^34^. The chemoenzymatically generated LLOs could be transferred to a fluorescently labeled peptide using purified STT3B from *Trypanosoma* *brucei* (*Tb*STT3B), a single-subunit OST^36^. The resulting glycopeptides were analyzed using tricine–SDS–PAGE (Fig. 1b). In vitro activity assays were performed by allowing purified enzyme to transfer mannose from Dol25-P-Man to the provided acceptor LLO before enzyme denaturation. These assays demonstrated that detergent-solubilized *Sc*ALG3, *Hs*ALG9 and *Gg*ALG12 were catalytically active (Fig. 1c–f). Assays with Dol25-PP-GlcNAc_2_Man_6_ or Dol25-PP-GlcNAc_2_Man_8_ acceptor substrates confirmed the dual activity of ALG9 in vitro (Fig. 1d,f). Prolonged incubation times with each of these enzymes showed that they specifically add a single mannose, without further extension (Fig. 1c–f).

### High-affinity antigen-binding fragment (Fab) selection using a synthetic library

**Extended Data Fig. 2:**
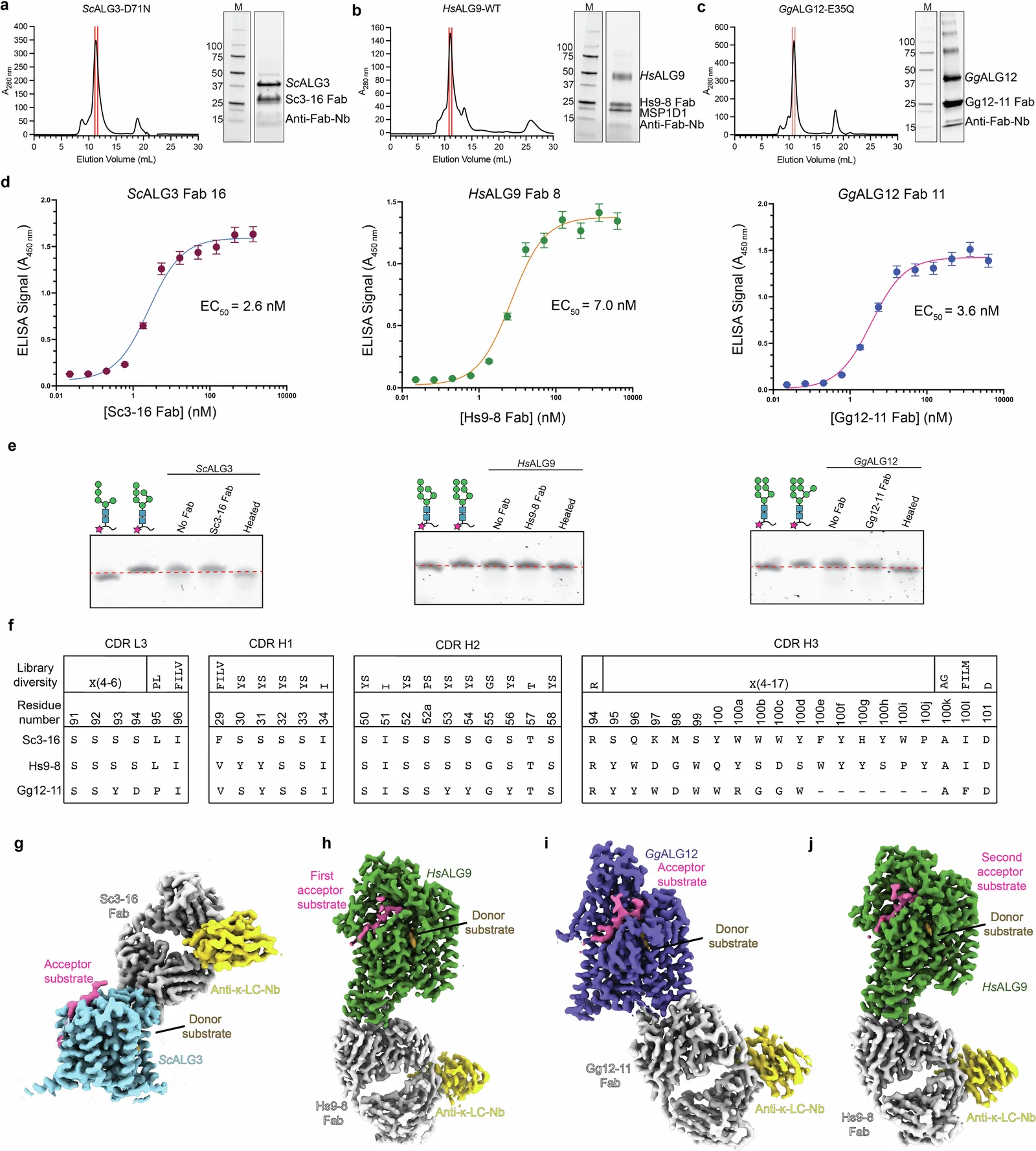
Biochemical characterisation of *Sc*ALG3, *Hs*ALG9 and *Gg*ALG12 Fabs. **a-c**, Size exclusion chromatography and SDS-PAGE analysis of *Sc*ALG3 D71N with Sc3-16 Fab **(a)**, *Hs*ALG9 WT with Hs9-8 Fab **(b)** and *Gg*ALG12 E35Q with Gg12-11 Fab **(c)**. In panels **a-c**, molecular weight markers are indicated in kDa. All samples in panels **a**-**c** included the anti-κ-light-chain nanobody. Purification of *Sc*ALG3, *Hs*ALG9 and *Gg*ALG12 were performed multiple times (n > 10) with similar results. Fractions taken for the SDS-PAGE gels are indicated with red lines. **d**, Multi-point ELISA binding curves for the indicated Fabs. Data (n = 3 biological replicates for Fab each concentration) are presented as mean values ± SD. **e**, Activity of *Sc*ALG3, *Hs*ALG9 and *Gg*ALG12 in the presence and absence of the Fabs in panels **a-c**. The reaction products were transferred to a fluorescent peptide (TAMRA-YA**N**ATS-NH_2_) using *Tb*STT3B and separated by tricine gel electrophoresis. Lanes with glycopeptide cartoons over them serve as glycopeptide standards for the indicated glycan. Activity assays with Fabs were performed at least three times with similar results. **f**, Sequences of the complementarity determining regions (CDRs) of each Fab used for structural studies. The Kabat numbering system is used for the Fab CDR residues^120^. **g-i**, Cryo-EM density maps of **g**, *Sc*ALG3 ternary, **h**, *Hs*ALG9 first ternary, **i**, *Gg*ALG12 ternary and **j**, *Hs*ALG9 second ternary complexes. The cryo-EM density maps are coloured according to the different components as labelled. Source data

To provide insight into the reaction mechanisms, high-resolution structures of each of the enzymes in complex with donor and acceptor substrates were required. Because they are small integral membrane proteins (*Sc*ALG3, 52.9 kDa; *Hs*ALG9, 69.9 kDa; *Gg*ALG12, 55.8 kDa), particle alignment during cryo-electron microscopy (cryo-EM) data processing is challenging^37^. We, therefore, generated Avi-tagged constructs of *Sc*ALG3, *Hs*ALG9 and *Gg*ALG12, which allowed in vitro biotinylation and subsequent screening of phage library E for specific, high-affinity synthetic Fabs^38–40^. This yielded the Fabs Sc3-16, Hs9-8 and Gg12-11, which formed high-affinity complexes with *Sc*ALG3, *Hs*ALG9 and *Gg*ALG12 respectively, as shown by SEC, SDS–PAGE and multipoint protein ELISA (Extended Data Fig. 2a–d). At a tenfold molar excess of each Fab, the mannosyltransferase activities of *Sc*ALG3, *Hs*ALG9 and *Gg*ALG12 were unaltered, demonstrating that the Fabs did not interfere with enzyme function (Extended Data Fig. 2e). The sequences of the complementarity-determining regions (CDRs) of Sc3-16, Hs9-8 and Gg12-11 are shown in Extended Data Fig. 2f.

### Structures of substrate-bound *Sc*ALG3, *Hs*ALG9 and *Gg*ALG12

**Extended Data Fig. 3:**
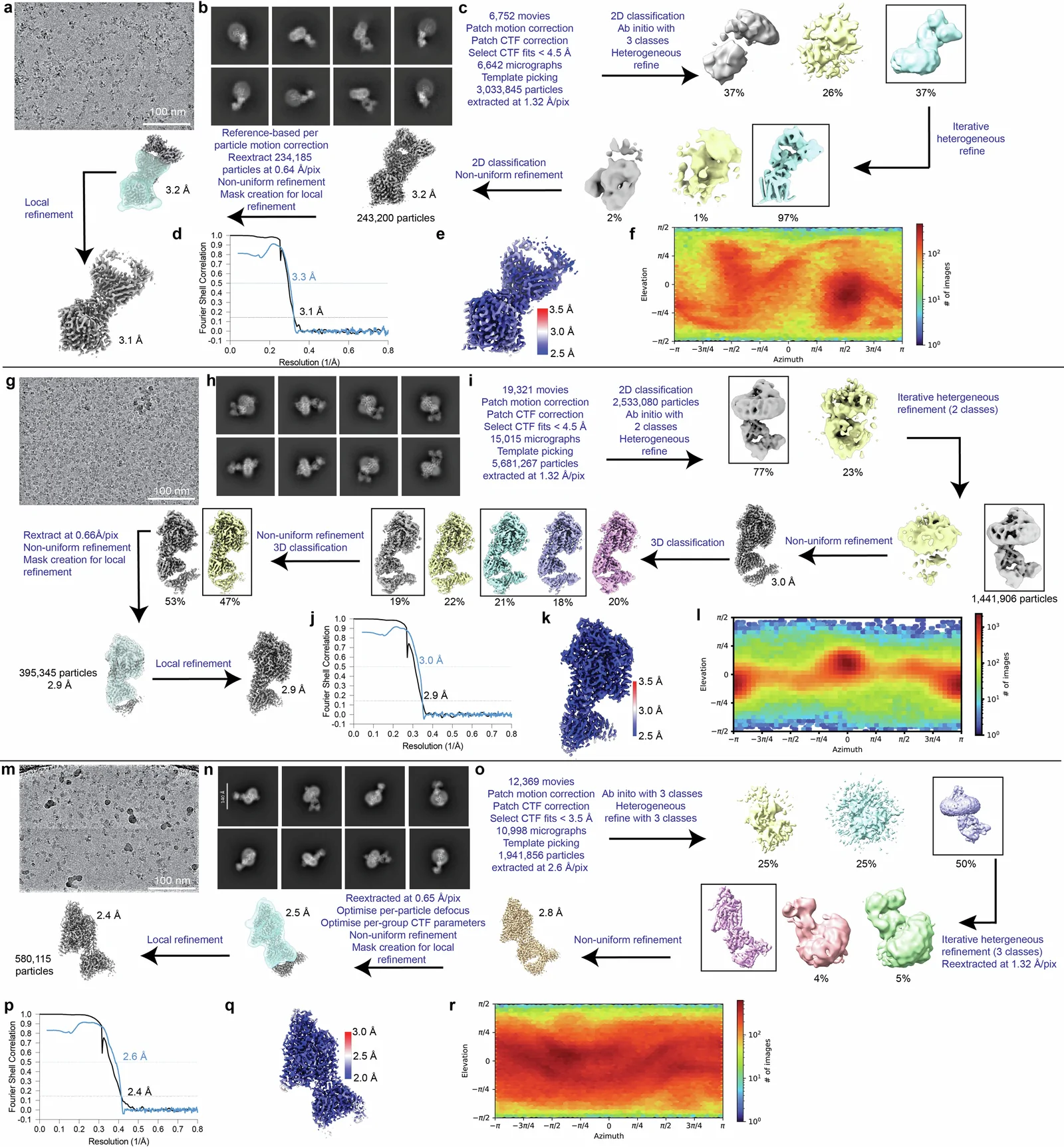
Single particle cryo-EM data collection and processing for *Sc*ALG3, *Hs*ALG9 and *Gg*ALG12 structures. Panels **a**-**f** pertain to the ALG3-ternary complex. **a**, Representative micrograph. **b**, Reference-free 2D class averages. **c**, Processing strategy in cryoSPARC^82^. **d**, Gold standard half-map and model-map FSC curves shown in black and blue lines, respectively. The dashed lines are shown at 0.143 and 0.5 FSC. **e**, Final map coloured by local resolution. **f**, Particle orientation distribution plot. Panels **g**-**l** pertain to the ALG9-first-ternary complex and are listed in order as panels **a**-**f**. Panels **m**-**r** pertain to the ALG12-ternary complex and are listed in order as panels **a**-**f**. The scale bar in panels **a**, **g** and **m** represents 100 nm.

To facilitate the trapping of pseudo-Michaelis complexes, we substituted the residues providing the putative catalytic base in *Sc*ALG3 (D71N), *Hs*ALG9 (D82A) and *Gg*ALG12 (E35Q) to either an alanine or the corresponding carboxamide side chain. The catalytic residues were predicted on the basis of sequence homology with other glycosyltransferase class C_A_ (GT-C_A_) enzymes^41^ and confirmed by activity assays, where the mutant enzymes showed strongly decreased mannosyltransferase activity (Extended Data Fig. 1c–e and Supplementary Figs. 1–3). Single-particle cryo-EM data of *Sc*ALG3-D71N, *Hs*ALG9-D82A and *Gg*ALG12-E35Q (referred to below as *Sc*ALG3, *Hs*ALG9 and *Gg*ALG12) in complex with Fab fragments and an anti-Fab nanobody targeting the κ light chain at the Fab hinge^42^ were collected and processed to resolutions ranging from 2.4 to 3.1 Å (Extended Data Table 1 and Extended Data Fig. 3). The Fabs targeting *Hs*ALG9 and *Gg*ALG12 bound the cytoplasmic face, while the Fab for *Sc*ALG3 bound the luminal face of the mannosyltransferases (Extended Data Fig. 2g–j). In each case, the high-resolution maps allowed de novo model building of the proteins and the bound substrate analogs.

**Fig. 2:**
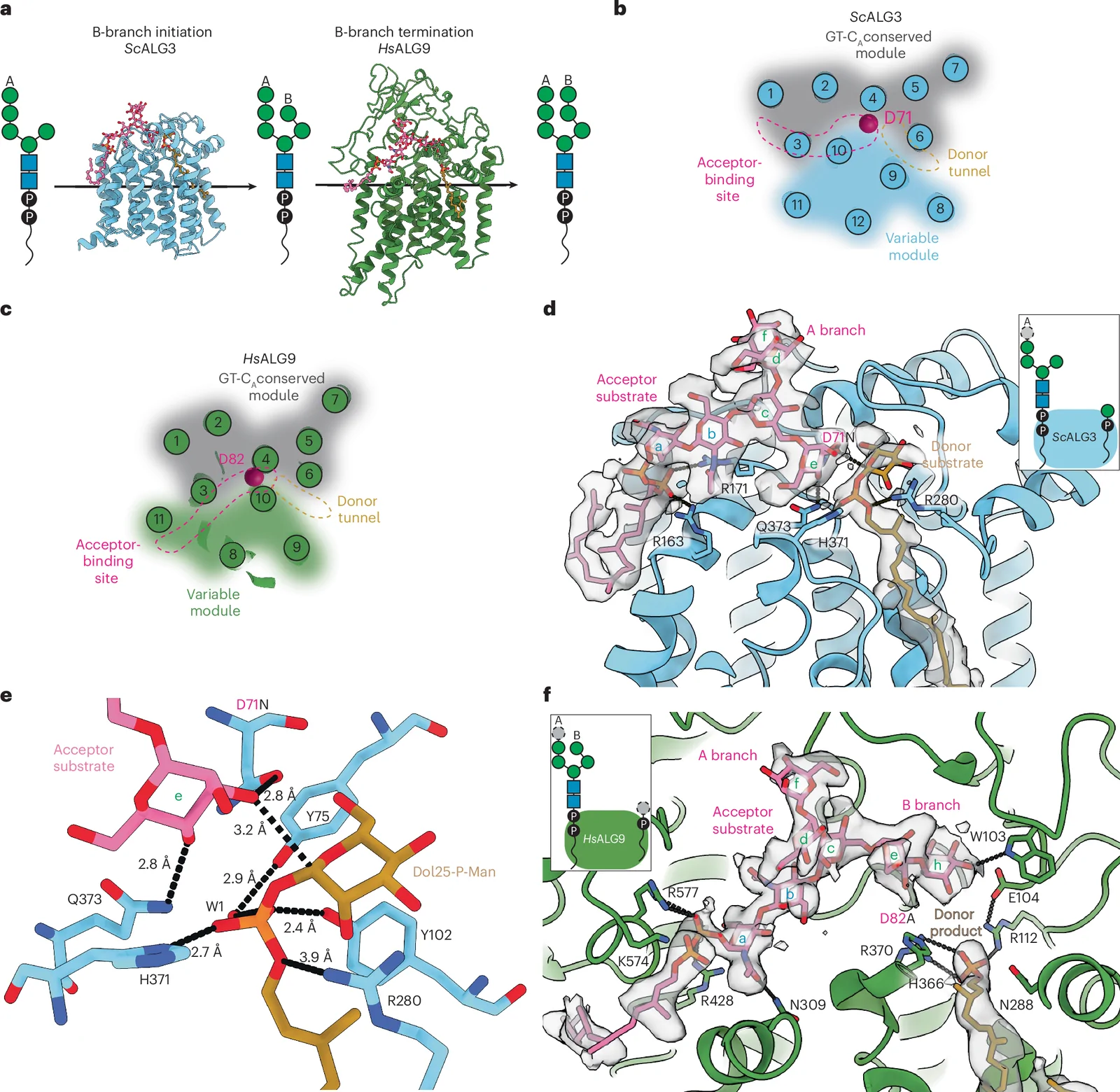
Oligomannose B-branch synthesis. **a**, Schematic showing the initiation and termination of the *N-*glycan B branch. *Sc*ALG3 and *Hs*ALG9 structures are displayed as ribbon diagrams, while the acceptor and donor substrates are shown as sticks in pink and gold, respectively. **b**,**c**, Cross-section schematic view of *Sc*ALG3 **(b)** and *Hs*ALG9 **(c)** viewed from the ER luminal side, indicating the arrangement of TMs and the substrate-binding pockets. The conserved and variable domains of the GT-C_A_ fold are indicated with gray and colored shading, respectively. The locations of catalytic base residues are indicated by magenta spheres. **d**, Structure of ternary complex of *Sc*ALG3-D71N with bound Dol25-PP-GlcNAc_2_Man_5_ (acceptor) and Dol25-P-Man (donor) substrates. **e**, Close-up view of the *Sc*ALG3 active site in stick representation. Carbons of protein residues are colored light blue, acceptor substrate in pink and donor substrate in gold. Selected distances are shown by dashed lines. **f**, Structure of the ternary complex of *Hs*ALG9-D82A with bound Dol25-PP-GlcNAc_2_Man_6_ acceptor substrate. Insets in **d**,**f** show cartoons of bound substrates, with monosaccharides resolved in the cryo-EM density colored according to the Symbol Nomenclature for Glycans, while unresolved monosaccharides are depicted in gray. In **d**,**f**, cryo-EM densities for bound substrates are shown as transparent gray surfaces. Acceptor and donor substrates are depicted in pink and gold stick representation. Key residues are highlighted as sticks and hydrogen-bonding interactions are shown as dashed lines.

**Fig. 3:**
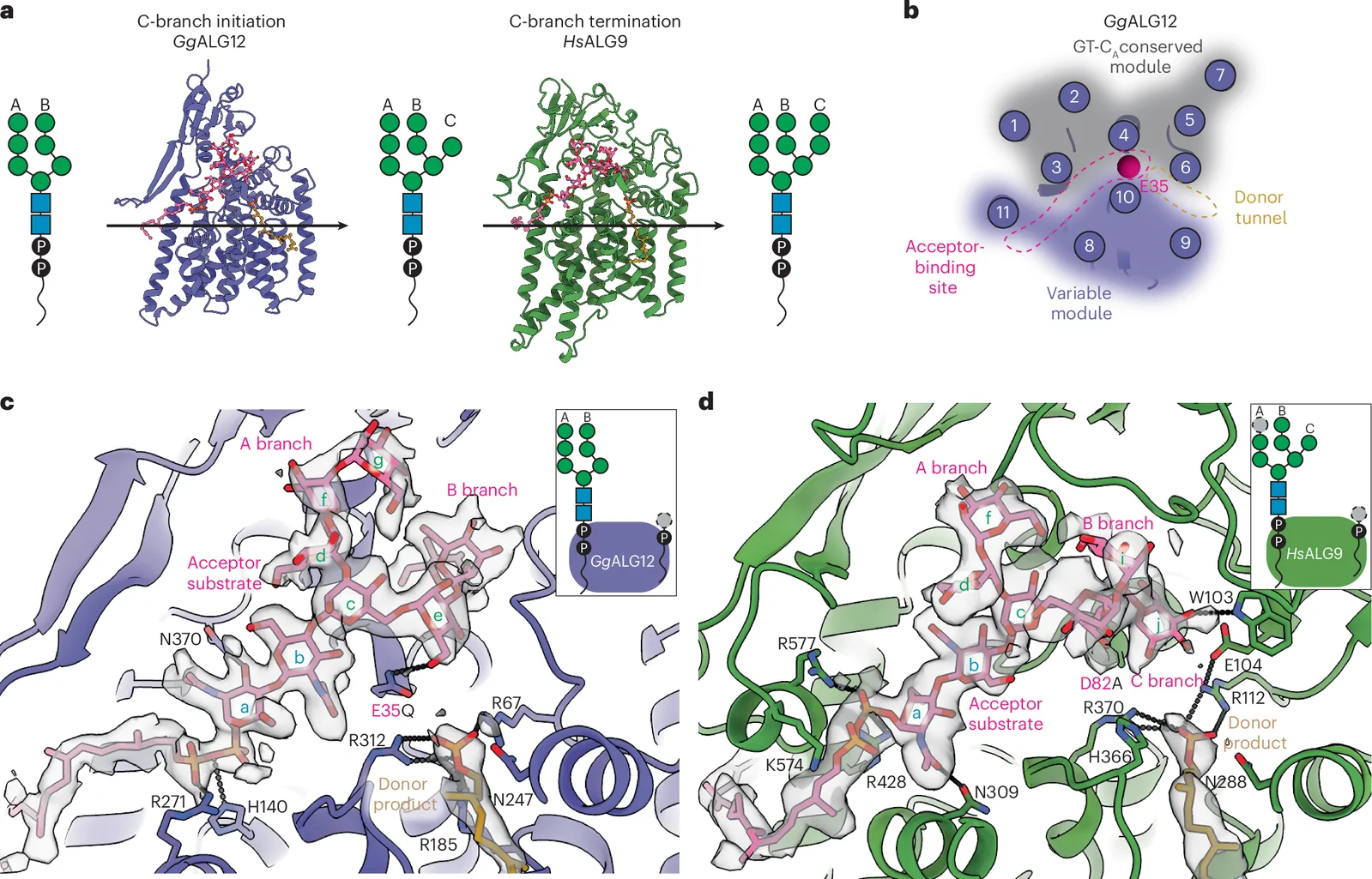
Oligomannose C-branch synthesis. **a**, Schematic showing synthesis of the *N-*glycan C branch. *Gg*ALG12 and *Hs*ALG9 structures are displayed as ribbon diagrams, with acceptor and donor substrates shown as sticks in pink and gold, respectively. **b**, Cross-section schematic view of *Gg*ALG12 viewed from the ER luminal side, indicating the arrangement of TMs and the substrate-binding pockets. The conserved and variable domains of the GT-C_A_ fold are indicated with gray and purple shading, respectively. The location of the catalytic base side chain is indicated with a magenta sphere. **c**, Structure of ternary complex of *Gg*ALG12-E35Q with bound Dol25-PP-GlcNAc_2_Man_7_ (acceptor) and Dol25-P-Man (donor) substrates. **d**, Structure of the ternary complex of *Hs*ALG9-D82A with bound Dol25-PP-GlcNAc_2_Man_8_ (acceptor) and Dol25-P-Man (donor) substrates. Insets in **c**,**d** show cartoons of bound substrates, with monosaccharides resolved in the cryo-EM density colored according to the Symbol Nomenclature for Glycans, while unresolved monosaccharides are depicted in gray. In **c**,**d**, cryo-EM densities for bound substrates are shown as transparent gray surfaces. Acceptor and donor substrates are depicted in pink and gold stick representation. Key residues are highlighted as sticks and hydrogen-bonding interactions are shown as dashed lines.

**Extended Data Fig. 4:**
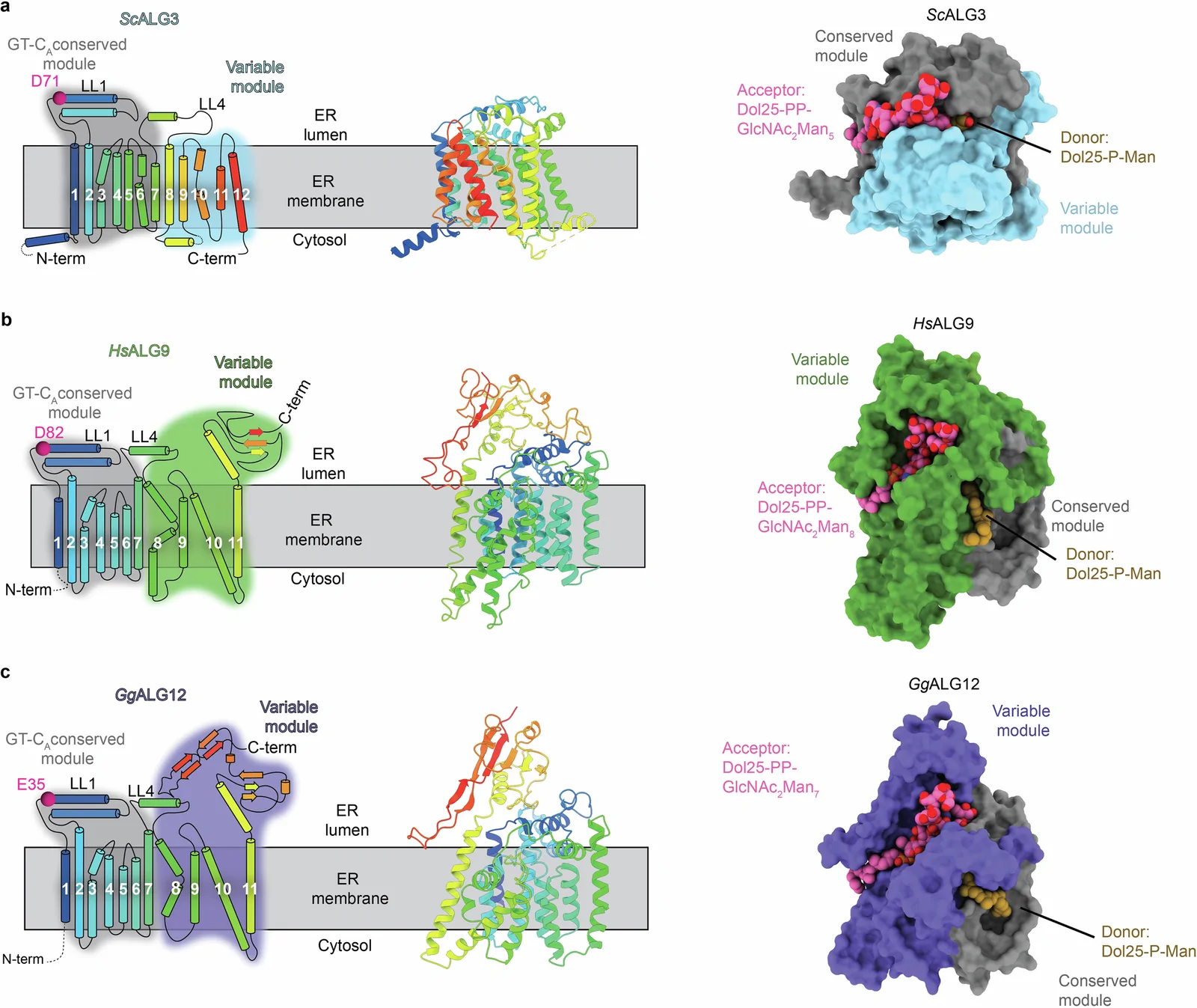
ER luminal mannosyltransferase architecture. Topology, cartoons in rainbow colouring and surface representation for *Sc*ALG3 **(a)**, *Hs*ALG9 **(b)** and *Gg*ALG12 **(c)**. The GT-C_A_ conserved module is shown in grey shading while the variable module is shaded in colour. The surface representations show the acceptor substrate binding cavity forms at the interface of the GT-C_A_ variable and conserved modules in CAZy families GT-22 (ALG9 and ALG12) and GT-58 (ALG3). The acceptor and donor substrates are shown as pink or gold spheres, respectively.

Previous phylogenetic studies have suggested that enzymes involved in *N*-linked glycan or glycosylphosphatidylinositol anchor synthesis and using Dol-P-Man as a donor substrate have a common ancestor, with each enzyme arising through gene duplication and divergent evolution^43^. In line with this hypothesis, our structures revealed that *Sc*ALG3, *Hs*ALG9 and *Gg*ALG12 feature similar folds and belong to the GT-C_A_ class (Figs. 2b,c and 3b and Extended Data Fig. 4). The GT-C_A_ class includes integral membrane enzymes that use isoprenoid-bound sugars as donor substrates and feature an N-terminal conserved module consisting of seven transmembrane (TM) helices, followed by a C-terminal module of varied length and structure^41^. The first luminal loop (LL1), located within the conserved domain, comprises two α-helices, the first of which contains the catalytic base. The variable module of *Sc*ALG3 is composed of five TM helices, whereas *Hs*ALG9 and *Gg*ALG12 feature only four TM helices with a C-terminal domain that extends into the ER lumen (Figs. 2b,c and 3b and Extended Data Fig. 4).

ALG3 is classified within the GT-58 family according to the CAZy nomenclature^44^. It contains 12 TM helices, with cytosolic N and C termini, and extends only minimally outside the membrane. ALG9 and ALG12 are classified as GT-22 enzymes. They contain 11 TM helices and a cytosolic N terminus. *N-*glycosylation of *Sc*ALG3 or *Gg*ALG12 was not observed but the variable domain of *Hs*ALG9 is *N-*glycosylated at N593, as indicated by cryo-EM density for the first GlcNAc entity of an *N*-glycan.

### Structural basis of B-branch initiation

To capture the ternary complex representing initiation of the B branch, detergent-solubilized *Sc*ALG3 was mixed with chemoenzymatically synthesized acceptor and donor substrates (Dol25-PP-GlcNAc_2_Man_5_ and Dol25-P-Man, respectively) before cryo-EM grid vitrification. The resulting structure revealed the acceptor and donor substrate-binding pockets, which flank the catalytic cleft on the luminal face of ALG3 (Fig. 2a,b,d). The acceptor Dol25 moiety is located in a hydrophobic groove formed by TM1 and TM3, where it is exposed to the lipid bilayer (Fig. 2b,d). An electropositive patch formed by R163 and R171 of TM3 is located at the membrane boundary, facing the ER lumen and forming ionic interactions with the pyrophosphate moiety. The two GlcNAc entities are bound to the protein surface adjacent to the catalytic cleft, positioning the **c** mannose such that the A branch projects toward bulk solvent, whereas the **e** mannose reaches into the active site (Fig. 2d,e). The A branch is likely mobile because its cryo-EM density weakens as it extends away from the protein surface. Strong CH–*π* contacts between the **b** GlcNAc and Y410, as well between the **c** mannose and W286, help position the substrate for catalysis (Supplementary Fig. 4a). Similar CH–*π* interactions have been shown to have an important role in other carbohydrate–protein interactions^45,46^. Within the active site, a hydrogen bond between the **e** mannose 4-OH and the side-chain nitrogen of Q373 helps orient the 3-OH for attack on the anomeric carbon atom of the donor substrate (Fig. 2e). The side chain of the catalytic D71, here substituted to asparagine, forms a hydrogen bond to the C3-OH of the **e** mannose, supporting its role as the catalytic base. The distance between the attacking C3-OH of the acceptor and the donor anomeric carbon is ~3.2 Å. Our structure, therefore, represents a pseudo-Michaelis state primed for mannose transfer and the formation of the α1,3-linkage.

The Dol25-P-Man donor substrate enters the active site from the opposite side of *Sc*ALG3 relative to the acceptor substrate pyrophosphate group. The fourth luminal loop (LL4) forms a bridge over the donor substrate-binding groove, an architectural feature that was previously observed for GT-C_A_ enzymes including ALG6, ArnT, EmbA-C and AftD^35,47–49^ (Extended Data Fig. 4a). We observed cryo-EM density for the entire Dol25-P-Man molecule, which revealed that the Dol25 moiety interacts with TM6 and TM8 (Fig. 2b). The phosphate group interacts with R280 from LL4, with H371 from LL5 connecting TM9 and TM10, and with the hydroxyl group of Y75 through a water molecule (Fig. 2e). Dol25-P-Man adopts a bent-back conformation, with the axial C2-OH projecting away from the acceptor substrate (Fig. 2e). Bent-back conformations have been observed for the Dol-P-Man donor substrate in the *C*-mannosyltransferase DPY19 (ref. ^50^) and in several structures containing nucleotide-activated donor sugars^51–56^. While energetically less favorable, the bent-back conformation is thought to prime the anomeric carbon for attack by the incoming nucleophile of the enzyme-bound acceptor substrate and subsequent release of the leaving group, Dol-P.

### Ternary complexes reveal completion of B and C branches

To uncover how ALG9 completes the B and C branches with the addition of α1,2-linked mannoses, we collected cryo-EM data of nanodisc-reconstituted enzyme in the presence of Dol25-P-Man donor substrate and either Dol25-PP-GlcNAc_2_Man_6_ (containing an initiated B branch) or Dol25-PP-GlcNAc_2_Man_8_ (containing a completed B branch and an initiated C branch) acceptor substrates (Figs. 2a and 3a). Our structures reveal how these distinct substrates are accommodated in the active site of the enzyme (Figs. 2a,c,f and 3a,d). In both cases, the acceptor substrate pyrophosphate group binds to a cluster of electropositive residues (R428 and R577) at the ER luminal membrane boundary while the Dol25 moiety binds to a hydrophobic patch formed by TM8 and TM11. The *N-*acetyl group of the **a** GlcNAc points out of the active-site cleft, whereas, in *Sc*ALG3 and *Gg*ALG12, the GlcNAc sugars are rotated 180° (Figs. 2d,f and 3c,d). A hydrogen bond between the **a** GlcNAc *N-*acetyl group and the side-chain nitrogen of N309 and a CH–*π* interaction between the Y473 side chain and the **c** mannose anchor the acceptor substrates (Supplementary Fig. 4b). As with the *Sc*ALG3 ternary structure, the A branch of bound acceptor LLO projects out of the catalytic cleft. The catalytic base, D82, was substituted to alanine to capture these ternary complexes. The ALG9 structures show the Cβ carbon of A82 within 5 Å of the C2-OH of the acceptor mannose, supporting the notion that the acceptor substrate represents a pretransfer pose.

In both ternary structures of *Hs*ALG9, we observed strong cryo-EM density for the Dol25 and phosphate moieties of bound Dol25-P-Man, which was bound in a hydrophobic groove formed by TM6 and TM9 and in a tunnel formed by LL4. The phosphate group interacts with two arginine residues, R112 and R370, which likely facilitate the transfer reaction through stabilization of the leaving group (Figs. 2f and 3d). In contrast to the *Sc*ALG3 structure, cryo-EM density for the mannose of bound Dol25-P-Man was not observed in *Hs*ALG9. To exclude that futile donor hydrolysis was responsible, we carried out activity assays with substoichiometric amounts of substrate relative to enzyme (Methods) and found no evidence of donor substrate hydrolysis (Supplementary Fig. 5a). This suggested that the donor substrate was chemically intact in our cryo-EM samples but that the mannose group was too mobile to allow visualization. We further investigated this conclusion using MD simulations, as discussed below.

### Structural basis of C-branch initiation

The structure of *Gg*ALG12 was determined in the presence of the chemoenzymatically generated acceptor substrate Dol25-PP-GlcNAc_2_Man_7_ and the donor substrate Dol25-P-Man. The Dol25 moiety of the acceptor is lodged in a groove formed by TM8 and TM11, facing the lipid bilayer (Fig. 3b). The pyrophosphate group is coordinated by H140 of TM3 and R271 of TM8 and located at the ER luminal membrane boundary (Fig. 3c). The side-chain nitrogen of N370 interacts with the C3-OH of the **a** GlcNAc, positioning the **c** mannose of the glycan such that the **e** mannose, to which the B and C branches are connected, projects toward the active site, while the A branch points away from the active site and interacts with the luminal domain of ALG12. The **c** mannose is further stabilized by a CH–*π* stacking interaction to the side chain of W256 (Supplementary Fig. 4c). ALG12 catalyzes an α1,6-linked mannose addition and we indeed found the C6-OH of the oligosaccharide e mannose at a distance of ~3.6 Å from the ε-oxygen of the catalytic base, E35, here substituted to glutamine. Notably, the acceptor B branch projects into a cavity created by LL1 and the C-terminal luminal domain of ALG12, where it interacts with several residues lining the pocket.

The donor substrate, Dol25-P-Man, accesses the active site through a tunnel formed by TM6, TM9 and LL4 (Fig. 3b). The phosphate group is coordinated by the side chains of R67 from LL1, R185 from the loop between TM5 and TM6 and R312 located in the loop between TM9 and TM10. Given this coordination of the leaving group phosphate, we believe this structure captures the state before mannose transfer. As with the *Hs*ALG9 ternary complexes, density for the donor mannose sugar was not observed despite the use of the catalytic mutant and the addition of Dol25-P-Man before vitrification. We, therefore, performed a similar assay as detailed for ALG9 to detect donor hydrolysis but none was observed (Supplementary Fig. 5b).

### Molecular logic of oligomannose core assembly

The correct composition and structure of the GlcNAc_2_Man_9_Glc_3_ glycan transferred to nascent glycoproteins is essential for quality control mechanisms in the ER and for the generation of hybrid and complex glycans in the Golgi. In vivo studies have shown that ALG3, ALG9 and ALG12 synthesize the *N-*glycan in a defined order^20,22–24^. Our structural and functional data reveal the basis for ordered assembly of the mannose core.

**Fig. 4:**
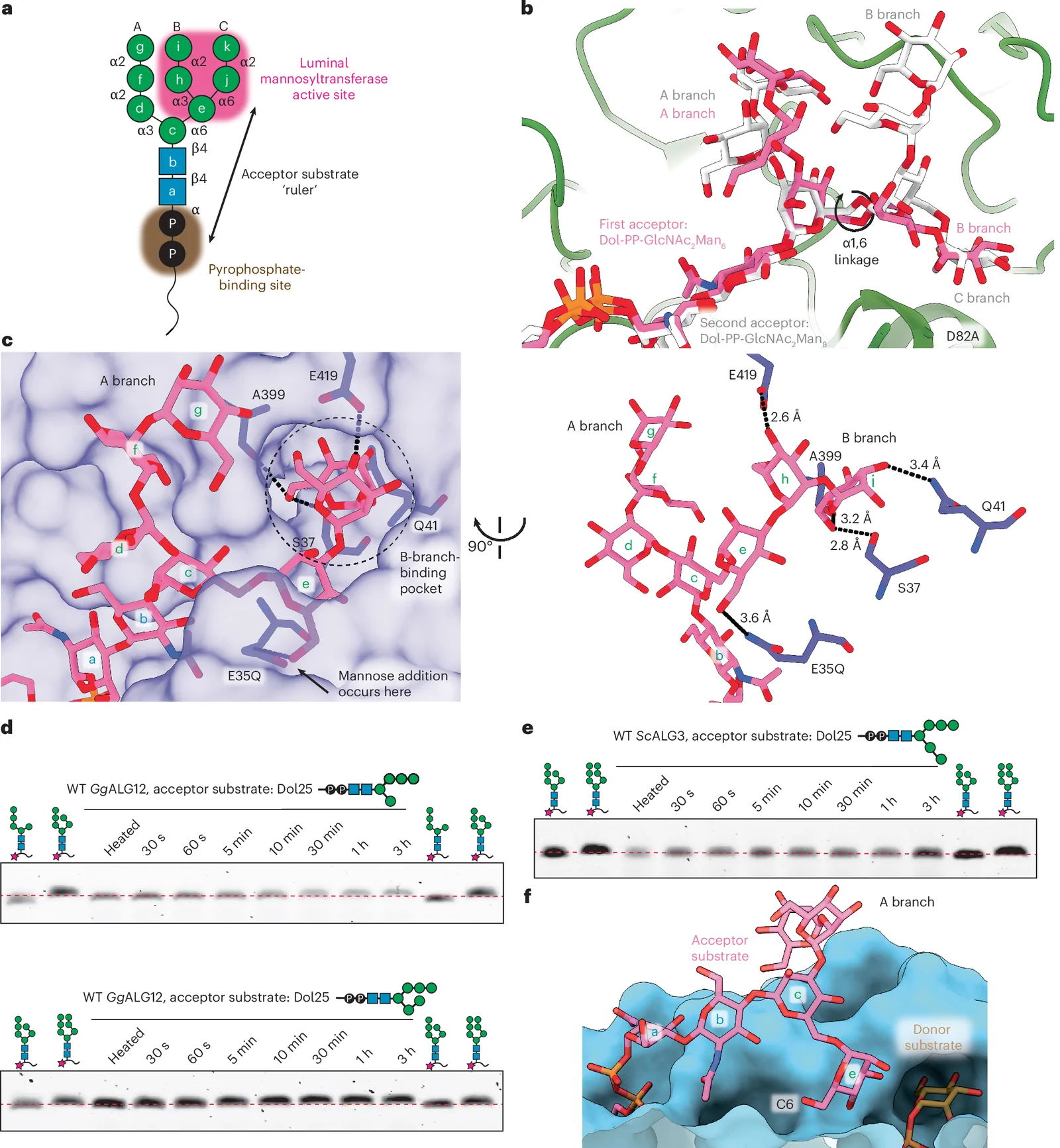
Molecular basis of ordered oligomannose synthesis. **a**, The distance between the pyrophosphate-binding site of ALG enzymes and the active sites acts as a molecular ruler to select the correct acceptor substrate. **b**, Superimposed structures of *Hs*ALG9 containing either Dol25-PP-GlcNAc_2_Man_6_ (pink sticks) or Dol25-PP-GlcNAc_2_Man_8_ (white sticks) substrates reveal two distinct binding modes. **c**, Acceptor B-branch-binding pocket in *Gg*ALG12 structure underpins the requirement of a completed B branch in the acceptor substrate. **d**, Activity assays with *Gg*ALG12 and Dol25-PP-GlcNAc_2_Man_5_ (no B branch) or Dol25-PP-GlcNAc_2_Man_6_ (initiated but incomplete B branch) acceptor substrates. *Gg*ALG12 processes substrates without a complete B branch very slowly compared to the native acceptor substrate. **e**, Functional studies of *Sc*ALG3 with Dol25-PP-GlcNAc_2_Man_6_ bearing an initiated C branch showed no turnover even after extended incubation. In **d**,**e**, reactions were incubated as indicated above the lanes; for ‘heated’ samples, the enzyme was replaced with heat-inactivated enzyme. The obtained glycans were transferred to a fluorescent peptide (TAMRA-YA**N**ATS-NH_2_) using *Tb*STT3B and separated on a tricine gel. Lanes with glycopeptide cartoons over them serve as glycopeptide standards to allow comparison. Time-course assays in **d**,**e** were performed at least twice with similar results. A schematic for reactions in **d**,**e** is shown in Extended Data Fig. 5d. **f**, Zoomed-in view of *Sc*ALG3 catalytic cleft with bound donor and acceptor substrates. The ternary complex shows insufficient room in the active-site cleft to accommodate an initiated acceptor C branch at the indicated C6 hydroxyl. Source data

**Extended Data Fig. 5:**
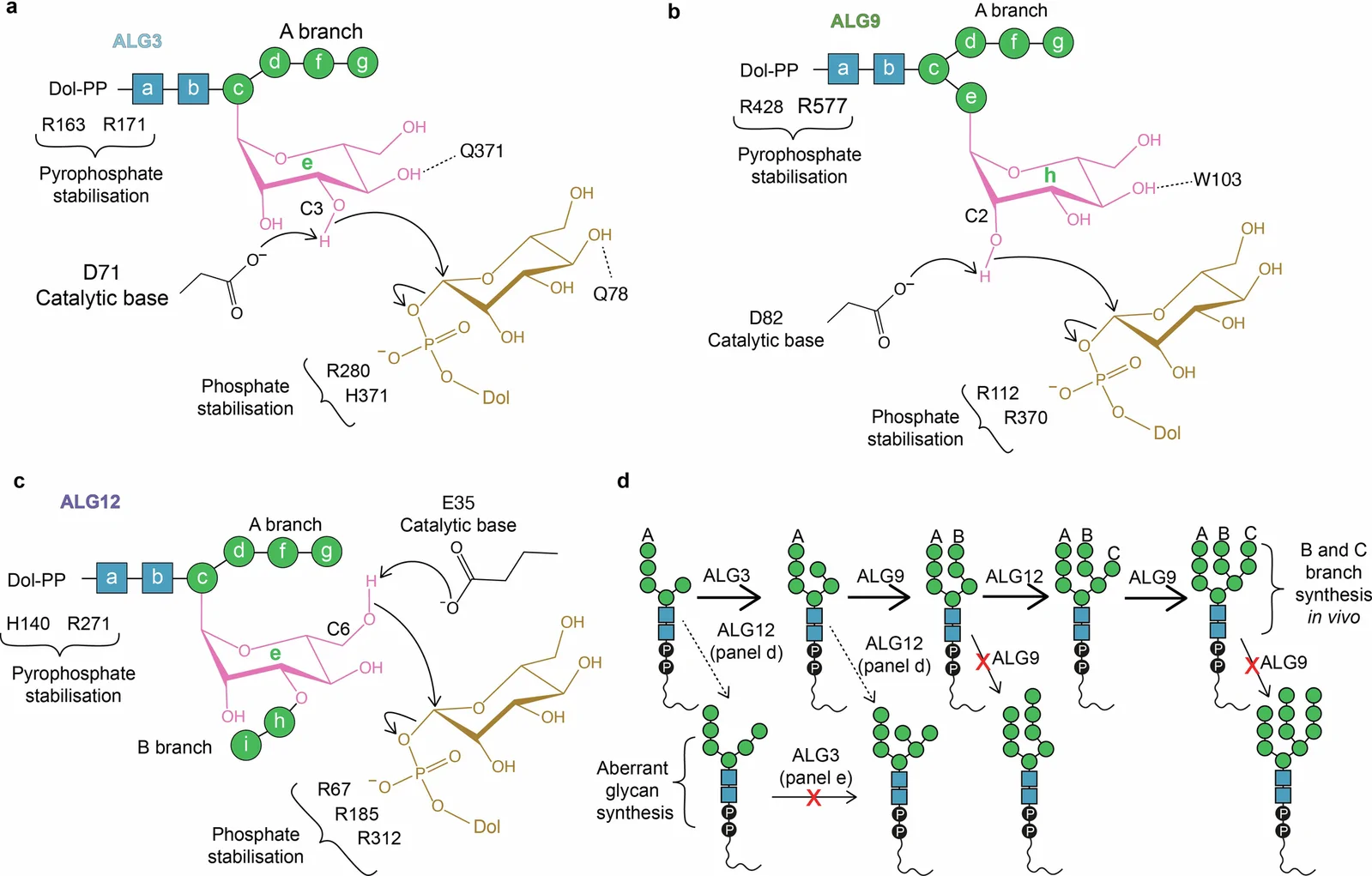
Catalytic mechanisms of ALG3, ALG9 and ALG12 and schematic of *N*-glycan assembly. Proposed reaction mechanisms for **a**, B branch initiation by ALG3, **b**, B branch completion by ALG9 and **c**, C branch initiation by ALG12. Monosaccharides are labelled according to the nomenclature in Fig. 1a. The acceptor carbon bound to the attacking hydroxyl is indicated in each panel. **d**, Schematic of eukaryotic oligomannose *N*-glycan assembly. Bold arrows indicate *in vivo* reactions, while dashed arrows indicate slow reactions that can be forced *in vitro*. Reactions that do not occur are indicated with a red cross.

The binding pockets of ALG3, ALG9 and ALG12 are precisely tailored to allow accommodation of acceptor glycans in an extended pose from the pyrophosphate-binding site to the active site (Figs. 2d,f and 3c,d). This suggests that the distance from the pyrophosphate-binding site to the active site functions as a molecular ruler, preventing mannose transfer to incorrect acceptor substrates (Fig. 4a). The observed interactions between the acceptor pyrophosphate and the protein constrain the substrate and enforce a binding pose that presents the correct hydroxyl for activation by the catalytic base. Such activity control likely prevents ER luminal mannosyltransferases from acting as polymerases (Extended Data Fig. 5d), which would result in overly long B and C branches. An analogous ruler concept was suggested for the bacterial glycosyltransferase PglH of *Campylobacter* *jejuni*^57^.

The two *Hs*ALG9 ternary complexes illuminate how two different acceptor substrates are accommodated by a single enzyme. The dual specificity relies on rotation of the α1,6-linkage between the **c** and **e** mannoses, allowing presentation of either the **h** or the **j** mannose to the active site (Fig. 4b). For the first acceptor substrate (Dol-PP-GlcNAc_2_Man_6_), the C branch has not been initiated; thus, only the B-branch-initiating mannose of the acceptor substrate is able to reach the active site. For the second acceptor substrate (Dol-PP-GlcNAc_2_Man_8_), the B branch has been already completed and projects toward bulk solvent.

Whereas ALG9 evidently requires ALG3 or ALG12 to initiate the B and C branches before they can be completed, it was less obvious how ALG12 activity was prevented until ALG3 and ALG9 could complete the B branch. The physiological importance is that a GlcNAc_2_Man_7_ intermediate containing **h** and **j** mannoses must be avoided by the biosynthetic pathway because it could interfere with protein quality assessment processes mediated by the lectin OS9, which depends on such a GlcNAc_2_Man_7_ structure^58^. The ALG12 ternary structure reveals that this is implemented by a deep binding cavity that accommodates the B branch of the acceptor glycan (Fig. 4c). The **h** mannose interacts with the side chain of E419 through its C4-OH, while the **i** mannose forms hydrogen bonds to the side chains of S37 and Q41, as well as to the backbone carbonyl of A399 (Fig. 4c). Tight binding and immobilization of the completed B branch in ALG12 likely helps orient the acceptor **e** mannose to receive the α1,6-linked j mannose, providing a mechanism to strongly favor the GlcNAc_2_Man_7_ glycan and prevent premature C-branch initiation. Assays with wild-type (WT) *Gg*ALG12 and acceptor substrates either lacking a B branch or featuring a B branch containing a single mannose demonstrated that mannosyl transfer activity was drastically reduced but not impossible, in line with in vivo observations^24^ (Fig. 4d and Extended Data Fig. 5d). Furthermore, yeast cells lacking ALG9 accumulate a GlcNAc_2_Man_6_ intermediate through ALG3 activity, providing evidence that ALG12 does not proceed with C-branch initiation unless the B branch is first completed^24^. Only with overexpression of ALG12 do *ALG9*-knockout cells produce a certain amount of GlcNAc_2_Man_7_ (ref. ^24^). Should ALG12 occasionally generate a nonnative Dol-PP-GlcNAc_2_Man_6_ intermediate containing an initiated C branch, this acceptor substrate is not processed by ALG3 (Fig. 4e and Extended Data Fig. 5d). The *Sc*ALG3 ternary complex structure revealed that an acceptor substrate with an initiated C branch would cause a steric clash in the binding pocket (Fig. 4f). This ensures that no GlcNAc_2_Man_7_ intermediates containing initiated B and C branches accumulate.

Our ALG3 structure revealed that the A-branch mannoses project away from the enzyme toward the bulk solvent (Figs. 2d and 4f) and are not required for activity. Hence, ALG3 does not ensure completeness of the GlcNAc_2_Man_5_ moiety. However, there is evidence that the flippase RFT1 favors Dol-PP-GlcNAc_2_Man_5_ over Dol-PP-GlcNAc_2_Man_3_, which may prevent immature glycans from being flipped to the ER lumen^18^. The OST complex has a specific pocket for the Glc_3_ moiety of the oligosaccharide but the B and C branches are not recognized^29^. Following glycan transfer and the removal of the two terminal glucoses, the remaining glucose is cleaved or reattached, depending on the folding status of the linked protein, during the folding process based on calnexin/calreticulin and UDP-Glc:glycoprotein glucosyltransferase (UGGT)^59^. However, the glucosidases and UGGT require a fully mannosylated glycan with complete B and C branches for efficient removal or addition of glucose and, therefore, for proper functioning of this pathway^60,61^. Given the importance of the mannoses in these branches for subsequent signaling steps in the ER and Golgi, it is essential that the B and C branches are completed before the glucosyltransferases complete the A branch to prevent transfer of a premature glycan. In vivo evidence suggests that one or more of the ER luminal glucosyltransferases provide this specificity^23,62,63^.

### Catalytic mechanism

Our structural and functional studies define the geometry of the active site for *Sc*ALG3, *Hs*ALG9 and *Gg*ALG12 and allow us to propose structure-based reaction mechanisms (Extended Data Fig. 5a–c). The three mannosyltransferases facilitate an inverting mechanism by accepting a β-linked donor mannose and transferring it to the acceptor LLO with an α-linkage (α1,3 for ALG3, α1,2 for ALG9 and α1,6 for ALG12). We propose a bimolecular nucleophilic substitution reaction, whereby the attacking hydroxyl group of the acceptor mannose is activated by deprotonation by a catalytic base (side chains of D71 in *Sc*ALG3, D82 in *Hs*ALG9 and E35 in *Gg*ALG12). This is followed by an attack of the donor mannose anomeric carbon, causing inversion of configuration and departure of the Dol-P leaving group. In each enzyme, electropositive residues coordinate the donor phosphate, thus stabilizing the negative charge of the leaving group. Notably, the acceptor substrates bind ALG3, ALG9 and ALG12 at the domain boundary of the conserved and variable module, with specific contacts predominantly formed with residues of the variable module (Figs. 2b,c and 3b and Extended Data Fig. 4). We hypothesize that the GT-C_A_ variable modules also facilitate the specific binding of diverse acceptor substrates in other GT-C_A_ enzymes.

### Mechanism of donor substrate specificity

**Extended Data Fig. 6:**
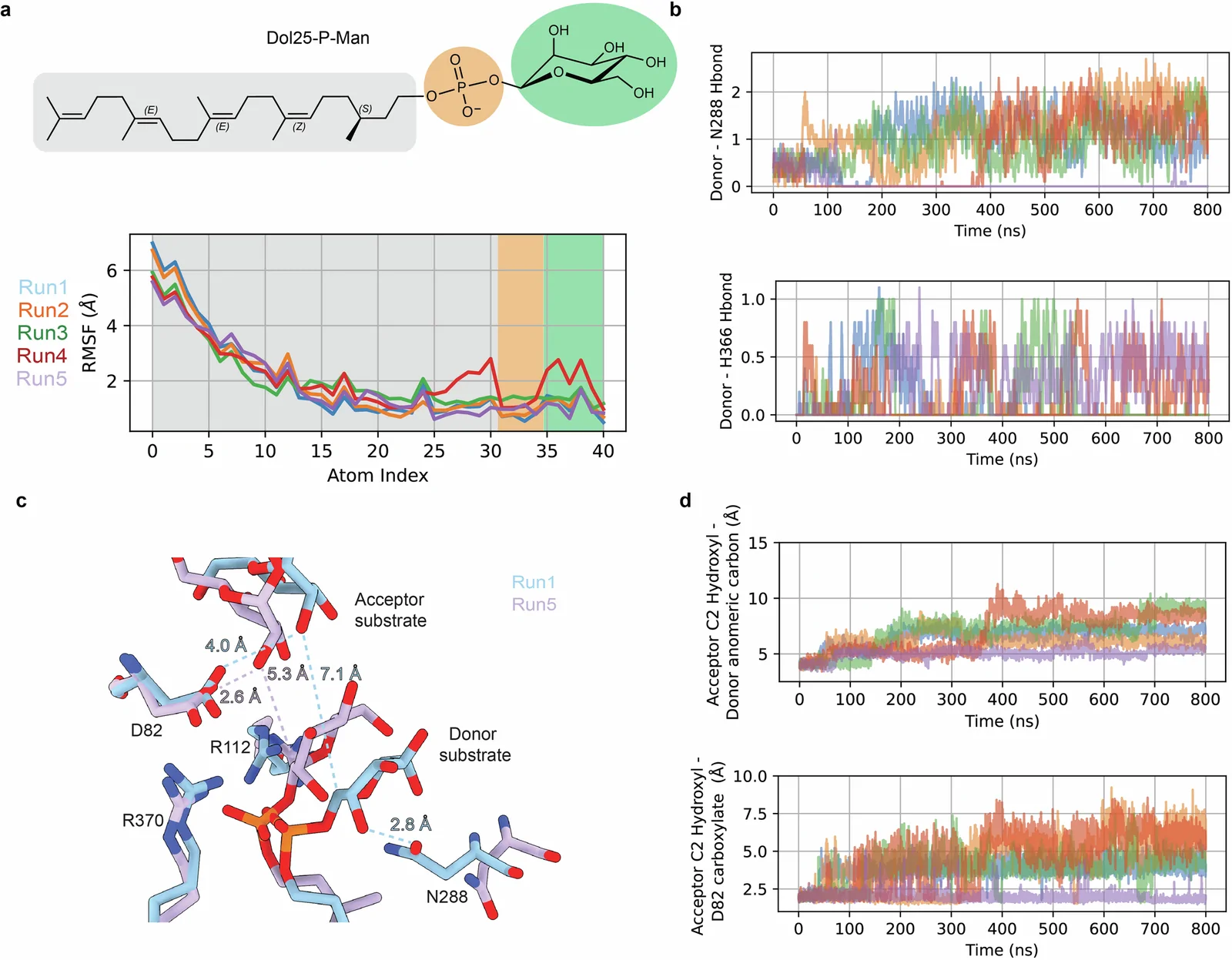
Molecular dynamics (MD) simulations of *Hs*ALG9 with Dol25-PP-GlcNAc_2_Man_8_ acceptor and Dol25-P-Man donor substrates. **a**, Structure of donor substrate Dol25-P-Man, with the hydrophobic tail highlighted in grey, the phosphate group in orange, and the mannose in light green. Root mean square fluctuation (RMSF) of the donor substrate atoms was calculated by aligning all MD frames to the averaged conformation. **b**, Plots of hydrogen bond formation between the side chains of Asn288 (upper panel) or His366 (lower panel) and the donor substrate C2-OH over the duration of 5 simulations. Data are shown in running average of 1 ns for the clearer visibility. **c**, Comparison of the active site geometry between the representative frames of the first simulation (blue curve, t = 796.4 ns) and the fifth simulation (purple curve, t = 780 ns). Dol25-P-Man and selected side chains are shown in sticks and labelled. Selected interatomic distances are indicated by the dashed lines with colours corresponding to the respective simulations. **d**, Distance between the acceptor C2 hydroxyl oxygen and donor anomeric carbon (upper panel) and between the acceptor C2 hydroxyl hydrogen and D82 γ-carbon over the course of the simulation. Results of the five independently generated trajectories are shown by different colours as indicated in panel **a**. The last frame of the fifth (purple curve) trajectory was taken as the initial template for the subsequent simulations. MD simulations were performed with 5 replicates.

The donor substrates Dol-P-Man and Dol-P-Glc are both present in the ER membrane, making it essential for ER luminal mannosyltransferase and glucosyltransferase enzymes to discriminate between these C2 epimers. To explore the molecular basis for donor selectivity, we conducted MD simulations with *Hs*ALG9 using the cryo-EM structure of the second ternary complex (capturing the state just before completion of the C branch) as the starting model. In a first set of five replicates over 800 ns, we observed that, while the lipid tail of Dol25-P-Man was highly mobile, the phosphate and sugar moieties were more stably bound (Extended Data Fig. 6a). During the simulations, the axial C2-OH of the donor mannose often formed hydrogen bonds with the side chains of N288 and H366, suggesting that these residues might have a role in donor selection (Extended Data Fig. 6b). However, the Dol25-P-Man molecule shifted further into the active site by about 2 Å in one of the five simulations, with the side chains of R112 and R370 moving apart to accommodate this motion and better coordinate the donor phosphate leaving group (Extended Data Fig. 6c,d). In this new ‘engaged’ pose, the distance between the C2-OH of the acceptor mannose and the anomeric carbon of the donor mannose was shortened, suggesting that this represented a conformation closer to the transition state.

**Fig. 5:**
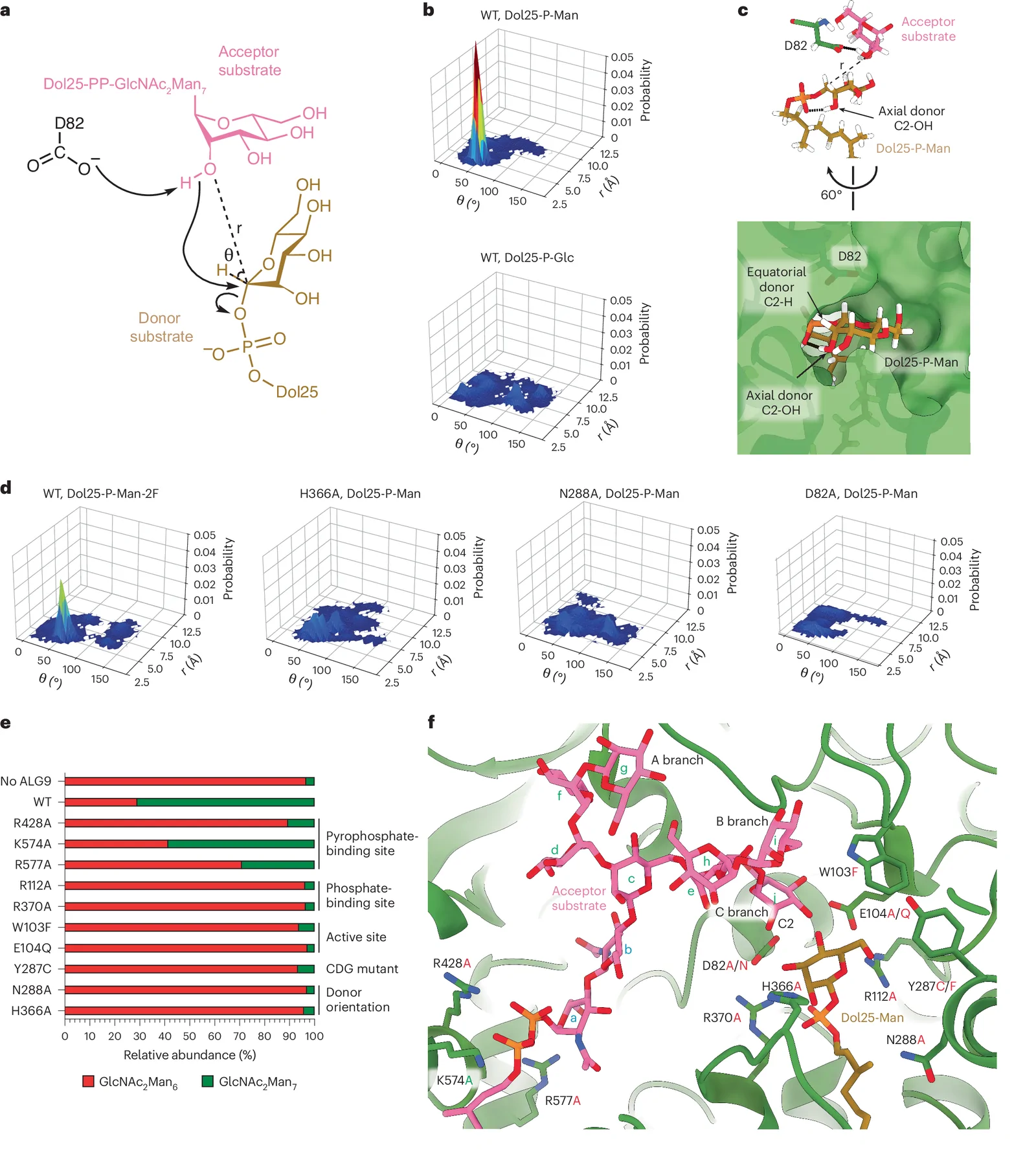
MD simulations and mutagenesis of *Hs*ALG9. **a**, Definition of donor pose metrics used for analysis of MD results including distance (*r*) and angle (θ). **b**, Probability of different distance (*r*) and angle (θ) values observed during MD simulations for the donor analogs, Dol25-P-Man or Dol25-P-Glc. In **b**,**d**, the plots are colored by the relative probability of observed donor poses. **c**, Representative pose of Dol25-P-Man in the catalytically favorable conformation in the active site of WT *Hs*ALG9. Top, hydrogen bonds between the attacking hydroxyl of the acceptor substrate and the catalytic base (D82) and between the donor C2-OH and the phosphate oxygen are shown with dotted lines. The distance (*r*) between the acceptor C2 hydroxyl oxygen and donor anomeric carbon is indicated with a dashed line. Bottom, Dol25-P-Man shown as sticks in the active site, with *Hs*ALG9 displayed as a partially transparent surface. **d**, Probability density of different distance (*r*) and angle (θ) values observed during MD simulations with WT *Hs*ALG9 and Dol25-P-Man-2F or with *Hs*ALG9 mutants and Dol25-P-Man. **e**, Relative amount of GlcNAc_2_Man_6_ (acceptor) and GlcNAc_2_Man_7_ (product) after reaction for 30 s with different *Hs*ALG9 mutants. The resulting glycans were transferred to excess fluorescent peptide (TAMRA-YANATS-HN_2_) with *Tb*STT3B and measured using high-resolution MS. **f**, Structure of *Hs*ALG9 active site, with protein residues colored green, acceptor substrate colored pink and donor substrate colored gold. The shown structure represents an initial frame of an MD simulation with WT *Hs*ALG9 and Dol25-P-Man. Key residues are shown as sticks and labeled, including the substitutions introduced. The color of the label (red, deleterious; green, minimal effect) indicates the effect of the substitution on ALG9 mannosyltransferase activity. Source data

**Extended Data Fig. 7:**
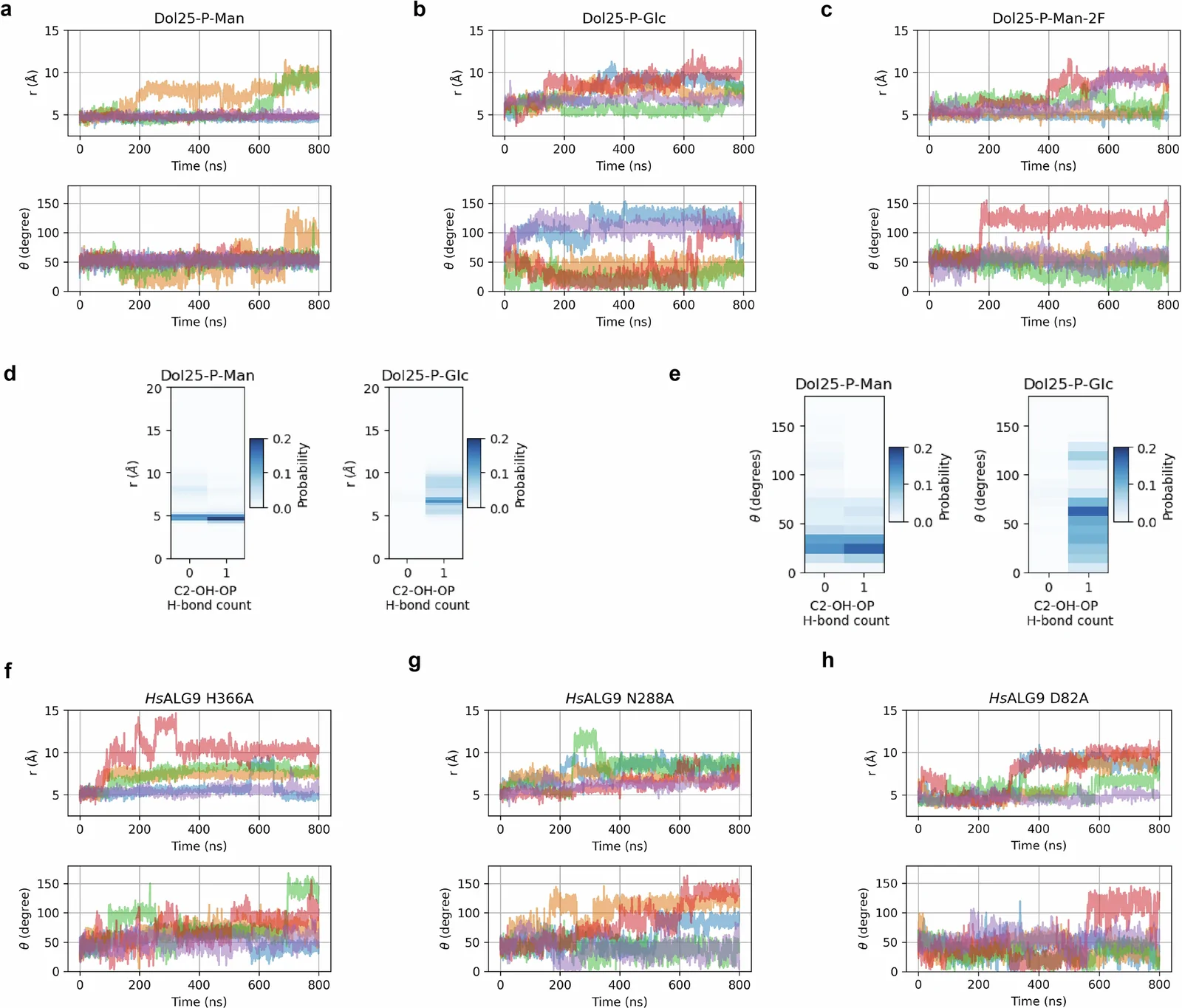
Time plots of molecular dynamics (MD) simulations of WT or mutant *Hs*ALG9. **a-c**, Donor distance (r) and angle (θ) in the simulations of wild type *Hs*ALG9 in complex with acceptor substrate and donor analogues, as defined in Fig. 5a. Shown are the plots for Dol25-P-Man **(a)**, Dol25-P-Glc **(b)**, or Dol25-P-Man-2F **(c)** over the course of the simulation. **d,e** Plot of probability for distance (r) **(d)** and angle (θ) **(e)** values observed during MD simulations in panels **a** and **b**, here filtered by the presence or absence of the intramolecular hydrogen bond between the donor C2-OH and the donor phosphate. Here a hydrogen bond is defined by an acceptor-hydrogen-donor angle cutoff of less than 135° and a donor-acceptor distance of less than 3 Å. **f-h** Distance (r) and angle (θ) in the simulations of *Hs*ALG9 mutant N288A (**f**), H366A (**g**) or D82A (**h**) in complex with acceptor substrate and Dol25-P-Man. In each example, MD simulations were performed with 5 replicates.

To test the relevance of these simulated donor sugar–protein interactions, we conducted further MD simulations using the engaged Dol25-P-Man pose as the starting structure. We first compared distinct donor molecule analogs and performed simulations with Dol25-P-Man, Dol25-P-Glc or Dol25-P-Man-2F, where the C2-OH group was replaced by a fluorine atom, which cannot serve as a hydrogen-bond donor (Fig. 5a–d and Extended Data Fig. 7a–c). We plotted the relative propensity of different donor sugar poses (‘probability’) during the MD simulations, defined by the observed distances between the donor anomeric carbon and the attacking acceptor oxygen and the angle between the C–H bond of the anomeric carbon and a line between the anomeric carbon and the acceptor C2 oxygen (Fig. 5a). The simulations showed that Dol25-P-Man predominantly adopted a catalytically favorable pose, whereas Dol25-P-Glc rarely adopted a similar pose (Fig. 5b,c and Extended Data Fig. 7a,b). This is likely because, in the engaged pose, the donor mannose C2 is within van der Waals distance of the enzyme and space exists only for an axial hydroxyl group (Fig. 5c). An equatorial hydroxyl group, such as that in Dol-P-Glc, would cause a steric clash, offering a molecular explanation for the donor substrate specificity. When simulating with the nonreactive donor analog Dol25-P-Man-2F, a maximum was observed in a similar position to the Dol25-P-Man simulation (Fig. 5b,d and Extended Data Fig. 7a,c). However, the peak was lower, suggesting that the fluorinated Dol-P-Man analog not only is inactive but may also be less likely to adopt a potentially reactive pose in the active site.

The simulations also revealed an intramolecular hydrogen bond between the mannose C2-OH and the phosphate group of Dol25-P-Man, resulting in a bent-back pose similar to that observed in the experimental *Sc*ALG3 structure (Figs. 2d,e and 5c). Notably, we observed that a loss of this hydrogen bond correlated with a loss of the engaged donor pose (Extended Data Fig. 7d,e). The simulations with Dol25-P-Glc revealed an analogous hydrogen bond between the C2-OH and the phosphate oxygen but a catalytically active pose was not favored (Fig. 5b and Extended Data Fig. 7d,e). Dol25-P-Man-2F does not permit similar hydrogen bonding because of the fluorine substitution. Our results suggests that, while Dol-P-Glc can enter the active site of ALG9 in vivo, it cannot proceed to adopt a catalytically active pose.

The hydrogen-bonding interactions between the donor mannose C2-OH and the side chain of N288 are eliminated in the engaged pose. However, N288 may have a role in orientating the donor substrate as it moves into the active site. To probe the role of N288 and H366, we performed additional MD simulations of *Hs*ALG9 after replacing these residues individually with alanine. The resulting Dol25-P-Man pose distributions were analogous to those with Dol25-P-Glc, supporting the role of these two residues in correctly orientating the donor substrate (Fig. 5b,d and Extended Data Fig. 7f,g). Given the lack of density for the donor mannose in our ALG9 cryo-EM structures, we also investigated donor mannose mobility by simulating the components vitrified during cryo-EM grid preparation. We carried out MD simulations of *Hs*ALG9-D82A with bound Dol25-P-Man and Dol25-PP-GlcNAc_2_Man_8_ and observed that the donor sugar was highly mobile (Fig. 5d and Extended Data Fig. 7h). This may be because of the added space or lack of charge in the active site as a result of the aspartate to alanine substitution, preventing the donor mannose from adopting a single favored pose.

**Extended Data Fig. 8:**
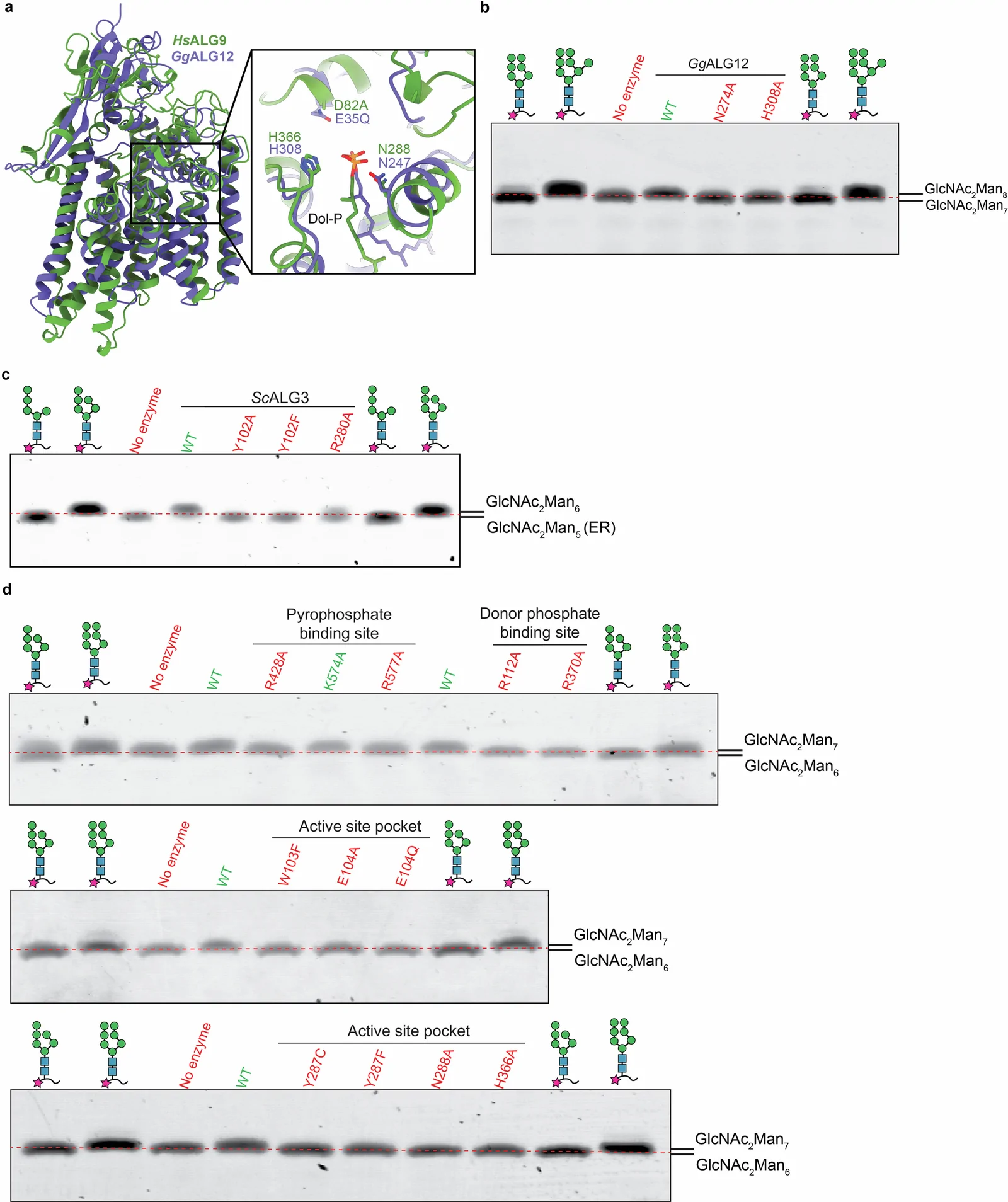
Residues involved in donor substrate selection and catalysis in *Sc*ALG3, *Hs*ALG9 and *Gg*ALG12. **a**, Superposition of *Hs*ALG9 and *Gg*ALG12 structures. The zoomed insert shows the active site, with Dol25-P shown as sticks. **b**, Tricine gel electrophoresis of glycopeptides generated by WT or mutant *Gg*ALG12, with conditions indicated above the lanes. **c**, Tricine gel electrophoresis of glycopeptides generated by WT or mutant *Sc*ALG3 WT, with conditions indicated above the lanes. **d**, Tricine gel electrophoresis of glycopeptides generated using WT or mutant *Hs*ALG9. In **b-d**, following the reaction with *Sc*ALG3, *Hs*ALG9 or *Gg*ALG12, the glycans were transferred to a fluorescently labelled peptide (TAMRA-YA**N**ATS-NH_2_) with *Tb*STT3B. Labels above the gel are coloured to indicate whether mannose transfer has occurred (green) or has not proceeded or is reduced (red). Lanes with glycopeptide cartoons over them serve as glycopeptide standards for the indicated glycan. Activity assays were performed at least three times with similar results. Source data

Given the close structural similarity of *Hs*ALG9 to *Gg*ALG12, we expect analogous mechanisms of donor substrate selection (Extended Data Fig. 8a). *Hs*ALG9 residues N288 and H366 are conserved in *Gg*ALG12 as N247 and H308. Substitution of N247 or H308 to alanine caused a reduction in transfer activity in *Gg*ALG12 as with *Hs*ALG9, as discussed below, supporting the importance of these residues in *Gg*ALG12 (Extended Data Fig. 8b). *Sc*ALG3 also needs to select the correct donor substate but has a clearly distinct architecture compared to ALG9 and ALG12 (Extended Data Fig. 4). Among the residues located within 4 Å of the mannose donor C2-OH, we found Y102 and R280, both of which also coordinate the donor phosphate group (Fig. 2e). The side chains of these residues could form hydrogen bonds with the axial 2-OH of the mannose donor (distance to hydroxyl group of Y102 of 3.7 Å and to η-N of R280 of 2.5 Å). These residues might, therefore, have a role in donor substrate selection in addition to the R280-mediated stabilization of the phosphate leaving group. Functional assays with *Sc*ALG3 variants containing either Y102A, Y102F or R280A substitutions indeed showed reduced transfer activity, supporting the importance of these residues in facilitating mannose transfer (Extended Data Fig. 8c)

### Functional analysis of *Hs*ALG9

To validate our structural-based and simulation-based findings, we expressed and purified *Hs*ALG9 variants, probed their in vitro function (Fig. 5e and Extended Data Fig. 8d) and mapped the observed activities onto the *Hs*ALG9 structure (Fig. 5f). To assay the activity of ALG9 mutants, we transferred the resulting LLOs to a fluorescently labeled peptide and separated them by tricine gel electrophoresis or analyzed the glycopeptide composition by high-resolution mass spectrometry (MS) (Fig. 5e and Supplementary Fig. 6). We found that substitution of arginine residues coordinating the acceptor pyrophosphate (R428A and R577A) reduced ALG9 activity, indicating that these residues have a crucial role in substrate binding. Interestingly *Hs*ALG9 mutants R428A and R577A still had some residual activity, which supports the idea that, while the pyrophosphate binding is necessary, binding is partially supported by either one of these residues. Substitution of K574, which is nearby but does not directly coordinate the pyrophosphate group in either of our structures, did not appear to substantially effect activity. In contrast, substitution of either R112 or R370 appeared to abolish activity supporting their essential function in stabilizing the donor leaving group. The *Hs*ALG9 structures further revealed that E104 forms a salt bridge with R112 in the active site (Figs. 2f and 3d). When the negative charge of this side chain was removed (E104Q or E104A substitutions), ALG9 activity was strongly reduced, suggesting that E104 may be important for correct orientation of R112. Additionally, when W103 was substituted to a phenylalanine, ALG9 activity was again abolished, suggesting that the observed hydrogen bond between the tryptophan side-chain nitrogen and the C4-OH of the acceptor mannose is required for substrate binding or orientation (Figs. 2f and 3d). We also probed the functional role of residues N288 and H366 that were identified in the MD studies. We found that the N288A and H366A variants both abolished *Hs*ALG9 function, supporting their importance for catalysis and validating the MD simulations (Fig. 5d–f and Extended Data Fig. 7f,g).

### Structural basis for CDG involving ALG3, ALG9 and ALG12

**Fig. 6:**
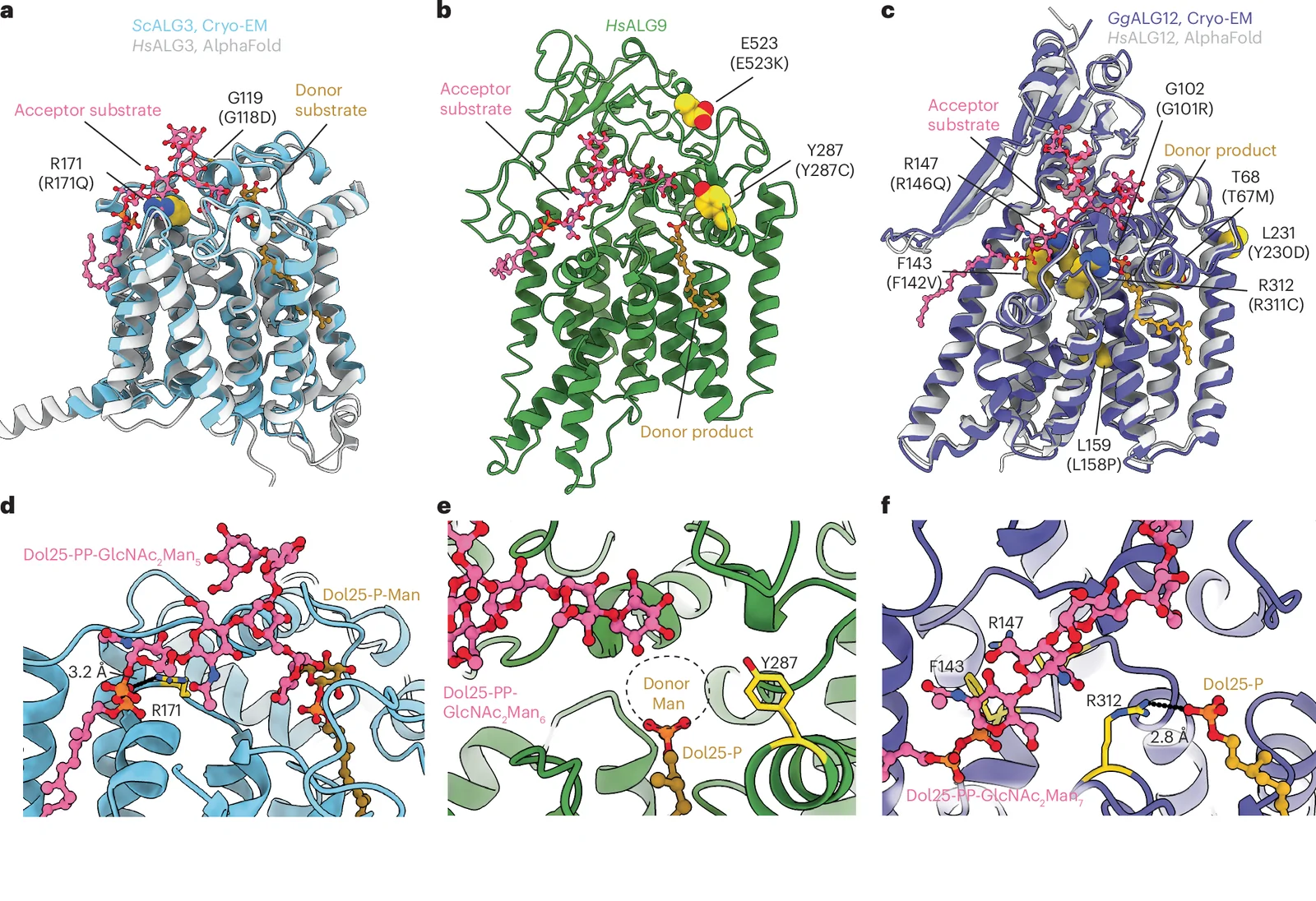
Substitutions in ALG3, ALG9 and ALG12 associated with CDGs in humans. **a**–**c**, Locations of selected substitutions associated with CDGs mapped onto the structures of *Sc*ALG3 (**a**), *Hs*ALG9 (**b**) and *Gg*ALG12 (**c**). Substituted residues are shown as yellow spheres. Equivalent residues in the human constructs are shown in brackets. Superimposed AlphaFold2 models of human homologs of *Sc*ALG3 and *Gg*ALG12 are shown as light-gray cartoons. **d**–**f**, Close-up views of *Sc*ALG3 (**d**), *Hs*ALG9 (**e**) and *Gg*ALG12 (**f**), with selected residues associated with CDGs shown as yellow sticks.

Our structural data provide mechanistic explanations for many of the ALG3, ALG9 and ALG12 variants associated with CDGs in humans (Extended Data Table 2 and Supplementary Figs. 1–3). All highlighted substitutions affect residues that are conserved between the human and yeast homologs of ALG3 and the human and chicken homologs of ALG12 (Fig. 6). Of note, the ALG3-R171Q mutant has been associated with hyperinsulinemic hypoglycemia, hypotonia, facial dysmorphisms and death within the first 3 weeks of life^64^. Our structural data reveal that R171 is involved in an electrostatic interaction with the pyrophosphate moiety of the acceptor substrate, suggesting that substrate recruitment would be disrupted in a R171Q mutant (Fig. 6a,d). In ALG9, the Y287C mutant has been linked to severe symptoms including hypotonia, recurrent seizures and psychomotor retardation^65,66^. Y287 lines the binding site of the donor mannose. We purified the *Hs*ALG9 variants Y287C and Y287F and observed that both showed strongly reduced catalytic activity, suggesting that the side-chain hydroxyl group of Y287 might be indirectly involved in binding and orientating the donor substrate (Figs. 5e,f and 6b,e and Extended Data Fig. 8d). In human ALG12, substitution of R311 (equivalent to R312 in *Gg*ALG12) to cysteine was reported to cause a CDG characterized by psychomotor retardation, severe cerebellar hypoplasia and hypotonia^67^. This residue is in direct vicinity of the donor phosphate group and, therefore, likely stabilizes the leaving group of the substitution reaction (Fig. 6f), which can explain why substitution to cysteine results in a loss of function. Additionally, several persons with CDG displaying debilitating symptoms leading to death in the first 2 years of life were found to have substitutions of either F142 or R146 in human ALG12 (F143 or R147 in *Gg*ALG12)^62,68,69^. These residues form a *π*–cation stacking interaction in our *Gg*ALG12 ternary structure (Fig. 6f) and line the acceptor substrate-binding cleft. They do not directly contact the nearby GlcNAc moieties or the pyrophosphate moiety (closest distance: 4.6 Å away) but this *π*–cation interaction is likely important for the structural integrity of the acceptor substrate-binding cavity.

## Conclusions

Our studies reveal the mechanism and logic of the assembly of the branched oligomannose glycan GlcNAc_2_Man_9_, which underpins essential quality control and folding mechanisms of *N*-glycoproteins in the ER and provides the oligomannose structure required for Golgi glycan remodeling. Furthermore, we show that the discrimination of the correct donor substrate, Dol-P-Man from its C2 epimer, Dol-P-Glc, is mediated by residues within the active site and the adopted pose of the Dol-P-Man donor when it engages in an intramolecular hydrogen bond. In addition to fundamental understanding in an essential biosynthetic pathway, the molecular insights governing LLO substrate selection contribute a basis for future biotechnological engineering, including the production of viral oligomannose epitopes or structure-guided mutagenesis to modify ALG substrate preferences (for example, bump-and-hole methods^70^), facilitating new tools for probing the *N-*linked glycome and glycoproteome. Our results provide a framework for understanding the mechanisms of other GT-C enzymes, particularly those involved in branched-glycan biosynthesis. Lastly, we provide a molecular basis for understanding how ALG3, ALG9 and ALG12 enzyme variants lead to lethal CDGs.

## Methods

### Cloning, expression and purification of *Sc*ALG3, *Hs*ALG9 and *Gg*ALG12

Gene sequences coding for *Sc*ALG3 (UniProt P38179), isoform 1 of *Hs*ALG9 (UniProt Q9H6U8) and *Gg*ALG12 (UniProt F1P077) were codon-optimized for expression in human cells using GeneArt (Thermo Fisher Scientific) and cloned into a modified pUC57 vector using restriction free cloning^71^. *Sc*ALG3 was cloned with a C-terminal HRV 3C cleaveage sequence followed by eYFP and rho-1D4 sequences^72^. *Hs*ALG9 and *Gg*ALG12 where cloned with an N-terminal FLAG tag followed by eYFP and HRV 3C cleavage sequences. All three proteins were transiently expressed in suspension 293 c18 (American Type Culture Collection, CRL-10852) cells maintained at 37 °C in humidified incubators with supplemental carbon dioxide. Protein expression was induced by transient transfection with linear polyethylenimine. Cells expressing protein for 2 days were collected by centrifugation, washed with PBS and flash-frozen before storage at −80 °C.

Cells were thawed in lysis buffer (150 mM sodium chloride, 50 mM HEPES pH 7.5 and 10% (v/v) glycerol) in a 1:5 (w/v) ratio. All subsequent steps were either performed on ice or at 4 °C. Before lysis by Dounce homogenization, 1 mM PMSF, 20 µg ml^−1^ DNAse I (Roche) and a 1:100 (v/v) dilution of protease inhibitor cocktail (Sigma) were added. Membrane extraction was performed for 1 h in 1% (w/v) lauryl maltose neopentyl glycol (LMNG), 0.1% (w/v) cholesteryl hemisuccinate (CHS) or 1% (w/v) *n*-dodecyl-β-D-maltopyranoside (DDM) and 0.2% (w/v) CHS for samples destined for nanodisc reconstitution or substrate synthesis. All detergents used were purchased from Anatrace. The lysate was then centrifuged at 186,000*g* for 30 min in a Type-45Ti (Beckman Coulter) rotor to pellet insolubilized membrane. The supernatant was then incubated with either M2 Flag antibody resin (Sigma) or 1D4 antibody resin (made in house) for 1 h with rotation. The flowthrough was discarded and the resin was washed twice with 15 column volumes of wash buffer (150 mM sodium chloride, 20 mM HEPES pH 7.5, 10% (v/v) glycerol and 0.01% LMNG, 0.001% CHS or 0.017% DDM and 0.0034% CHS). HRV 3C protease was added to the column and incubated for 1 h before the protein of interest was eluted from the column with wash buffer. The protein was concentrated on an Amicon Ultra centrifugal filter (50-kDa molecular mass cutoff; Merck Millipore). For cryo-EM samples, the appropriate Fab, expressed and purified as described below, was added followed by anti-κ light chain nanobody, expressed and purified as described below, in a 1:2:4 molar ratio of protein, Fab and nanobody, respectively. The complex was allowed to assemble for at least 10 min on ice before the next steps were carried out.

For LMNG–CHS-reconstituted samples, SEC was performed using a Superdex 200 increase 10/300 column (GE Healthcare) equilibrated in SEC buffer without detergent (150 mM sodium chloride and 20 mM HEPES pH 7.5) and run at 0.5 ml min^−1^. For DDM–CHS-reconstituted samples, SEC was performed using a Superdex 200 increase 10/300 column (GE Healthcare) equilibrated in SEC buffer with detergent (150 mM sodium chloride, 20 mM HEPES pH 7.5, 0.017% DDM and 0.0034% CHS) and run at 0.5 ml min^−1^. Fractions containing the protein of interest were concentrated as before and either directly used or flash-frozen in liquid nitrogen and stored at −80 °C. For nanodisc-reconstituted samples, DDM–CHS-reconstituted *Hs*ALG9 in complex with Fab and elbow nanobody was mixed with membrane scaffold protein 1D1 (ref. ^73^) and porcine liver polar lipid extract (Avanti Polar Lipids) mixed 4:1 (w/w) with cholesterol (Avanti Polar Lipids) in a 1:5:120 molar ratio. The lipids were mixed in chloroform, dried, washed in diethyl ether, dried, resuspended in nanodisc buffer (20 mM HEPES pH 7.5 and 150 mM sodium chloride) and stored at −80 °C until needed^74^. Before nanodisc assembly, the lipid suspension was thawed and 1% DDM and 0.2 % CHS were added; the mixture was sonicated for approximately 1 h. The lipids were mixed with protein and incubated for 5 min at room temperature with rotation before adding the membrane scaffold protein and incubating for a further 20 min at room temperature. Activated Bio-Beads SM-2 polystyrene beads (Bio-Rad) were added to 0.8 g (wet weight) of Bio-Beads per ml of sample and incubated with rotation at room temperature for 10 min before being moved to 4 °C overnight^74^. Following nanodisc reconstitution, the sample was further purified using a Superdex 200 increase 10/300 column (GE Healthcare) equilibrated in nanodisc buffer and run at 0.5 ml min^−1^. Fractions containing the protein of interest were concentrated as before and used to prepare cryo-EM grids.

### Chemoenzymatic substrate synthesis

Acceptor substrates were enzymatically extended from chemically synthesized Dol25-PP-GlcNAc_2_ (refs. ^34,35,75^). Dol25-PP-GlcNAc_2_ was extended to Dol25-PP-GlcNAc_2_Man_5_ in a one-pot reaction with *Sc*ALG1, *Hs*ALG2 and *Sc*ALG11 (ref. ^34^). *Sc*ALG3, *Hs*ALG9 and *Gg*ALG12 were then used to further extend the acceptor substrate as desired. All enzymes were extracted and purified in DDM–CHS detergents.

A 1:400 molar ratio of enzyme to acceptor substrate was used for *Sc*ALG3, *Hs*ALG9 and *Gg*ALG12 with an excess of chemically synthesized Dol25-P-Man donor substrate or with submolar amounts of donor substrate regenerated in situ^35,36^. Donor recycling was accomplished using DPMS from *Pyrococcus* *furiosus*, GDP-mannose and 10 mM MgCl_2_ (ref. ^36^). DPMS was used at a 1:100 molar ratio of enzyme to acceptor substrate. Reactions were incubated at room temperature overnight in 150 mM sodium chloride, 20 mM HEPES pH 7.5, 0.017% DDM and 0.0034% CHS. Donor product Dol25-P was removed after synthesis, as needed, by the transfer of GlcNAc-1-phosphate from UDP-GlcNAc to Dol25-P using AglH (UniProt P39465) from *Sulfolobus* *acidocaldarius* at 45 °C overnight^36^. AglH was included at a concentration of ~2–3 µM with excess UDP-GlcNAc^36^. LLOs were purified as needed with solvent extraction of lyophilized product using a 2:1 volumetric ratio of chloroform to methanol, followed by a 10:10:2 volumetric ratio of chloroform to methanol to water^34^.

### Activity assays with *Sc*ALG3, *Hs*ALG9 and *Gg*ALG12

Mutants were prepared using site-directed mutagenesis and enzyme variants were purified as for the WT enzyme. Activity assays were performed with 100 nM WT or enzyme variant, 10 µM acceptor substrate and 20 µM donor substrate. Reactions were carried out at room temperature in reaction buffer (150 mM sodium chloride, 20 mM HEPES pH 7.5, 0.01% LMNG and 0.001% CHS) for 30 s before being heated to 95 °C for 10 min unless otherwise noted. For activity assays with Fabs, the protein was preincubated with a tenfold molar excess of Fab before the addition of substrates and reacted for 60 s before heating. The resulting LLO samples were then transferred to a peptide fluorescently labeled with carboxytetramethylrhodamine (TAMRA) using *Tb*STT3B (ref. ^36^). The peptide had the sequence: TAMRA-YA**N**ATS-NH_2_ (Genscript), with the glycosylated asparagine residue shown in bold. Briefly, 200 nM *Tb*STT3B was incubated with 5 µM LLO and 10 µM peptide in transfer buffer (150 mM sodium chloride, 20 mM HEPES pH 7.5, 10 mM MnCl_2_, 0.035% DDM and 0.007% CHS) at 30 °C for at least 4 h. The glycopeptides were then separated using tricine–SDS–PAGE and imaged with a fluorescence scanner^76^.

Liquid chromatography (LC)–MS/MS glycopeptide analysis was performed at the Functional Genomics Center Zürich. The samples were diluted 20-fold with 0.1% formic acid before injection of 2 µl on a calibrated Fusion Lumos MS instrument (Thermo Fischer Scientific) coupled to a nano-Acquity ultrahigh-performance LC system (Waters). Peptides were loaded onto a nanoEase M/Z Symmetry C18 trap column (180 µm × 20 mm, 100 Å, particle size: 5 μm) and separated on a nanoEase M/Z HSS C18 T3 column (75 μm × 150 mm, 100 Å, particle size: 1.8 μm), at a constant flow rate of 300 nl min^−1^, with a column temperature of 50 °C and a linear gradient of 2% to 35% acetonitrile with 0.1% formic acid in 20 min and then 35% to 98% acetonitrile with 0.1% formic acid in 5 min, before being held isocratically for another 5 min. The MS instrument was operated in data-dependent acquisition mode; one scan cycle comprised a full-scan MS survey spectrum, followed by up to 12 sequential higher-energy collision-induced dissociation (HCD) MS/MS runs on the most intense signals above a threshold of 1 × 10^4^. Full-scan MS spectra (300–1,800 *m*/*z*) were acquired in the FT-Orbitrap at a resolution of 120,000 at 400 *m*/*z*, while HCD MS/MS spectra were recorded in the FT-Orbitrap at a resolution of 60,000 at 400 *m*/*z*. HCD was performed with a target value of 1 × 10^5^ and a normalization collision energy of 32 was applied. Automatic gain control target values were 5 × 10^5^ for full Fourier-transform MS. For all experiments, dynamic exclusion was used with a single repeat count, repeat duration of 15 s and exclusion duration of 30 s.

### Assays to detect donor hydrolysis by *Hs*ALG9-D82A and *Gg*ALG12-E35Q

First, the Dol25-P-Man donor substrate was titrated to determine the minimum concentration needed to completely extend a given concentration of acceptor substrate. This concentration was then used to determine whether donor hydrolysis was occurring. *Hs*ALG9-D82A was incubated at 16 µM with 8 µM Dol25-PP-GlcNAc_2_Man_6_ and 12 µM Dol25-P-Man for 1 h on ice, whereas *Gg*ALG12-E35Q was incubated at 10 µM with 5 µM Dol25-PP-GlcNAc_2_Man_7_ and 10 µM Dol-P-Man for 1 h on ice. By incubating with an excess of enzyme, we reasoned that our system could be used to detect donor hydrolysis even in the absence of substrate turnover. The various substrate combinations and concentrations were also incubated without enzyme. The timeframe of 1 h on ice was chosen to simulate the maximum time the proteins would be mixed with substates before vitrification for cryo-EM. Experiments with *Hs*ALG9-D82A were performed with protein purified in reaction buffer (150 mM sodium chloride and 20 mM HEPES pH 7.5) with DDM–CHS while *Gg*ALG12-E35Q was performed using protein purified with LMNG–CHS. Following incubation on ice, the samples were heated to 95 °C for 10 min and then cooled. The samples with and without enzyme were then divided into three tubes to determine whether donor hydrolysis had occurred. To the first tube, nothing was added. To the second tube, 1 µM *Hs*ALG9 WT was added to samples with Dol25-PP-GlcNAc_2_Man_6_ or 1 µM *Gg*ALG12 WT was added to samples with Dol25-PP-GlcNAc_2_Man_7_. The third tube was treated as the second tube but with the addition of 20 µM Dol-P-Man. These tubes were incubated for 10 min at room temperature for mannose transfer to occur. The resulting LLOs were transferred to a fluorescently labeled peptide (5-FAM-GSDANYTYTQ-NH_2_) using *Tb*STT3A (ref. ^77^). Briefly, 200 nM *Tb*STT3A was incubated with 5 µM LLO and 10 µM peptide in transfer buffer (150 mM sodium chloride, 20 mM HEPES pH 7.5, 10 mM MnCl_2_, 0.035% DDM and 0.007% CHS). The glycopeptides were then separated using tricine–SDS–PAGE and imaged with a fluorescence scanner^76^. If we observed full conversion of acceptor substrate, we interpreted this as indicating that hydrolysis of donor substrate did not occur during the incubation with the inactive enzyme variant.

### Generation of biotinylated *Sc*ALG3, *Hs*ALG9 and *Gg*ALG12 for phage display

*Sc*ALG3, *Hs*ALG9 and *Gg*ALG12 constructs were modified using restriction-free cloning to include the 15-aa AviTag sequence^78^. Proteins were expressed and purified as without the AviTag in LMNG–CHS detergent. Purified protein was biotinylated overnight at 4 °C with *Escherichia* *coli* BirA (protein-to-BirA molar ratio ranging from 1:1 to 5:1) in biotinylation buffer (150 mM sodium chloride, 50 mM bicine pH 8.3, 10 mM magnesium acetate, 10 mM ATP, 250 µM biotin, 0.01% (w/v) LMNG and 0.001% (w/v) CHS). Following biotinylation, SEC was performed on a Superdex 200 increase column (300/10) equilibrated in SEC buffer (150 mM sodium chloride, 20 mM HEPES pH 7.5, 0.01% (w/v) LMNG and 0.001% (w/v) CHS). The level of biotinylation was evaluated by pulldown using Streptavidin MagneSphere paramagnetic particles (Promega). Biotinylated protein was flash-frozen in liquid nitrogen and stored at −80 °C until needed.

### Phage display selection

AviTagged *Sc*ALG3, *Hs*ALG9 and *Gg*ALG12, biotinylated through the AviTag, were used for phage display selection. Before the campaign, the labeling (biotinylation) efficiency of the protein was verified by a pulldown assay on Streptavidin magnetic beads. Phage display selection was performed at 4 °C according to published protocols^39^. The selection buffer was 25 mM HEPES pH 7.5, 150 mM NaCl, 0.02% LMNG, 0.004% CHS and 0.5% BSA. In the first round, 250 nM of target was immobilized on 250 µl of Streptavidin magnetic beads. Then, 100 μl of a phage library E^40^ was added to the target bound to the Streptavidin beads and incubated for 30 min. The resuspended beads containing bound phages were washed extensively and then used to infect log phase *E*. *coli* XL1-Blue cells. Phages were amplified overnight in 2×YT medium with 50 µg ml^−1^ ampicillin and 10^9^ plaque-forming units per ml of M13-KO7 helper phage. To obtain binders of high affinity and specificity, four additional rounds of selection were performed while decreasing the target concentration in each round, with the final concentration of the target being 10 nM in the fifth round of selection for all three target proteins. In each round, the amplified pool of phages from the preceding round was used as the input. From the second round onward, the bound phages were eluted using 100 mM glycine pH 2.7. This elution technique often results in the elution of nonspecific and Streptavidin binders. To eliminate them, the precipitated phage pool from the second round onward was negatively selected against 50 µl of Streptavidin magnetic beads before adding to the target. The precleared phage pool was then used as input for the selection.

### Single-point phage ELISA

The ELISA experiments were performed at 4 °C in 96-well plates coated with 50 µl of 2 µg ml^−1^ neutravidin in Na_2_CO_3_ buffer (pH 9.6) and subsequently blocked by 1.0% BSA in PBS. A single-point phage ELISA was used to rapidly screen the binding of the obtained Fab fragments displayed on phage. Colonies of *E*. *coli* XL1-Blue harboring phagemids from the fifth round of selection were inoculated directly into 500 μl of 2×YT broth supplemented with 100 μg ml^−1^ ampicillin and M13-KO7 helper phage. The cultures were grown overnight at 37 °C in a 96-deep-well block plate. The ELISA buffer was identical to that used in selection. The experimental wells in the ELISA plates were incubated with 25 nM of respective biotinylated target proteins (ALG3, ALG9 and ALG12) in ELISA buffer for 20 min. Only buffer was added to the control wells. Overnight culture supernatants containing Fab phage were diluted tenfold in ELISA buffer. The diluted phage supernatants were then transferred to ELISA plates that were preincubated with biotinylated target and washed with ELISA buffer. The ELISA plates were incubated with the phage for another 20 min and then washed with ELISA buffer. The washed ELISA plates were incubated with a 1:1 mixture of mouse anti-M13 monoclonal antibody (GE, 27-9420-01; 1:5,000 dilution in ELISA buffer) and peroxidase-conjugated goat anti-mouse IgG (Jackson Immunoresearch, 115-035-003; 1:5,000 dilution in ELISA buffer) for 30 min. The plates were washed again, developed with TMB substrate and then quenched with 1.0 M HCl; the absorbance at 450 nm was determined. The background binding of the phage was monitored by the absorbance from the control wells. The sequences of the clones exhibiting high binding over background were verified by DNA sequencing.

### Expression and purification of Fabs

Fabs were expressed in *E*. *coli* (BL12 (DE3) or C43) in Luria–Bertani or ZYP-5052 autoinduction medium at 30 °C overnight^79^. Fabs were purified using a 5-ml protein-G column and dialyzed into buffer (150 mM sodium chloride and 20 mM HEPES pH 7.5)^79^.

### Multipoint protein ELISA for determination of half-maximal effective concentration (EC_50_)

Multipoint ELISA was performed at 4 °C to estimate the affinity of the Fabs to ALG3, ALG9 and ALG12. The ELISA buffer consisted of 25 mM HEPES pH 7.4, 150 mM NaCl, 0.02% LMNG and 0.004% CHS supplemented with 0.5% BSA. First, 25 nM of target immobilized on a neutravidin-coated ELISA plate was incubated with threefold serial dilutions of the purified Fabs starting from 4 μM for 20 min. The plates were washed and the bound target-Fab complexes were incubated with a secondary horseradish-peroxidase-conjugated Pierce recombinant protein L (Thermo Fisher, 32420; 1:5,000 dilution in ELISA buffer) for 30 min. The plates were again washed, developed with TMB substrate and quenched with 1.0 M HCl; the absorbance at 450 nm was determined. To determine the affinities, the data were fitted to a dose–response sigmoidal function in GraphPad Prism and EC_50_ values were calculated.

### Expression and purification of anti-κ light chain nanobody

The anti-κ light chain nanobody^42^ was expressed using *E*. *coli* BL21 (DE3) cells with ZYP-5052 autoinduction medium^80^ and was purified by nickel affinity chromatography^81^.

### Cryo-EM

For preparation of cryo-EM grids, detergent-reconstituted *Sc*ALG3 and GgALG12 were concentrated to 21 or 33 µM, respectively. For the *Sc*ALG3 sample, 50 µM Dol25-PP-GlcNAc_2_Man_5_ and 100 µM Dol25-P-Man were added before vitrification. For *Gg*ALG12, 150 µM Dol25-PP-GlcNAc_2_Man_7_ and 300 µM Dol25-P-Man were included before plunging the grids. For the detergent-reconstituted samples, 100 µM fluorinated octyl-maltoside (Anatrace) was added directly before plunging. *Hs*ALG9, reconstituted in nanodiscs, was concentrated to 12 µM before the addition of 50 µM Dol25-PP-GlcNAc_2_Man_6_ or Dol25-PP-GlcNAc_2_Man_8_ acceptor substrate and 100 µM Dol25-P-Man donor substrate. For all datasets collected, Quantifoil holey carbon film (R1.2/1.3) 300-mesh grids with gold support were glow-discharged before the addition of 3 µl of sample. Vitrification was performed using a Vitrobot Mark IV (Thermo Fisher) set to 4 °C and 100% relative humidity. Grids were blotted for 2.5–4.5 s before plunging into liquid propane and ethane.

Datasets were collected at the ScopeM facility at ETH Zürich using a 300-kV Titan Krios (Thermo Fischer Scientific) transmission EM instrument equipped with a K3 Summit direct electron detector (Gatan) and a GIF Quantum energy filter (Gatan) operated at a slit width of 12 or 20 keV. Fully automated collection of movies was performed in EPU2 (Thermo Fischer Scientific) with a defocus range set from −0.6 to −2.8 µm or from −0.6 to −2.2 µm. The *Hs*ALG9 second ternary complex and the ALG12 datasets were collected in correlated double-sampling mode. Data collection parameters for each dataset are listed in Extended Data table 1.

### Cryo-EM data processing

Cryo-EM data were processed using cryoSPARC version 4 (ref. ^82^) as shown in Extended Data Fig. 3. Similar processing strategies were used for each dataset. Beam-inducted motion correction and contrast transfer function (CTF) estimation were performed using patch motion correction and patch CTF. Micrographs with a CTF fit resolution worse than 4.5 Å for the *Sc*ALG3 and *Hs*ALG9 datasets or 3.5 Å for the *Gg*ALG12 dataset were discarded. Particles were initially picked using the blob picker on a subset of micrographs before two-dimensional (2D) classification and ab inito reconstruction was used to create an initial model for template-based particle picking of the full dataset. Particles were initially extracted with a box size of 432 or 512 pixels and 2× or 4× binning. Iterative heterogeneous refinement with two or three classes was used with or without 2D classification to select the best particles with bound Fab. The best particles were reextracted without binning and taken forward for nonuniform refinement. Three-dimensional classification, reference-based per-particle motion correction, per-particle and per-group CTF optimization, masking and local refinement were used as needed to improve resolution. Refined maps were either sharpened in cryoSPARC or using DeepEMhancer^83^.

### Model building and refinement

Models were initially generated de novo using models for the ALG enzymes predicted by ModelAngelo^84^ or AlphaFold2 (ref. ^85^), while an anti-κ light chain nanobody–Fab complex model (PDB 6WW2)^86^ was used for the Fab and nanobody regions of the complex. The models were rigid-body fitted into the map using ChimeraX^87^ and manually build as required in Coot^88^. Models were initially refined using Rosetta-based refinement and local density-guided optimization implemented in StarMap^89–91^. Ligand restraints were prepared using AceDRG^92^ and models were iteratively refined in the PHENIX software suite^93^ using phenix.real_space_refine^94^, followed by manual manipulation in Coot. Models were evaluated using MolProbity^95^ and validation tools implemented in PHENIX^96^. Figures were prepared using ChimeraX and Adobe Illustrator. Sequences were aligned using Clustal Omega^97^ within the EMBL-EBI Job Dispatcher^98^ and prepared for publication using ESPript 3.0 (ref. ^99^) and Adobe Illustrator.

### MD simulation and analysis

The initial model for the *Hs*ALG9 MD simulations with donor substrate (Dol25-P-Man) and the second acceptor substrate (Dol25-PP-GlcNAc_2_Man_8_) was taken from the EM-refined model. The complex was embedded in a lipid bilayer comprising 400 phosphatidylcholine (POPC) molecules and solvated in a water box (distance from the top of *Hs*ALG9 to the wall = 22.5 Å) with 0.15 M sodium chloride using CHARMM-GUI^100^ and the PPM server^101^, leading to a box size of 125.86 Å × 126.20 Å × 103.07 Å. The D82A substitution was restored to WT and the disulfide bond between C414 and C480 was built using tleap from AmberTools22 (ref. ^102^). The atomic structure of the unresolved mannose was manually added onto the donor product with the donor anomeric carbon facing toward the oxygen of the acceptor C2 hydroxyl in VMD^103^. The energy of the system was first minimized by 70,000 steps with positional restraints on the solute atoms with a force constant of 0.418 kJ mol^−1^ nm^−2^. The same minimized system was equilibrated five times with different random number seeds for the generation of the initial velocities consisting of a 75-ps NVT simulation and a 650-ps NPT simulation at 303.15 K using Langevin dynamics^104^ with a friction coefficient of 1 ps^−1^ and a gradually decreasing force constant for the positional restraints on the solute atoms (from 0.418 kJ mol^−1^ nm^−2^ to 0.004 kJ mol^−1^ nm^−2^). An 800-ns-long NPT production run was performed starting from each equilibrated system. The printout frequency for analysis was 0.2 ns. For the NPT simulations, an anisotropic Monte Carlo barostat^105,106^ was used to keep the pressure at 1 bar with a coupling constant of 0.5 ps and a volume change attempted every 0.2 ps. Nonbonded interactions were treated with a cutoff distance of 9 Å and long-range electrostatic interactions were taken into account with the particle mesh Ewald method^107,108^. The simulations were integrated with a time step of 2 fs and SHAKE^109^ bond-length constraints on hydrogen with a geometrical tolerance of 10^−6^. The translational and rotational center-of-mass motion was removed every 2 ps. Atomic interactions were described by the classical force fields AMBER ff19SB (ref. ^110^) for the proteins, lipid21 (ref. ^111^) for POPC, GAFF2 (ref. ^112^) for donor and acceptor substrates and OPC^113^ for water molecules, with Joung/Cheatham^114^ ion parameters for ions. Partial charges of the donor and acceptor substrates were assigned with the AM1-BCC^115^ method using antechamber^116^. Energy minimization and MD simulations were conducted with the pmemd.MPI and pmemd.cuda engines in Amber22 (University of California, San Francisco), respectively.

Among the five simulations from the initial guess of the donor mannose position, one of them exhibited a stable geometry at the catalytic center. The final frame from this simulation was taken as the initial structure for the subsequent five systems, including Dol25-P-Man, Dol25-P-Glc and Dol25-P-Man-2F with WT *Hs*ALG9 and Dol25-P-Man with *Hs*ALG9 mutants N288A, H366A and D82A. Five repeats of 800-ns-long simulations were performed following the same procedure as described above. The changes from Dol25-P-Man to Dol25-P-Glc and Dol25-P-2F were performed with PyMol Builder (Schrödinger) and the N288A and H366A substitutions were realized using tleap from AmberTools22 (ref. ^102^). For MD simulation analysis, hydrogen bonds were defined as being present with default settings in pytraj (acceptor–hydrogen–receptor angle cutoff of 135° and a donor–acceptor cutoff of 3 Å)^117^.

### Reporting summary

Further information on research design is available in the Nature Portfolio Reporting Summary linked to this article.

## Online content

Any methods, additional references, Nature Portfolio reporting summaries, source data, extended data, supplementary information, acknowledgements, peer review information; details of author contributions and competing interests; and statements of data and code availability are available at 10.1038/s41589-026-02164-7.

## Supplementary information

Supplementary Information Supplementary Figs. 1–7.

Reporting Summary

Peer Review File

## Source data

Source Data Fig. 1 Unprocessed gels.

Source Data Fig. 4 Unprocessed gels.

Source Data Fig. 5 Statistical source data.

Source Data Extended Data Fig. 1 Unprocessed gels.

Source Data Extended Data Fig. 2 Unprocessed gels.

Source Data Extended Data Fig. 2 Statistical source data.

Source Data Extended Data Fig. 8 Unprocessed gels.

## Figures and Tables

**Extended Data Table 1:**
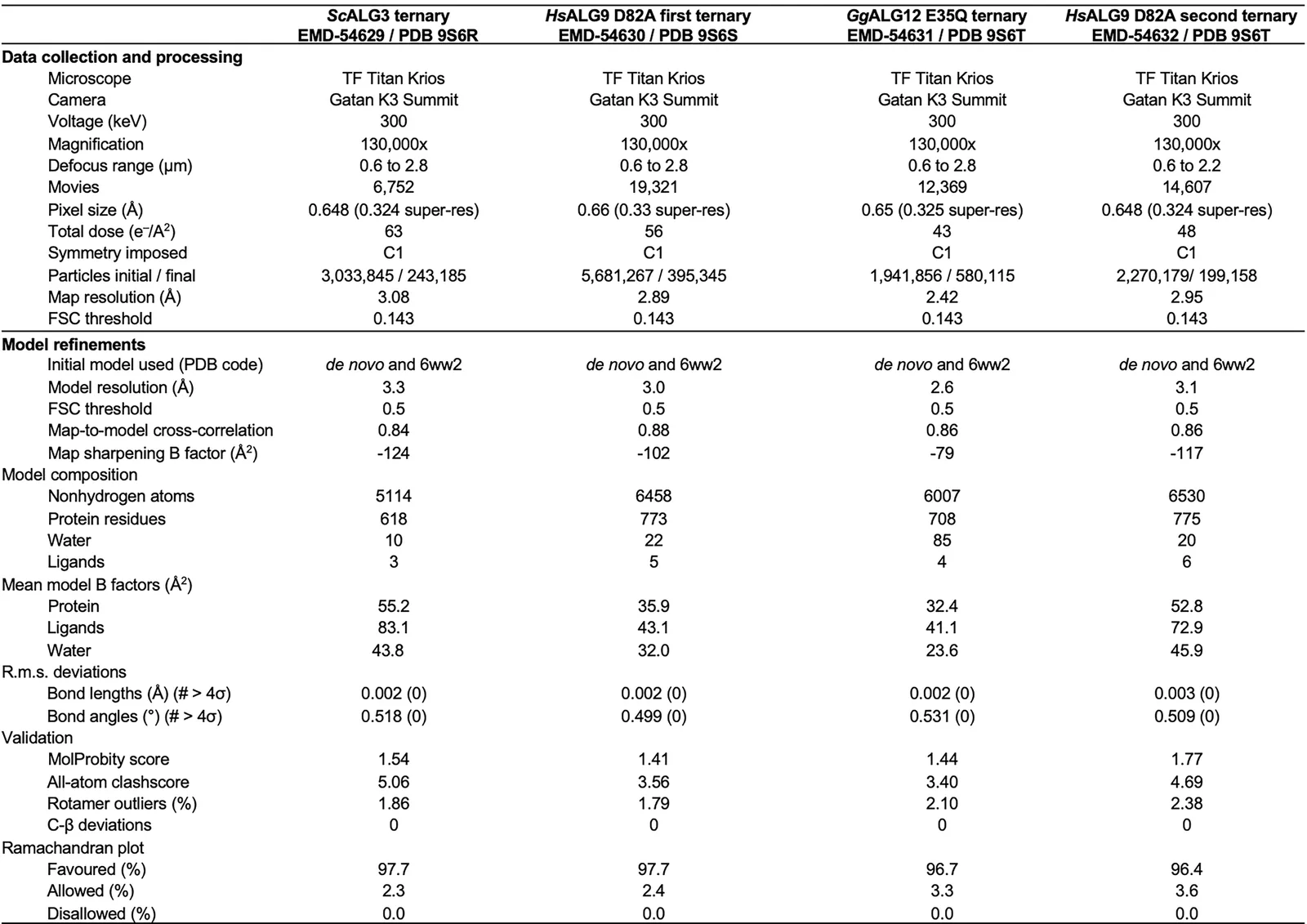
Cryo-EM data collection, processing and model statistics

**Extended Data Table 2:**
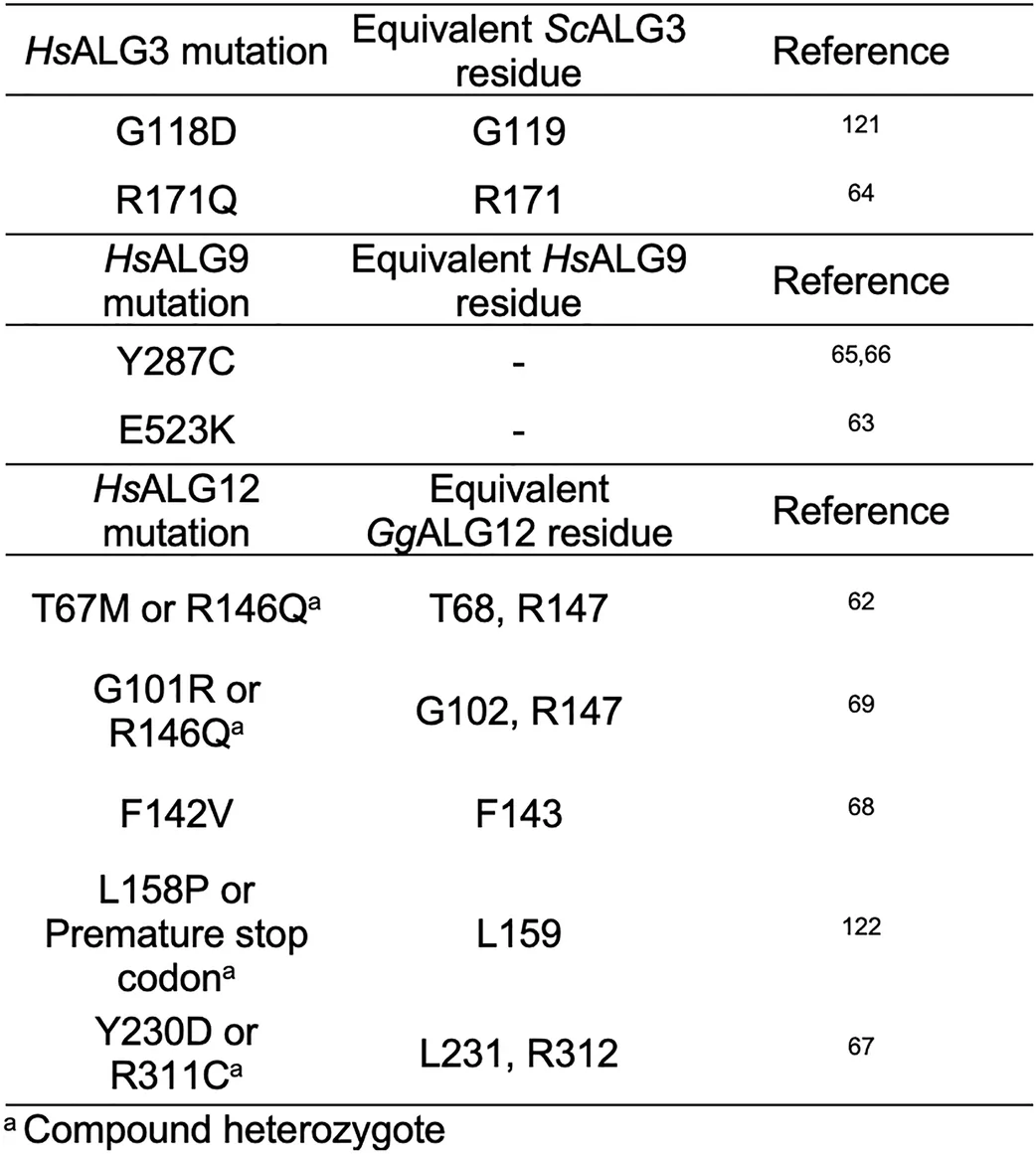
Clinically observed mutations in human ALG3, ALG9 and ALG12 causing congenital disorders of glycosylation

For additional details, see refs. ^62–69, 121, 122^.

## Data Availability

Models and EM maps for the *Sc*ALG3 ternary complex, the first ternary complex of *Hs*ALG9, the *Gg*ALG12 ternary complex and the second ternary complex of *Hs*ALG9 were deposited to the Protein Data Bank with the accession codes 9S6R, 9S6S, 9S6T and 9S6U, respectively, and the EM Data Bank with accession codes EMD-54629, EMD-54630, EMD-54631 and EMD-54632, respectively. The MS glycoproteomics data generated in this study were deposited to the ProteomeXchange Consortium through the PRIDE^118^ partner repository with the dataset identifier PXD072144. Source data are provided with this paper.

## Code availability

The MD topology files, input files, simulation scripts and analysis scripts are freely available from GitHub (https://github.com/rinikerlab/ALG_mannosyltransferase). Because of the large file size (>20 GB without solvent), the tMD trajectories used in this work can be provided upon requests to sriniker@ethz.ch. The solvent-stripped MD trajectories used in this work are provided on Zenodo (10.5281/zenodo.17973165)^119^.
