# Supplementary material for: Structures of ALG3/9/12 reveal the assembly logic of the N-glycan oligomannose core
Source: Nat Chem Biol. 2026 Mar 10;22(9):1501–13. doi: 10.1038/s41589-026-02164-7 (PMC13503287; doi:10.1038/s41589-026-02164-7)
Supplement: Supplementary file 2 [file 41589_2026_2164_MOESM1_ESM.pdf]

nature chemical biology

Supplementary information

https://doi.org/10.1038/s41589-026-02164-7

Structures of ALG3/9/12 reveal the assembly logic of the *N*-glycan oligomannose core

In the format provided by the authors and unedited

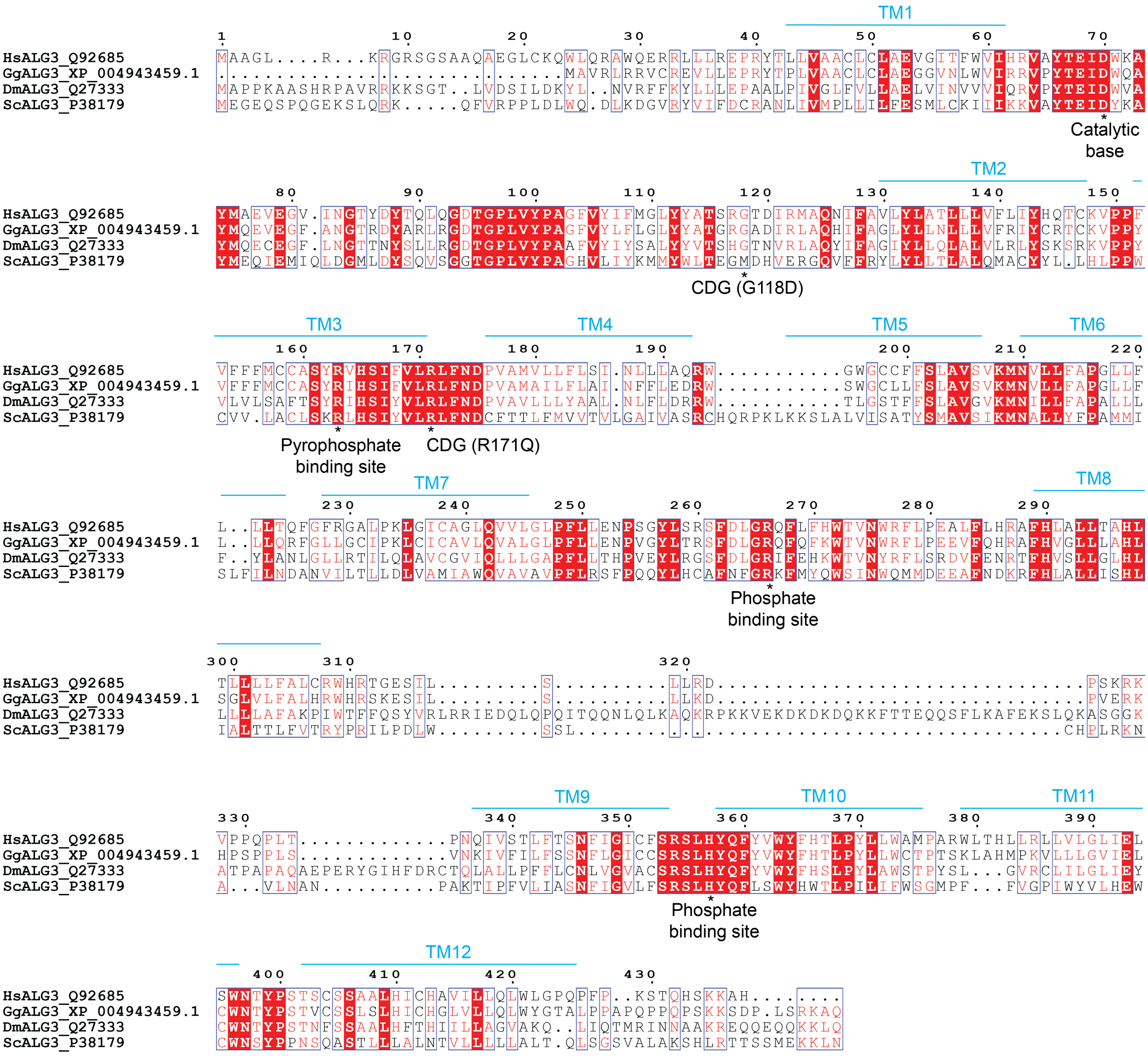

Supplementary Figure 1 |Homologue protein sequence alignments of ALG3 from *Homo sapiens*, *Gallus gallus*, *Drosophila melanogaster* and *Saccharomyces cerevisiae*. The catalytic base, substrate pyrophosphate and phosphate binding residues are indicated along with residues reported to be involved in CDGs. See Extended Data Table 2 for references for the variants associated with CDGs. The protein names are followed by the accession number.

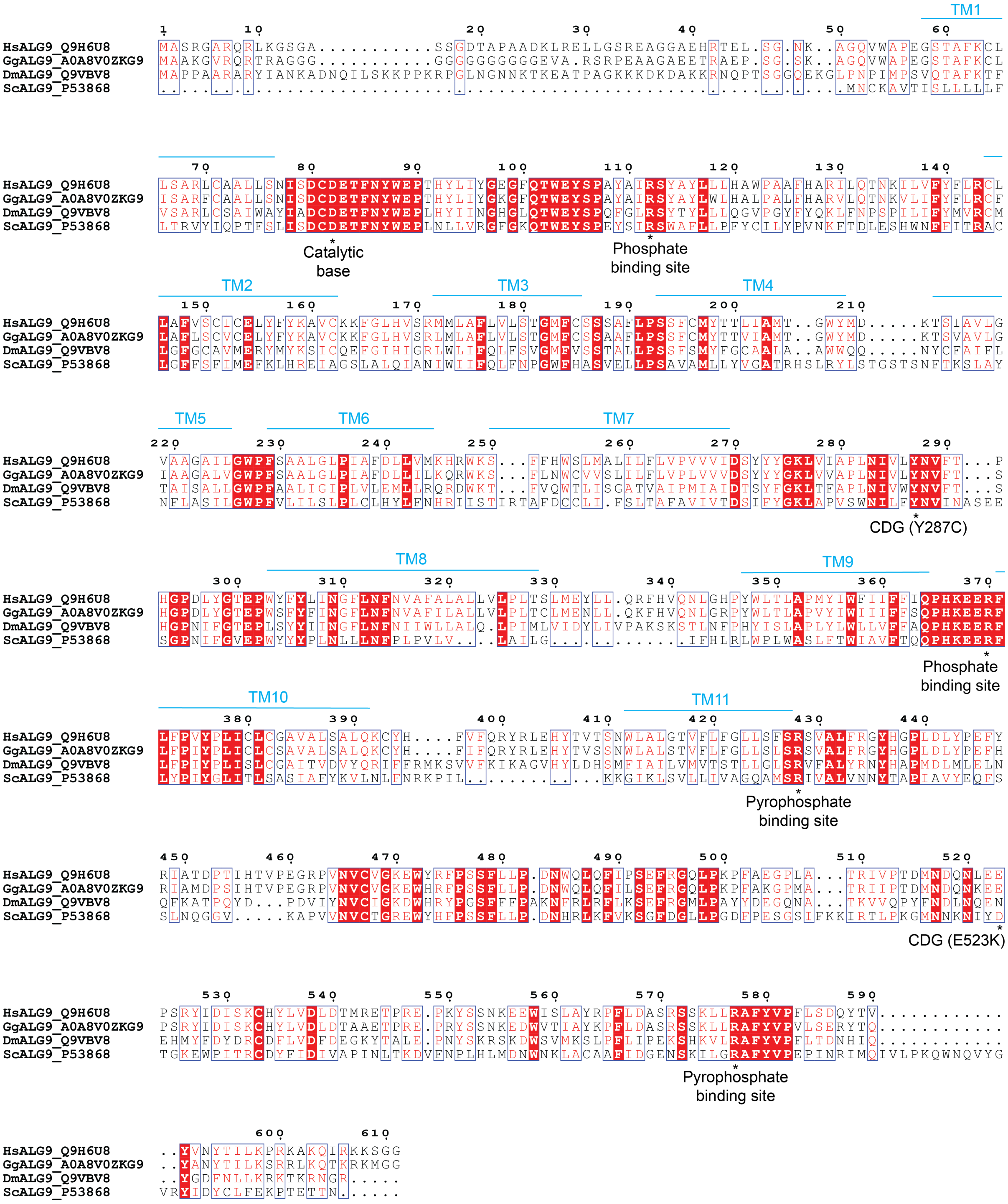

Supplementary Figure 2 |Homologue protein sequence alignments of ALG9 from *Homo sapiens*, *Gallus gallus*, *Drosophila melanogaster* and *Saccharomyces cerevisiae*. The catalytic base, substrate pyrophosphate and phosphate binding residues are indicated along with residues reported to be involved in CDGs. See Extended Data Table 2 for references for the variants associated with CDGs. The protein names are followed by the accession number.

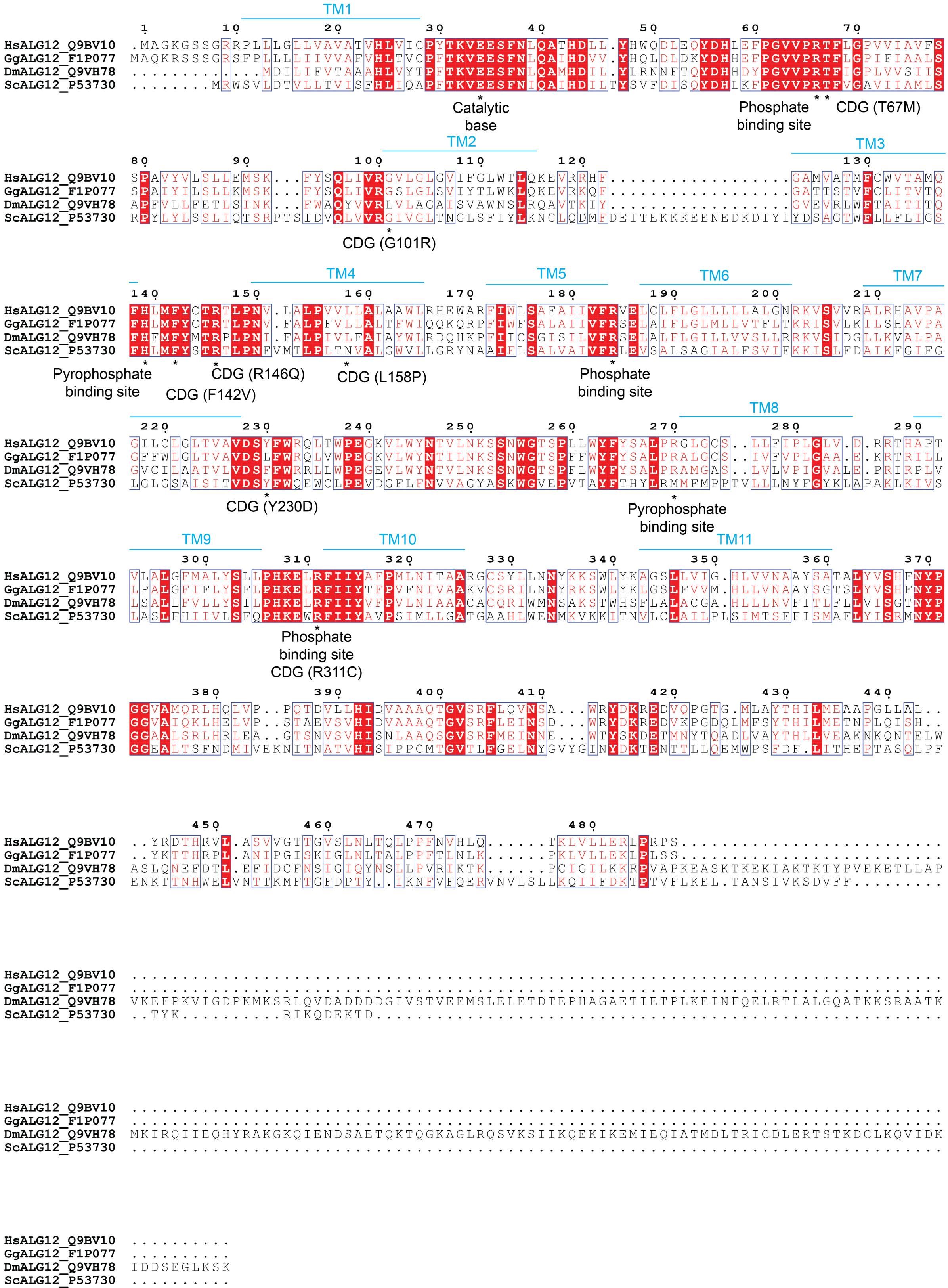

Supplementary Figure 3 |Homologue protein sequence alignments of ALG12 from *Homo sapiens*, *Gallus gallus*, *Drosophila melanogaster* and *Saccharomyces cerevisiae*. The catalytic base, substrate pyrophosphate and phosphate binding residues are indicated along with residues reported to be involved in CDGs. See Extended Data Table 2 for references for the variants associated with CDGs. The protein names are followed by the accession number.

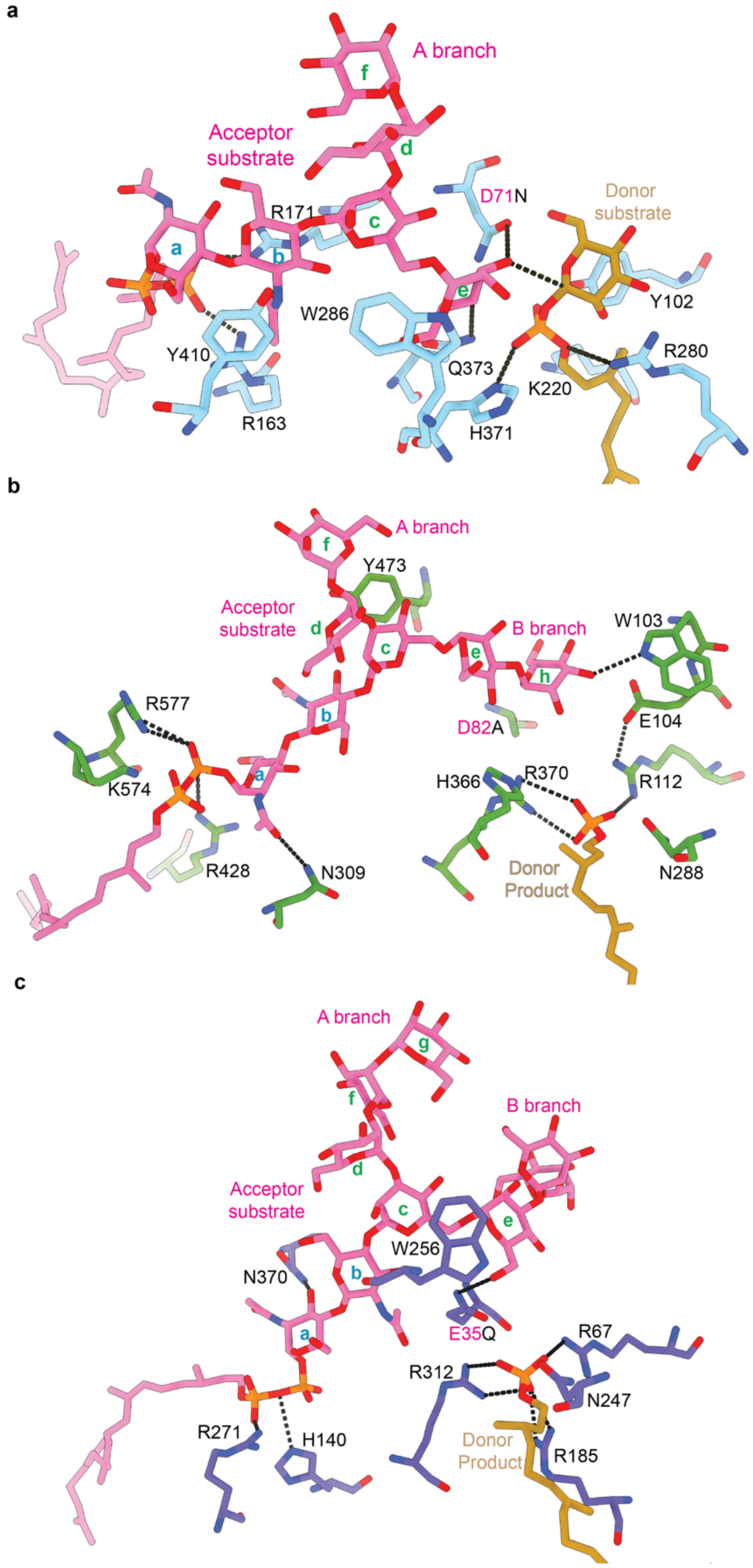

Supplementary Figure 4 | Close up of substrates and selected active site residues. a, *Sc*ALG3, **b,** *Hs*ALG9 and **c.** *Gg*ALG12 ternary complexes. Acceptor and donor substrates are depicted in pink and gold stick representation. Key residues are highlighted as sticks, and hydrogen bonding interactions are shown as dashed lines.

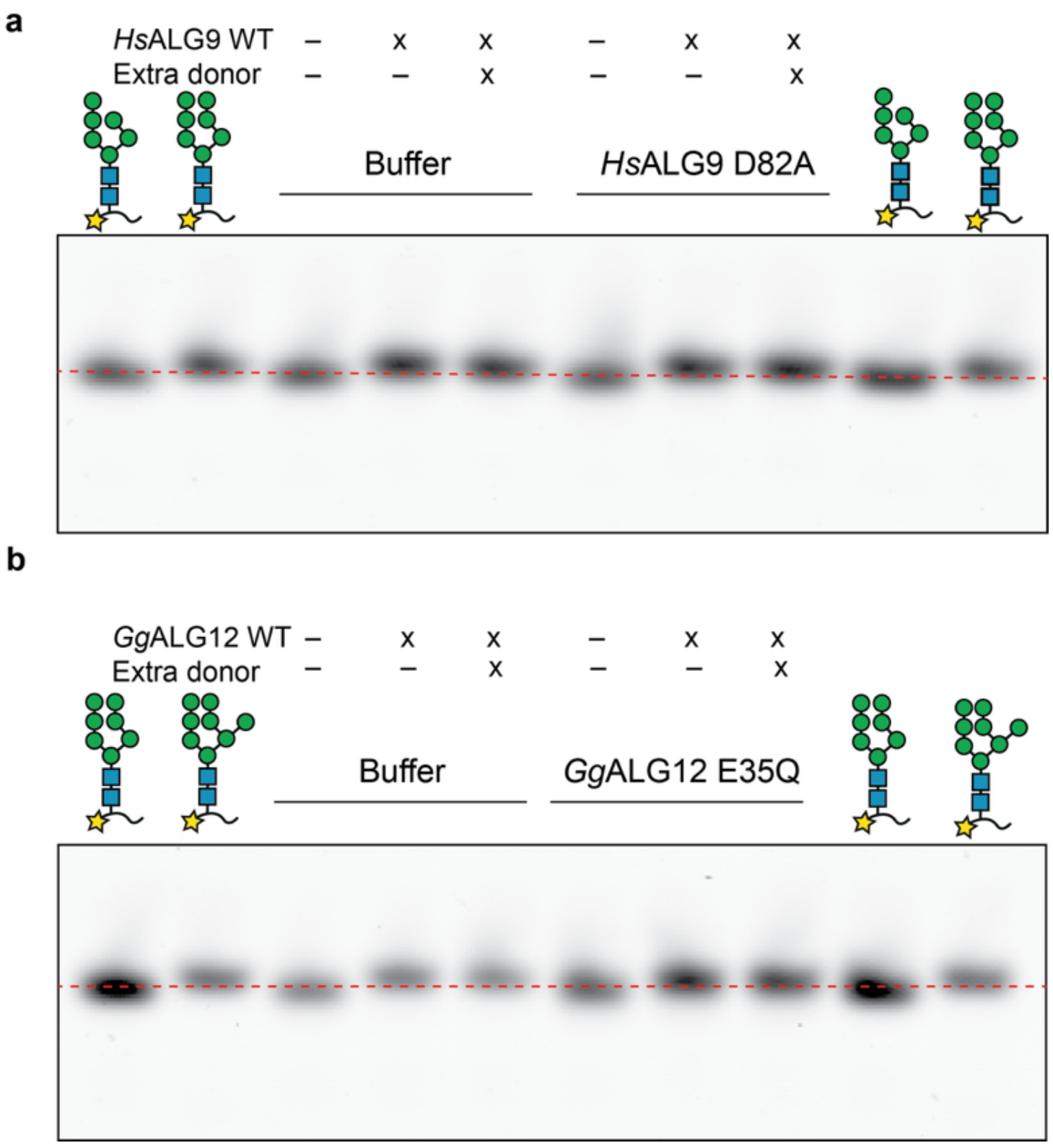

Supplementary Figure 5 | Assay to measure futile donor hydrolysis by *Hs*ALG9 D82A or *Gg*ALG12 E35Q. a, Assay with *Hs*ALG9 D82A. **b,** Assay with *Gg*ALG12 E35Q. After incubation of acceptor and donor substrates for 1 hour on ice as indicated above the gel, the samples were split into three and further processed with either buffer alone, WT enzyme or WT enzyme and fresh donor substrate as indicated above the gel. The reaction products were transferred to a fluorescent peptide (5-carboxyfluorescein-GSDANYTYTQ-NH_2) using *Tb*STT3A and separated by tricine gel electrophoresis. Lanes with glycopeptide cartoons over them serve as glycopeptide standards for the indicated glycan. Assays to measure donor hydrolysis were performed at least twice with similar results.

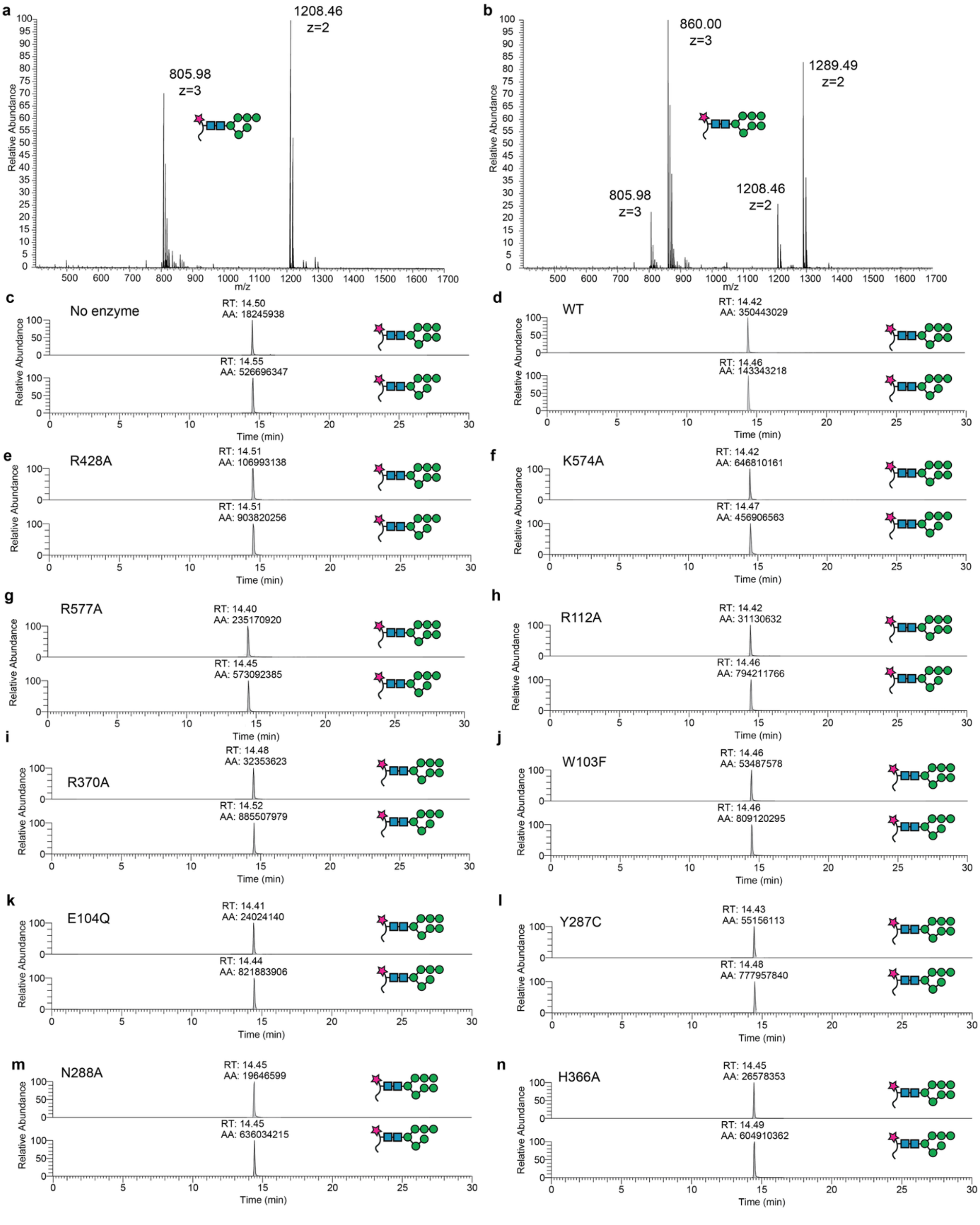

Supplementary Figure 6 | HRMS analysis of glycopeptides from the *Hs*ALG9 activity assay. a, Representative LC-MS analysis of the $GlcNac_2Man_6$ glycopeptide. HRMS (m/z): $[M+2H]^{2+}$ calcd. for $C_{103}H_{148}N_{12}O_{54}^{2+}$, 1208.46; found, 1208.46. $[M+3H]^{3+}$ calcd. for $C_{103}H_{149}N_{12}O_{54}^{3+}$, 805.98; found, 805.98. **b,** Representative LC-MS analysis of the $GlcNac_2Man_7$ glycopeptide. HRMS (m/z): $[M+2H]^{2+}$ calcd. for $C_{109}H_{158}N_{12}O_{59}^{2+}$, 1289.49; found, 1289.49. $[M+3H]^{3+}$ calcd. for $C_{109}H_{159}N_{12}O_{59}^{3+}$, 859.99; found, 860.00. **c-n,** Extracted ion chromatograms for $GlcNac_2Man_6$ and $GlcNac_2Man_7$ glycopeptides from reactions with **c,** no enzyme, **d,** WT, **e,** R428A, **f,** K574A, **g,** R577A **h,** R112A, **i,** R370A **j,** W103A, **k,** E104Q, **l,** Y287C, **m,** N288A and **n,** H366A samples. The analyte areas for the $GlcNac_2Man_6$ (acceptor) and $GlcNac_2Man_7$ (product) glycopeptide are indicated on the spectra.

Supplementary Fig. 5a

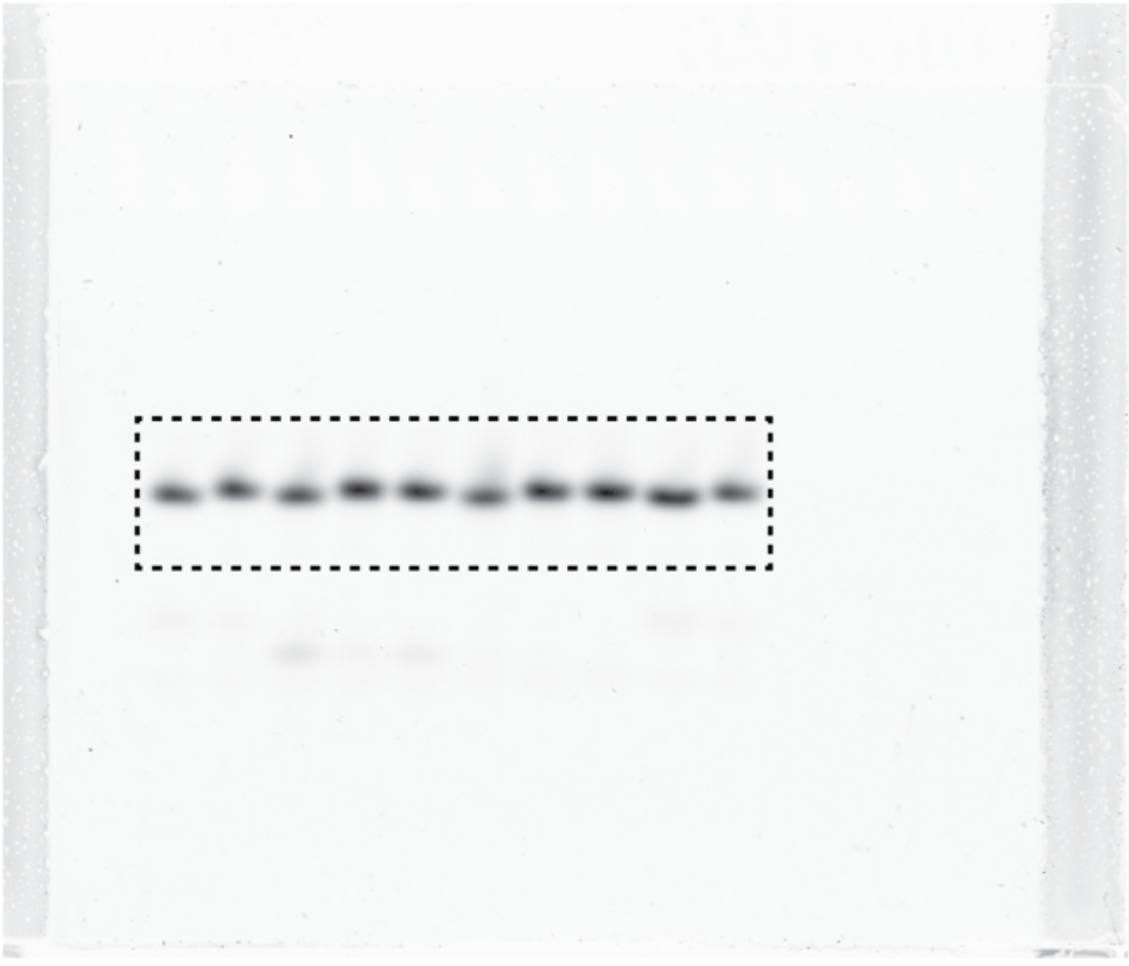

Supplementary Fig. 5b

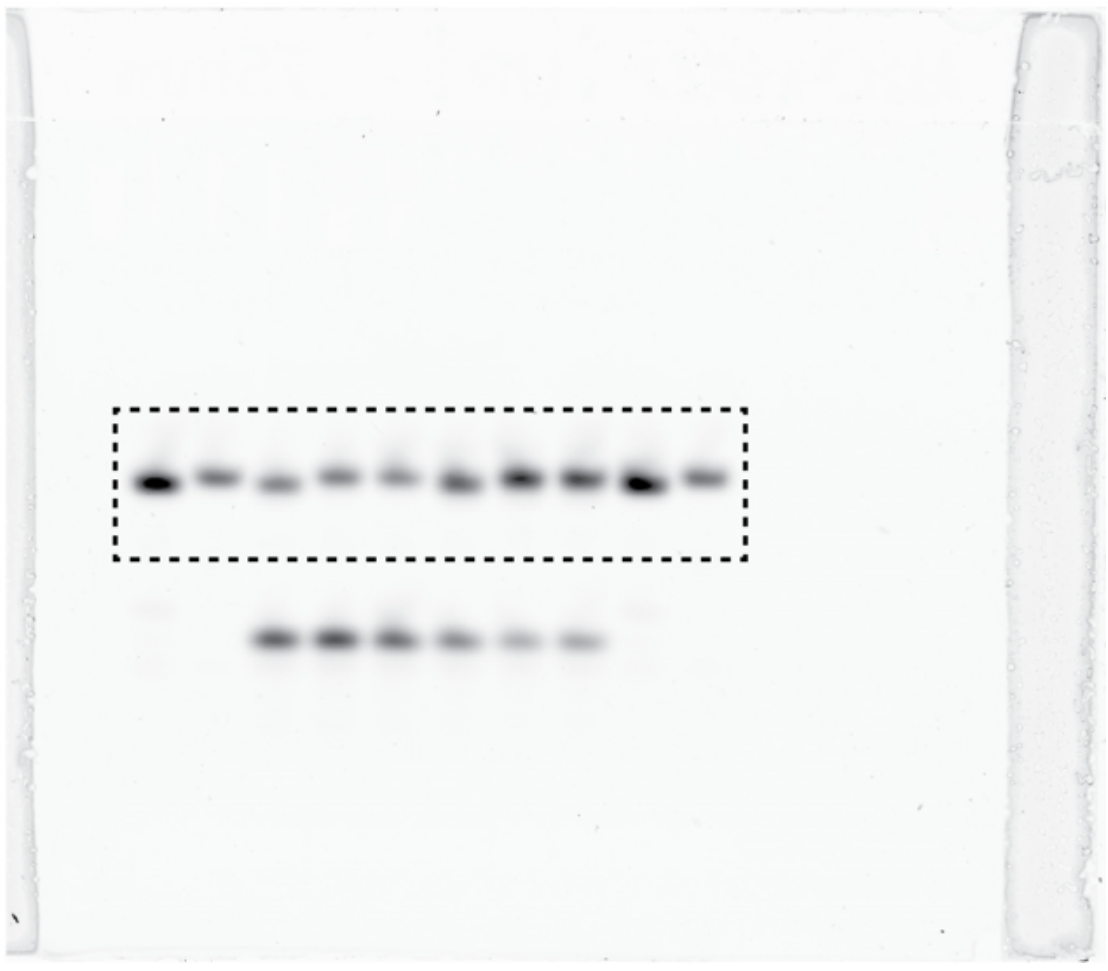

Supplementary Figure 7 | Unprocessed gels for Supplementary Figure 5.
