# Supplementary material for: Structures of ALG3/9/12 reveal the assembly logic of the N-glycan oligomannose core
Source: Nat Chem Biol. 2026 Mar 10;22(9):1501–13. doi: 10.1038/s41589-026-02164-7 (PMC13503287; doi:10.1038/s41589-026-02164-7)
Supplement: Supplementary file 3 [file 41589_2026_2164_MOESM2_ESM.pdf]

nature portfolio

Corresponding author(s): Kaspar P. Locher

Last updated by author(s): Dec 21, 2025

Reporting Summary

Nature Portfolio wishes to improve the reproducibility of the work that we publish. This form provides structure for consistency and transparency in reporting. For further information on Nature Portfolio policies, see our Editorial Policies and the Editorial Policy Checklist.

Statistics

For all statistical analyses, confirm that the following items are present in the figure legend, table legend, main text, or Methods section.

n/a	Confirmed	
☐	☒	The exact sample size (*n*) for each experimental group/condition, given as a discrete number and unit of measurement
☐	☒	A statement on whether measurements were taken from distinct samples or whether the same sample was measured repeatedly
☒	☐	The statistical test(s) used AND whether they are one- or two-sided<br>*Only common tests should be described solely by name; describe more complex techniques in the Methods section.*
☒	☐	A description of all covariates tested
☒	☐	A description of any assumptions or corrections, such as tests of normality and adjustment for multiple comparisons
☐	☒	A full description of the statistical parameters including central tendency (e.g. means) or other basic estimates (e.g. regression coefficient) AND variation (e.g. standard deviation) or associated estimates of uncertainty (e.g. confidence intervals)
☒	☐	For null hypothesis testing, the test statistic (e.g. *F*, *t*, *r*) with confidence intervals, effect sizes, degrees of freedom and *P* value noted<br>*Give P values as exact values whenever suitable.*
☒	☐	For Bayesian analysis, information on the choice of priors and Markov chain Monte Carlo settings
☒	☐	For hierarchical and complex designs, identification of the appropriate level for tests and full reporting of outcomes
☒	☐	Estimates of effect sizes (e.g. Cohen's *d*, Pearson's *r*), indicating how they were calculated

Our web collection on statistics for biologists contains articles on many of the points above.

Software and code

Policy information about availability of computer code

Data collection	Cryo-EM data were collected using EPU2 software (Thermo Fisher Scientific). Gels were imaged using a Azure Sapphire scanner
Data analysis	Data analysis and visualization:<br>UCSF ChimeraX 1.8-1.10<br>GraphPad Prism 10<br>cryoSPARC v4<br>Phenix 1.21.2<br>Coot 0.9.8.96<br>amber24<br>AmberTools24<br>VMD 1.9.4a57

For manuscripts utilizing custom algorithms or software that are central to the research but not yet described in published literature, software must be made available to editors and reviewers. We strongly encourage code deposition in a community repository (e.g. GitHub). See the Nature Portfolio guidelines for submitting code & software for further information.

Data

Policy information about availability of data

All manuscripts must include a data availability statement. This statement should provide the following information, where applicable:

- Accession codes, unique identifiers, or web links for publicly available datasets
- A description of any restrictions on data availability
- For clinical datasets or third party data, please ensure that the statement adheres to our policy

Models and EM maps for the ScALG3 ternary complex, the first ternary complex of HsALG9, the GgALG12 ternary complex and the second ternary complex of HsALG9 have been deposited to the Protein Data Bank and the Electron Microscopy Data Bank with the accession codes 9S6R, 9S6S, 9S6T, 9S6U and EMD-54629, EMD-54630, EMD-54631, EMD-54632, respectively. The mass spectrometry glyco-proteomics data generated in this study have been deposited to the ProteomeXchange Consortium via the PRIDE120 partner repository with the dataset identifier PXD072144 and 10.6019/PXD072144. The MD topology files, input files, simulation scripts, and the analysis scripts are freely available at https://github.com/rinikerlab/ALG_mannosyltransferase. Due to the large file size (> 20GB without solvents), the tMD trajectories used in this work are provided upon requests to sriniker@ethz.ch. The solvent-stripped MD trajectories used in this work are provided at https://doi.org/10.5281/zenodo.17973165

Research involving human participants, their data, or biological material

Policy information about studies with human participants or human data. See also policy information about sex, gender (identity/presentation), and sexual orientation and race, ethnicity and racism.

Reporting on sex and gender	Not applicable
Reporting on race, ethnicity, or other socially relevant groupings	Not applicable
Population characteristics	Not applicable
Recruitment	Not applicable
Ethics oversight	Not applicable

Note that full information on the approval of the study protocol must also be provided in the manuscript.

Field-specific reporting

Please select the one below that is the best fit for your research. If you are not sure, read the appropriate sections before making your selection.

☒ Life sciences ☐ Behavioural & social sciences ☐ Ecological, evolutionary & environmental sciences

For a reference copy of the document with all sections, see nature.com/documents/nr-reporting-summary-flat.pdf

Life sciences study design

All studies must disclose on these points even when the disclosure is negative.

Sample size	6,752 movies were collected for the ScALG3-ternary complex dataset; 19,321 movies were collected for the first HsALG9-ternary complex; 14,607 movies were collected for the second HsALG9-ternary complex dataset; 12,369 movies were collected for the GgALG12-ternary complex dataset
Data exclusions	During cryo-EM data processing particles not contributed to classes of interest were not included in final reconstructions as is standard in cryo-EM data processing strategies.
Replication	Three biological replicates were performed for ELISA experiments. Replication values for other experiments are reported in the figure legends.
Randomization	Not applicable. Samples of known composition were required and used.
Blinding	Not applicable. Samples of known composition were required and used.

Reporting for specific materials, systems and methods

We require information from authors about some types of materials, experimental systems and methods used in many studies. Here, indicate whether each material, system or method listed is relevant to your study. If you are not sure if a list item applies to your research, read the appropriate section before selecting a response.

Materials & experimental systems

n/a	Involved in the study
☐	☒ Antibodies
☐	☒ Eukaryotic cell lines
☒	☐ Palaeontology and archaeology
☒	☐ Animals and other organisms
☒	☐ Clinical data
☒	☐ Dual use research of concern
☒	☐ Plants

Methods

n/a	Involved in the study
☒	☐ ChIP-seq
☒	☐ Flow cytometry
☒	☐ MRI-based neuroimaging

Antibodies

Antibodies used: Sc3-16, Hs9-8 and Gg12-11 Fabs were produced in-house from a synthetic library (as described in methods)

Validation: Biophysical and biochemical characterisation of the antibodies is described in the results and methods sections

Eukaryotic cell lines

Policy information about cell lines and Sex and Gender in Research

Cell line source(s): Human Embryonic Kidney (HEK) suspension 293 c18 (ATCC CRL-10852); Spodoptera frugiperda Sf9 (ATCC CRL-1711)

Authentication: No authentication was performed

Mycoplasma contamination: No contamination tests were performed

Commonly misidentified lines (See ICLAC register): Not applicable

Plants

Seed stocks: Not applicable

Novel plant genotypes: Not applicable

Authentication: Not applicable
