# Supplementary material for: Structures of ALG3/9/12 reveal the assembly logic of the N-glycan oligomannose core
Source: Nat Chem Biol. 2026 Mar 10;22(9):1501–13. doi: 10.1038/s41589-026-02164-7 (PMC13503287; doi:10.1038/s41589-026-02164-7)
Supplement: Supplementary file 4 [file 41589_2026_2164_MOESM3_ESM.pdf]

nature portfolio

Peer Review File

Structures of ALG3/9/12 reveal the assembly logic of the N-glycan oligomannose core

Corresponding Author: Dr Kaspar Locher

Parts of this Peer Review File have been redacted as indicated to maintain the confidentiality of unpublished data.

This file contains all reviewer reports in order by version, followed by all author rebuttals in order by version.

Version 0:

Reviewer comments:

Referee #1

(Remarks to the Author)
In this work, Alexander and co-workers report structures of ALG3, ALG9 and ALG12 bound to chemo-enzymatically generated substrate analogues. Specifically, the authors present cryo-EM structures of (i) ALG3 from Saccharomyces cerevisiae (ScALG3) in complex with Dol25-PP-GlcNAc2Man5 and Dol25-P-Man at 3.3 A, (ii) ALG9-D82A from Homo sapiens (HsALG9) in complex with Dol25-PP-GlcNAc2Man6 and Dol25-PP-GlcNAc2Man8, both in complex Dol25-P-Man at 3.0 A and 3.1 A, respectively, and ALG12-E35Q from Gallus gallus (GgALG12) in complex with Dol25-PP-GlcNAc2Man7 and Dol25-P-Man at 2.6 A. In combination with thorough mutagenesis studies and molecular dynamics simulations, these structures reveal the mechanism of GlcNAc2Man5 to GlcNAc2Man9 biosynthesis in four sequential steps. I really enjoyed reading this manuscript. It is clearly and well written and the interpretation of the experimental data support the proposed model for assembly of the N-glycan oligomannose core. Because of its novelty and quality, I support the publication of this manuscript in Nature Chemical Biology.

Here a small suggestion for improvement:

1. I suggest incorporating a structure-based sequence alignment of ALG3, ALG9, ALG12, with corresponding homologues/CAZY family members, highlighting the structural elements and residues involved in (i) donor, and (ii) acceptor binding sites, (iii) catalytic mechanism and (iv) those associated to CDGs.

Congratulations to the authors for this very nice work.

Referee #2

(Remarks to the Author)
This is an excellent study in comparative structural biology of membrane glycosyltransferases relevant to the eukaryotic N-glycosylation pathway. The authors report high-resolution cryo-EM structures that enable a detailed elucidation of enzyme mechanisms, particularly the recognition of lipid-linked oligosaccharides. These structures are complemented by MD simulations and mutation analyses, which together provide robust, multi-angle validation of the proposed mechanisms. The selections of donor-substrate variants for MD and the locations of engineered mutations are appropriate and well justified. Key findings include (i) both shared and distinct structural features across the three enzymes examined, (ii) an inverting catalytic mechanism, and (iii) visualization of LLO dynamics via MD initiated from experimentally determined structures. Although the manuscript does not fully detail the coupled enzymatic reaction using TbSTT3, the sample preparation, measurements, and analytical procedures appear technically sound.
Overall, the paper is well written and provides new molecular-level insights into the mechanisms of transmembrane glycosyltransferases that underpin precise eukaryotic N-glycosylation. It also represents a significant practical advance for glycoprotein engineering and is likely to open innovative avenues.

Main points
1. Resolution of glycopeptide lengths (Ext. Fig. 9; alg9 W103F, E104A, E104Q assays)

In the Tricine SDS–PAGE, differences in migration between glycopeptides bearing distinct glycan lengths are difficult to discern by eye. Please provide a gel image with improved resolution or, preferably, an orthogonal readout (e.g., MALDI- or ESI-MS) that clearly distinguishes the product mass distribution.
2. Interpretation of donor mannose density (p. 8)
The manuscript notes that "cryo-EM density for the donor mannose was not observed, possibly due to high mobility (see MD simulations below)." An additional possibility is hydrolysis of Dol-P-Man under the EM/assay conditions, which would reduce donor occupancy.

Minor points
1. linkage annotations (Fig. 1a and p. 4)
Glycosidic linkages are introduced early in the text, but explicit labeling appears only in Fig. 4, which is less accessible on a first read. Please consider adding linkage labels directly to the oligosaccharide schematic in Fig. 1a.
2. Coupled assay with TbSTT3 (p. 5)
If applicable, please clarify that TbSTT3 can transfer LLOs of different glycan lengths to the peptide acceptor in the coupled assay, and point to supporting data.
3. Comparable structural analysis (p. 6)
In the "Comparable structural analysis" section, a brief phylogenetic overview would strengthen the narrative. For example, a tree comparing ALG3 with ALG9/ALG12 across higher vs lower eukaryotes, using ALG6 as an outgroup if appropriate. A relevant reference is: Oriol et al., Mol. Biol. Evol. 19 (2002): 1451–1463 ("Common origin and evolution of glycosyltransferases using Dol-P-monosaccharides as donor substrates").
4. CH–π interactions (pp. 8–9)
The text refers to CH–π interactions (Tyr437 with the c mannose on p. 8; Trp256 with the c mannose on p. 9). I may have missed them, but I could not locate the corresponding figure panels.
5. Linkage symbols (Fig. 4d,e)
In the glycan cartoons above the lanes, please double-check the linkage notations for accuracy and consistency with the main text.

Referee #3

(Remarks to the Author)
Alexander et al provide a comprehensive structural and functional dissection of the ALG3, ALG9 and ALG12 mannosyltransferases that are involved in LLO biosynthesis after flipping of the glycolipid to the ER luminal face. The authors expressed and purified active ALG3, ALG9 and ALG12 from a variety of eukaryotic homologs, and determined specificities and activities using a previously described acceptor peptide assay. High-resolution cryoEM was carried out with the aid of newly developed Fabs, as well as a Fab binding nanobody. Structures of donor+acceptor ligand complexes of ALG3, ALG9 and ALG12 reveal the molecular basis for acceptor substrate selectivity and the overall 'logic' of LLO biosynthesis, with MD simulations also revealing basis of Dol-P-Man donor substrate selectivity. Finally, several clinical mutations are mapped onto the solved structures, and possible mechanistic implications discussed.

Overall, this is a robust and insightful piece of work that increases our understanding of N-glycan LLO biosynthesis. The study is presented clearly, and in line with the quality that I would expect from this team.

I have a few technical queries, otherwise would be happy to recommend publication.

1- The authors state that the Man sugar in the Dol-P-Man donor substrate of the ALG9 and ALG12 complexes is likely not observable due to disorder. I am not convinced, as I would still expect to see diffuse density around the area of the sugar, as the overall bound glycolipid is fixed. Can it be confirmed that the Dol-P-Man is not hydrolysed upon incubation with the (mutant) enzymes. If the enzymes mediate non-productive Dol-P-Man hydrolysis, that seems to be an important mechanistic aspect worth mentioning, as it could have implications for the biosynthesis pathway.

2- The Fabs do not appear affect ALG3/9/12 enzyme activity when assayed at endpoint. But do they affect kinetics at all? Can the authors run a timecourse to confirm there is no slowdown of activity compared to unbound ALG enzymes. I appreciate the typical assay was run in 30-60 s, but lower amounts of enzyme could be used to get slower kinetics.

3- Some of the activity assay gels are very hard to make out e.g. Ext Data 1e is very unclear, as there also seems to be a negative sloping trend in the bands across the gel. Ext data Fig 9 is also not clear, which is important as it pertains to mutant activities. Can these be redone and run out for longer?

Minor points
- At least one of the references seems incorrect? P15 ref 69 appears twice. In its second occurrence (in the conclusions) 69 refers to bump hole engineering in the text, but the bibliography refers to "molecular clinical description of first US CDGs?". This is the only one I have spotted but may be others.

- As I understand it, ALG3 and ALG12 were solved in detergent micelles, and ALG9 after nanodisc reconstitution. It is unclear to me how substrate complexes were generated for ALG9 – whether they were made with ALG9 in detergent prior to nanodisc and then reconstituted together, or added after to fully reconstituted nanodiscs? From the methods "50 µM Dol25-PP-GlcNAc2Man6 or Dol25-PP-GlcNAc2Man8 acceptor substrate and 100 µM Dol25-P-Man donor substrate were

included”, my read is probably the latter, but it would be good to be explicit.

Version 1:

Reviewer comments:

Referee #1

(Remarks to the Author)
The authors have thoroughly addressed all my comments. Congratulations once again on this excellent contribution.

Referee #2

(Remarks to the Author)
Thank you for your careful and sincere responses to my previous review. The authors have satisfactorily addressed all concerns, and the revisions have improved the clarity and robustness of the work. I have no further comments. The manuscript is now ready for publication.

Referee #3

(Remarks to the Author)
The authors have addressed my comments. Congratulations on a nice piece of work.

Open Access This Peer Review File is licensed under a Creative Commons Attribution 4.0 International License, which permits use, sharing, adaptation, distribution and reproduction in any medium or format, as long as you give appropriate credit to the original author(s) and the source, provide a link to the Creative Commons license, and indicate if changes were made.
In cases where reviewers are anonymous, credit should be given to 'Anonymous Referee' and the source.
The images or other third party material in this Peer Review File are included in the article’s Creative Commons license, unless indicated otherwise in a credit line to the material. If material is not included in the article’s Creative Commons license and your intended use is not permitted by statutory regulation or exceeds the permitted use, you will need to obtain permission directly from the copyright holder.
To view a copy of this license, visit https://creativecommons.org/licenses/by/4.0/

We thank the reviewers for their enthusiastic support and feedback, which helped strengthen our manuscript. Below are our responses in blue italics.

Referee #1 (Remarks to the Author):

In this work, Alexander and co-workers report structures of ALG3, ALG9 and ALG12 bound to chemo-enzymatically generated substrate analogues. Specifically, the authors present cryo-EM structures of (i) ALG3 from Saccharomyces cerevisiae (ScALG3) in complex with Dol25-PP-GlcNAc2Man5 and Dol25-P-Man at 3.3 A, (ii) ALG9-D82A from Homo sapiens (HsALG9) in complex with Dol25-PP-GlcNAc2Man6 and Dol25-PP-GlcNAc2Man8, both in complex Dol25-P-Man at 3.0 A and 3.1 A, respectively, and ALG12-E35Q from Gallus gallus (GgALG12) in complex with Dol25-PP-GlcNAc2Man7 and Dol25-P-Man at 2.6 A. In combination with thorough mutagenesis studies and molecular dynamics simulations, these structures reveal the mechanism of GlcNAc2Man5 to GlcNAc2Man9 biosynthesis in four sequential steps. I really enjoyed reading this manuscript. It is clearly and well written and the interpretation of the experimental data support the proposed model for assembly of the N-glycan oligomannose core. Because of its novelty and quality, I support the publication of this manuscript in Nature Chemical Biology.

Here a small suggestion for improvement:

1. I suggest incorporating a structure-based sequence alignment of ALG3, ALG9, ALG12, with corresponding homologues/CAZY family members, highlighting the structural elements and residues involved in (i) donor, and (ii) acceptor binding sites, (iii) catalytic mechanism and (iv) those associated to CDGs.

Congratulations to the authors for this very nice work.
We thank the referee for their comments and enthusiastic review of our paper. As suggested, we have now included annotated amino acid sequence alignments for homologues of ALG3, ALG9 and ALG12 in a new figure (Extended Data Fig. 3).

Referee #2 (Remarks to the Author):

This is an excellent study in comparative structural biology of membrane glycosyltransferases relevant to the eukaryotic N-glycosylation pathway. The authors report high-resolution cryo-EM structures that enable a detailed elucidation of enzyme mechanisms, particularly the recognition of lipid-linked oligosaccharides. These structures are complemented by MD simulations and mutation analyses, which together provide robust, multi-angle validation of the proposed mechanisms. The selections of donor-substrate variants for MD and the locations of engineered mutations are appropriate and well justified.
Key findings include (i) both shared and distinct structural features across the three enzymes examined, (ii) an inverting catalytic mechanism, and (iii) visualization of LLO dynamics via MD initiated from experimentally determined structures. Although the manuscript does not fully detail the coupled enzymatic reaction using TbSTT3, the sample preparation, measurements, and analytical procedures appear technically sound.
Overall, the paper is well written and provides new molecular-level insights into the mechanisms of transmembrane glycosyltransferases that underpin precise eukaryotic N-glycosylation. It also represents a significant practical advance for glycoprotein engineering and is likely to open innovative avenues.
We thank the referee for their support of our paper and suggestions for improvement. We have clarified the activity assays in the text on p. 5.

Main points
1. Resolution of glycopeptide lengths (Ext. Fig. 9; alg9 W103F, E104A, E104Q assays)
In the Tricine SDS–PAGE, differences in migration between glycopeptides bearing distinct glycan lengths are difficult to discern by eye. Please provide a gel image with improved resolution or, preferably, an orthogonal readout (e.g., MALDI- or ESI-MS) that clearly distinguishes the product mass distribution.
We agree the shifts are small and have included a new gel with better separation in Extended Data Fig. 12 (new numbering) for ALG9 W103F, E104A, E104Q mutants. Unfortunately, completion of the

B or C branches (the two reactions catalysed by ALG9) results in especially small shifts even with optimised conditions. Despite the small shifts the tricine gels provide reproducible data when appropriate controls are used. As suggested, we then performed ESI-MS on a subset of samples assayed in Extended Data Fig. 12 (new numbering) to verify our interpretation. These new data are now included in Fig. 6e and Extended Data Fig. 12b-o.

2. Interpretation of donor mannose density (p. 8)
The manuscript notes that "cryo-EM density for the donor mannose was not observed, possibly due to high mobility (see MD simulations below)." An additional possibility is hydrolysis of Dol-P-Man under the EM/assay conditions, which would reduce donor occupancy.
Thank you for raising this point. This result initially puzzled us as well. To better understand the missing donor mannose density in our HsALG9 and GgALG12 ternary structures, we have included additional in vitro *and* in silico *experiments in this manuscript.*

We first titrated donor substrate to find the minimum amount of donor substrate required to completely react with a given amount of acceptor substrate using WT ALG9 or ALG12. Using these substrate concentrations, we then added either a 2-fold molar excess of ALG9-D82A, ALG12-E35Q over the acceptor substrate or an equal volume of buffer. By incubating with an excess of enzyme, we reasoned our system could be used to detect donor hydrolysis even in the absence of substrate turnover. The samples were incubated for 1 hour on ice, to simulate conditions directly prior to cryo-EM grid vitrification. Following the incubation on ice, each sample was split into three tubes and treated as following:

1. *Nothing added (negative control)*
2. *Added WT ALG9 or ALG12 enzyme*
3. *Added WT ALG9 or ALG12 enzyme with additional donor (positive control)*

The LLOs generated in each tube were then transferred to a fluorescent peptide and run on a gel. We expected the following results for each sample:

1. *Glycopeptide with the original acceptor substrate (due to the catalytic mutant being used in the first step)*
2. *If donor hydrolysis occurred during the incubation on ice, then we expected a glycopeptide with partial or no extension of the acceptor substate. Conversely, if no donor hydrolysis occurred then we expect a glycopeptide with a fully extended acceptor substrate.*
3. *As extra donor was added we expect full extension of the acceptor substrate.*

For sample 2 with ALG9-D82A, ALG12-E35Q or buffer we observed full extension of the acceptor substrate, demonstrating that donor hydrolysis is not occurring in our system (New data: Extended Data fig. 7).

To further investigate whether the catalytic mutant that we used could be causing increased movement of the donor mannose, we also performed additional molecular dynamics simulations (800 ns with 5 replicates) with Dol25-P-Man and the catalytic mutant of ALG9 used for the cryo-EM studies (D82A). These new data, shown in Fig. 6d and Extended Data Fig. 10h, suggest the donor sugar to be highly mobile with the D82A mutant, possibly due to the increased room and lack of charge due in the active site.

We believe these experimental results, in conjunction with the MD simulations, point to the lack of donor density likely being due to increased mobility of the donor mannose.

Minor points
1. linkage annotations (Fig. 1a and p. 4)
Glycosidic linkages are introduced early in the text, but explicit labeling appears only in Fig. 4, which is less accessible on a first read. Please consider adding linkage labels directly to the oligosaccharide schematic in Fig. 1a.
We agree and have updated Fig. 1a to include the linkage labels as suggested.

2. Coupled assay with TbSTT3 (p. 5)

If applicable, please clarify that TbSTT3 can transfer LLOs of different glycan lengths to the peptide acceptor in the coupled assay, and point to supporting data.
We think the referee may have missed Fig. 1b where we demonstrate the transfer of $GlcNAc_2Man_5$, $GlcNAc_2Man_6$, $GlcNAc_2Man_7$, $GlcNAc_2Man_8$, $GlcNAc_2Man_9$, glycans to the fluorescently labelled TAMRA-YANATS-NH_2 peptide using TbSTT3B. *Additionally, previous studies have documented the LLOs TbSTT3A and TbSTT3B can transfer:*

1. Poljak, K., Breitling, J., Gauss, R., Rugarabamu, G., Pellanda, M., & Aebi, M. (2017). Analysis of substrate specificity of Trypanosoma brucei oligosaccharyltransferases (OSTs) by functional expression of domain-swapped chimeras in yeast. *Journal of Biological Chemistry*, *292*(49), 20342-20352.
2. Izquierdo, L., Mehlert, A., & Ferguson, M. A. (2012). The lipid-linked oligosaccharide donor specificities of Trypanosoma brucei oligosaccharyltransferases. *Glycobiology*, *22*(5), 696-703.

3. Comparable structural analysis (p. 6)
In the "Comparable structural analysis" section, a brief phylogenetic overview would strengthen the narrative. For example, a tree comparing ALG3 with ALG9/ALG12 across higher vs lower eukaryotes, using ALG6 as an outgroup if appropriate. A relevant reference is: Oriol et al., Mol. Biol. Evol. 19 (2002): 1451–1463 ("Common origin and evolution of glycosyltransferases using Dol-P-monosaccharides as donor substrates").
Thank you for suggesting this reference. We have included this new citation on p. 6 in the redrafted manuscript.

4. CH–π interactions (pp. 8–9)
The text refers to CH–π interactions (Tyr437 with the c mannose on p. 8; Trp256 with the c mannose on p. 9). I may have missed them, but I could not locate the corresponding figure panels.
Yes, these interactions were not included in figures as it becomes difficult to see the acceptor substrate, the acceptor substrate density and the interacting residues when these residues are displayed. Instead, we have shown these interactions for ALG3, ALG9 and ALG12 in a new figure without cryo-EM density (Extended Data Fig. 6a-c).

5. Linkage symbols (Fig. 4d,e)
In the glycan cartoons above the lanes, please double-check the linkage notations for accuracy and consistency with the main text.
We have clarified in the figure legend that lanes with the glycopeptide cartoons above them serve as glycopeptide standards that allow comparison with the experimental samples loaded on the gel. We intended these samples to serve as guides for the comparing the retention of the glycopeptides, like a protein ladder is used for the comparison of proteins. They do not necessarily represent the glycan products of the reaction. We have indicated the acceptor substrate used in the reactions above the gel and have edited Fig. 4g to more clearly show which reactions are being attempted in Fig. 4d,e.

Referee #3 (Remarks to the Author):

Alexander et al provide a comprehensive structural and functional dissection of the ALG3, ALG9 and ALG12 mannosyltransferases that are involved in LLO biosynthesis after flipping of the glycolipid to the ER luminal face. The authors expressed and purified active ALG3, ALG9 and ALG12 from a variety of eukaryotic homologs, and determined specificities and activities using a previously described acceptor peptide assay. High-resolution cryoEM was carried out with the aid of newly developed Fabs, as well as a Fab binding nanobody. Structures of donor+acceptor ligand complexes of ALG3, ALG9 and ALG12 reveal the molecular basis for acceptor substrate selectivity and the overall 'logic' of LLO biosynthesis, with MD simulations also revealing basis of Dol-P-Man donor substrate selectivity. Finally, several clinical mutations are mapped onto the solved structures, and possible mechanistic implications discussed.

Overall, this is a robust and insightful piece of work that increases our understanding of N-glycan LLO biosynthesis. The study is presented clearly, and in line with the quality that I would expect from this team.

I have a few technical queries, otherwise would be happy to recommend publication.

The authors thank the reviewer for their appreciation of our work and thoughtful suggestions.

1- The authors state that the Man sugar in the Dol-P-Man donor substrate of the ALG9 and ALG12 complexes is likely not observable due to disorder. I am not convinced, as I would still expect to see diffuse density around the area of the sugar, as the overall bound glycolipid is fixed. Can it be confirmed that the Dol-P-Man is not hydrolysed upon incubation with the (mutant) enzymes. If the enzymes mediate non-productive Dol-P-Man hydrolysis, that seems to be an important mechanistic aspect worth mentioning, as it could have implications for the biosynthesis pathway.
Thank you for raising this point. This result initially puzzled us as well. To better understand the missing donor mannose density in our HsALG9 and GgALG12 ternary structures, we have included additional in vitro *and* in silico *experiments in this manuscript.*

We first titrated donor substrate to find the minimum amount of donor substrate required to completely react with a given amount of acceptor substrate using WT ALG9 or ALG12. Using these substrate concentrations, we then added either a 2-fold molar excess of ALG9-D82A, ALG12-E35Q over the acceptor substrate or an equal volume of buffer. By incubating with an excess of enzyme, we reasoned our system could be used to detect donor hydrolysis even in the absence of substrate turnover. The samples were incubated for 1 hour on ice, to simulate conditions directly prior to cryo-EM grid vitrification. Following the incubation on ice, each sample was split into three tubes and treated as following:

4. *Nothing added (negative control)*
5. *Added WT ALG9 or ALG12 enzyme*
6. *Added WT ALG9 or ALG12 enzyme with additional donor (positive control)*

The LLOs generated in each tube were then transferred to a fluorescent peptide and run on a gel. We expected the following results for each sample:

4. *Glycopeptide with the original acceptor substrate (due to the catalytic mutant being used in the first step)*
5. *If donor hydrolysis occurred during the incubation on ice, then we expected a glycopeptide with partial or no extension of the acceptor substate. Conversely, if no donor hydrolysis occurred then we expect a glycopeptide with a fully extended acceptor substrate.*
6. *As extra donor was added we expect full extension of the acceptor substrate.*

For sample 2 with ALG9-D82A, ALG12-E35Q or buffer we observed full extension of the acceptor substrate, demonstrating that donor hydrolysis is not occurring in our system (New data: Extended Data fig. 7).

To further investigate whether the catalytic mutant that we used could be causing increased movement of the donor mannose, we also performed additional molecular dynamics simulations (800 ns with 5 replicates) with Dol25-P-Man and the catalytic mutant of ALG9 used for the cryo-EM studies (D82A). These new data, shown in Fig. 6d and Extended Data Fig. 10h, suggest the donor sugar to be highly mobile with the D82A mutant, possibly due to the increased room and lack of charge due in the active site.

We believe these experimental results, in conjunction with the MD simulations, point to the lack of donor density likely being due to increased mobility of the donor mannose.

2- The Fabs do not appear affect ALG3/9/12 enzyme activity when assayed at endpoint. But do they affect kinetics at all? Can the authors run a timecourse to confirm there is no slowdown of activity compared to unbound ALG enzymes. I appreciate the typical assay was run in 30-60 s, but lower amounts of enzyme could be used to get slower kinetics.

Given the relatively poor separation of the different glycans on tricine gel obtaining quantitative kinetic data using these techniques is quite challenging. However, we have performed time course assays as suggested with a 10-fold molar excess of Fab over ALG enzyme using reduced amount of enzyme (Supporting data fig. 1, shown below). Under these conditions complete transfer of donor Man to the acceptor substrate takes 5-10 minutes. For each enzyme our assay fails to find a difference in activity between samples with or without Fab. In the context of structural studies, we believe these results

further demonstrate that the Fabs used in this study have minimal, if any, effect on ALG enzyme activity.

Redacted

Supporting data figure 1: Activity assays of ALG enzymes with and without synthetic Fab. *Time course assay for ScALG3, Dol25-PP-GlcNAc$_2$Man$_5$ and Dol25-P-Man* ***a,*** *without and* ***b,*** *with Fab Sc3-16. Time course assay for HsALG9, Dol25-PP-GlcNAc$_2$Man$_6$ and Dol25-P-Man* ***c,*** *without and* ***d,*** *with Fab Hs9-8. Time course assay for GgALG12, Dol25-PP-GlcNAc$_2$Man$_7$ and Dol25-P-Man* ***e,*** *without and* ***f,*** *with Fab Gg12-11. The incubation time for each sample are indicated above the lanes. Two glycopeptide standards are included on either end of each gel. A dashed blue line is overlayed the gels; reactant glycans migrate below the line while product glycans are observed above the line. Reactions were performed with 10 nM ScALG3 or HsALG9 while 25 nM was used for GgALG12. Activity assays were conducted with 10 µM acceptor and 20 µM donor substrates at room temperature. The reaction products were transferred to a fluorescent peptide (TAMRA-YA**N**ATS-NH$_2$) using TbSTT3B and separated by tricine gel electrophoresis.*

3- Some of the activity assay gels are very hard to make out e.g. Ext Data 1e is very unclear, as there also seems to be a negative sloping trend in the bands across the gel. Ext data Fig 9 is also not clear, which is important as it pertains to mutant activities. Can these be redone and run out for longer?
We agree the shifts are small and have included a new gel in Extended Data Fig. 1e. Additionally we have edited this figure to make it more clear which conditions are active, inactive or have intermediate activity.

We agree the shifts are small and have included a new gel with better separation in Extended Data Fig. 12 (new numbering) for ALG9 W103F, E104A, E104Q mutants. Unfortunately, completion of the B or C branches (the two reactions catalysed by ALG9) results in especially small shifts even with optimised conditions. Despite the small shifts the tricine gels provide reproducible data when appropriate controls are used. We then performed ESI-MS on a subset of samples assayed in Extended Data Fig. 12 (new numbering) to verify our interpretation. These new data are now included in Fig. 6e and Extended Data Fig. 12b-o.

Minor points
- At least one of the references seems incorrect? P15 ref 69 appears twice. In its second occurrence (in the conclusions) 69 refers to bump hole engineering in the text, but the bibliography refers to "molecular clinical description of first US CDGs?". This is the only one I have spotted but may be others.
Thank you for noticing this; the references have been corrected.

- As I understand it, ALG3 and ALG12 were solved in detergent micelles, and ALG9 after nanodisc reconstitution. It is unclear to me how substrate complexes were generated for ALG9 – whether they were made with ALG9 in detergent prior to nanodisc and then reconstituted together, or added after to fully reconstituted nanodiscs? From the methods "50 µM Dol25-PP-GlcNAc2Man6 or Dol25-PP-GlcNAc2Man8 acceptor substrate and 100 µM Dol25-P-Man donor substrate were included", my read is probably the latter, but it would be good to be explicit.

The text has been amended to explicitly state the substrates were added after nanodisc reconstitution. The text now reads: 'HsALG9, reconstituted in nanodiscs, was concentrated to 12 µM before the addition of 50 µM Dol25-PP-GlcNAc$_2$Man$_6$ or Dol25-PP-GlcNAc$_2$Man$_8$ acceptor substrate and 100 µM Dol25-P-Man donor substrate.'

We thank the reviewers for their support and feedback, which helped strengthen our manuscript. No further changes in response to reviewer comments were implemented.

Referee #1:
Remarks to the Author:
The authors have thoroughly addressed all my comments. Congratulations once again on this excellent contribution.

Referee #2:
Remarks to the Author:
Thank you for your careful and sincere responses to my previous review. The authors have satisfactorily addressed all concerns, and the revisions have improved the clarity and robustness of the work. I have no further comments. The manuscript is now ready for publication.

Referee #3:
Remarks to the Author:
The authors have addressed my comments. Congratulations on a nice piece of work.
