# Supplementary material for: Structures of ALG3/9/12 reveal the assembly logic of the N-glycan oligomannose core
Source: Nat Chem Biol. 2026 Mar 10;22(9):1501–13. doi: 10.1038/s41589-026-02164-7 (PMC13503287; doi:10.1038/s41589-026-02164-7)

Extended Data Fig. 1b

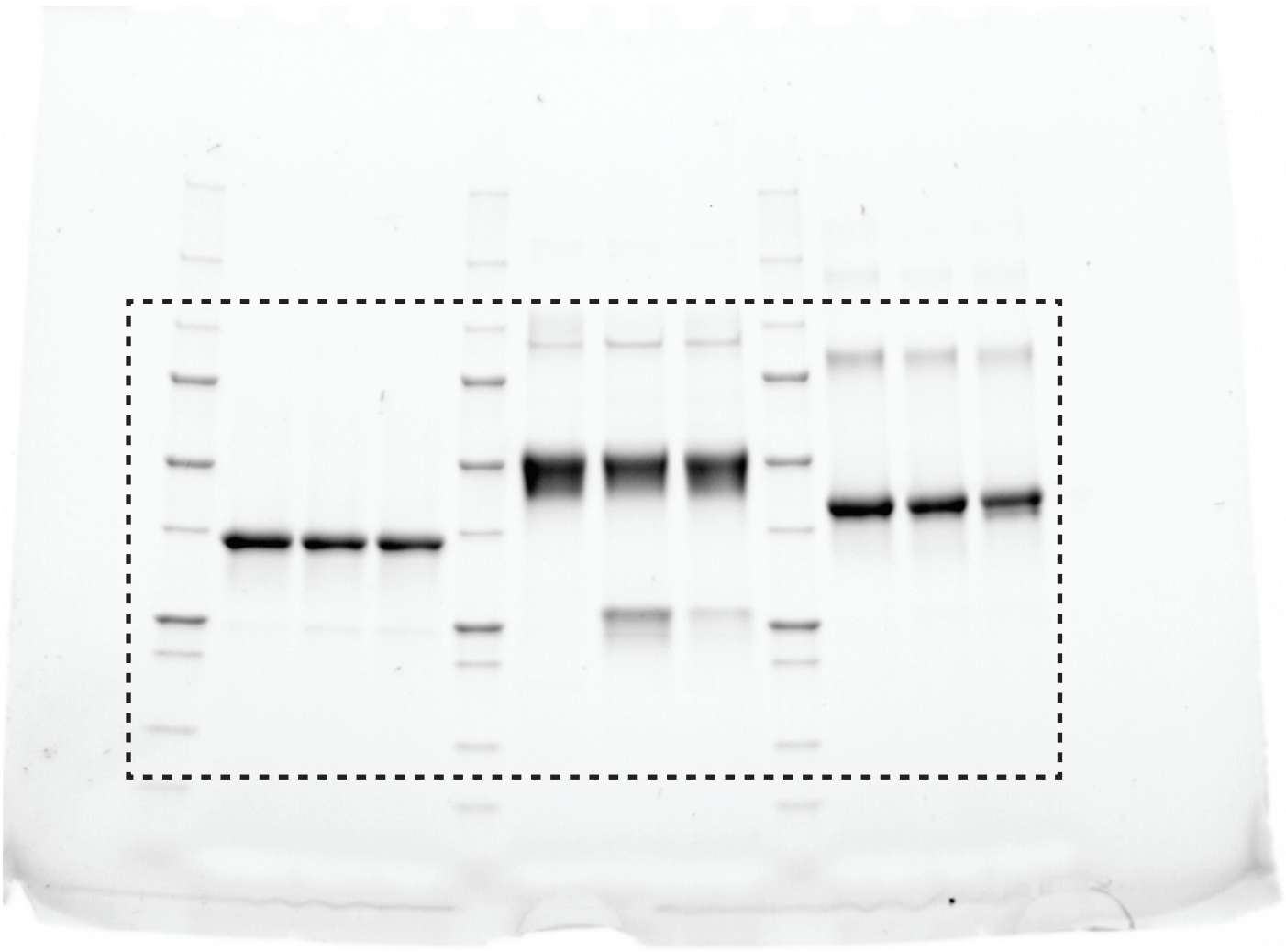

Extended Data Fig. 1c

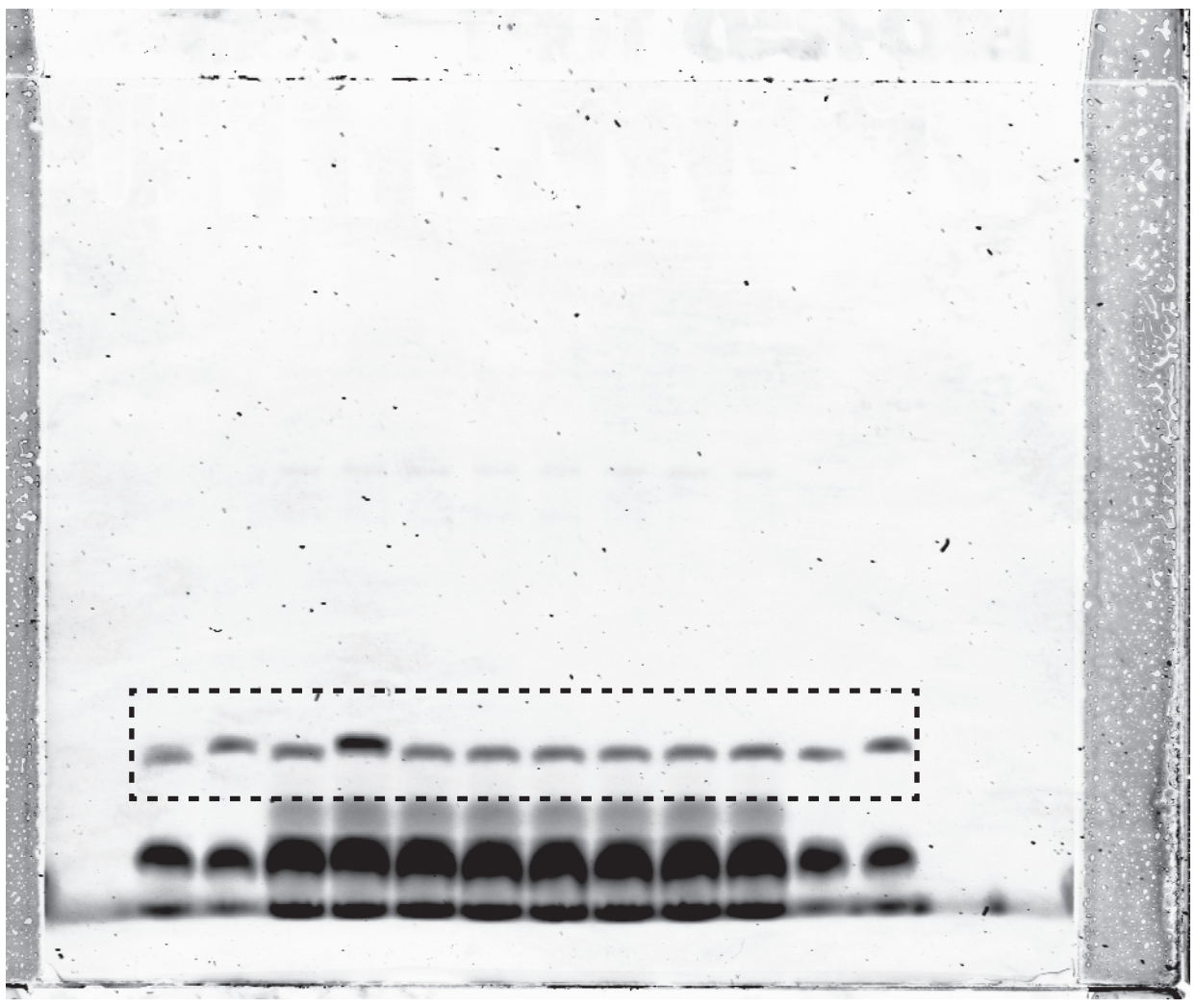

Extended Data Fig. 1d

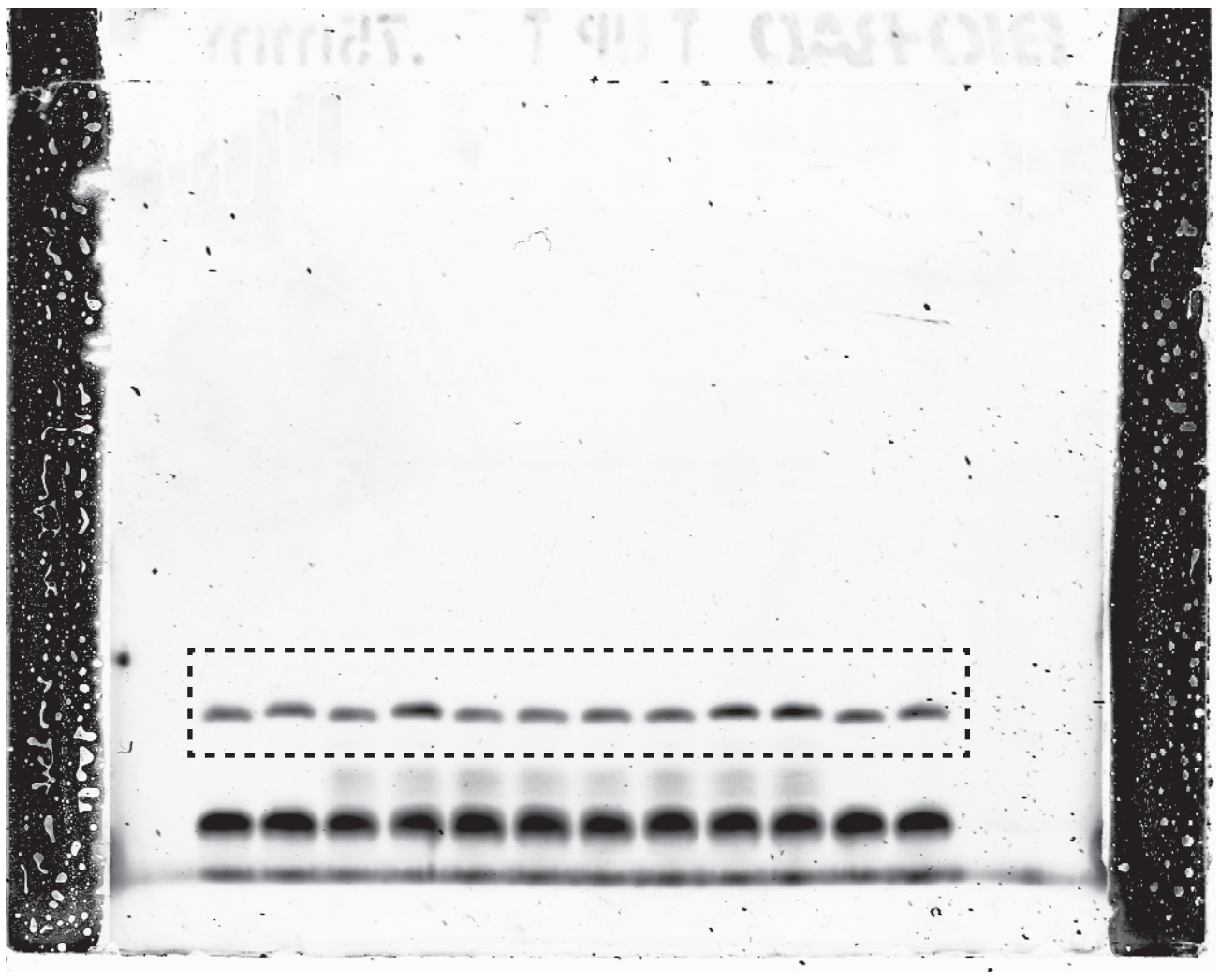

Extended Data Fig. 1e

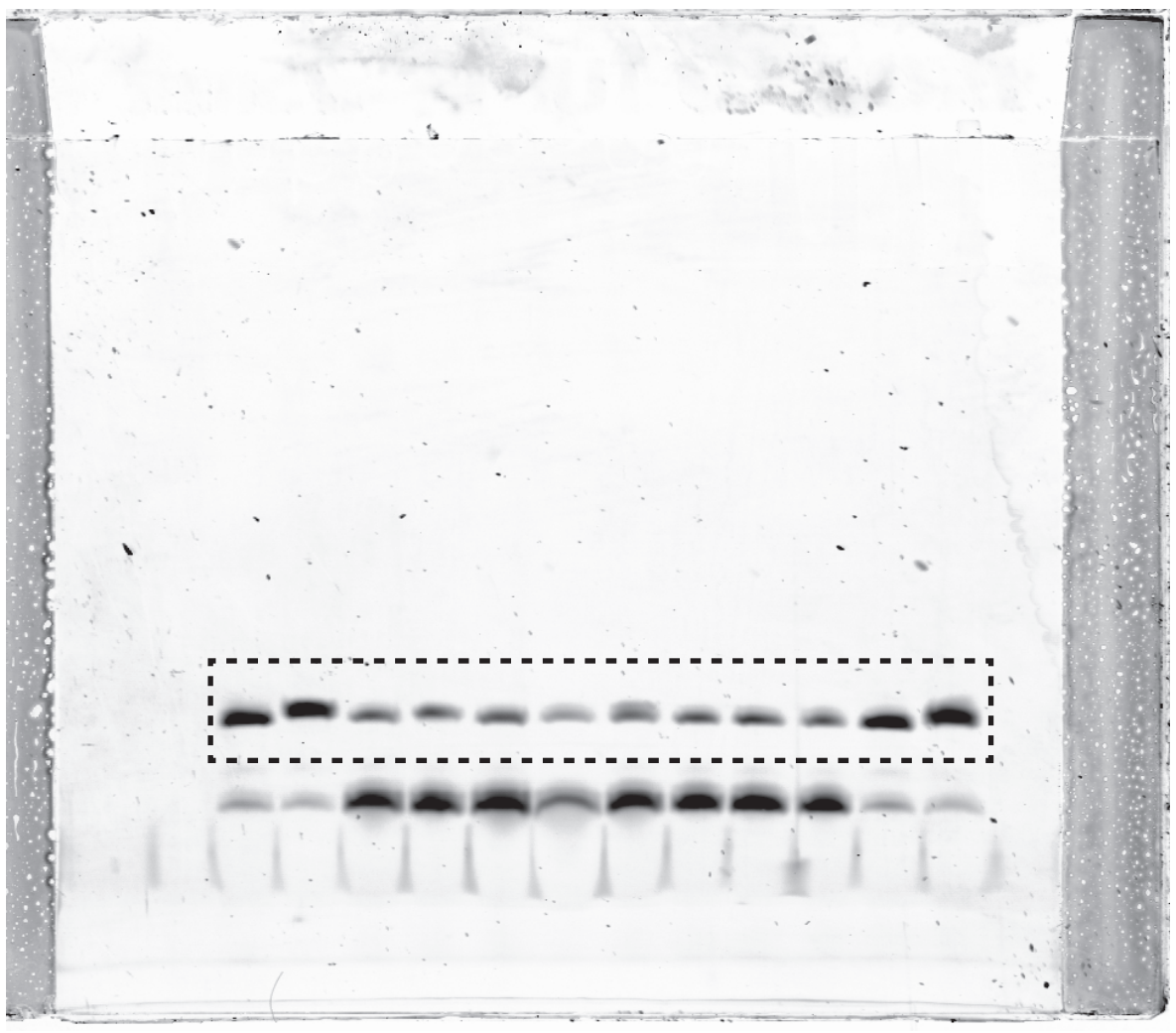

Supplement: Supplementary file 7 [file 41589_2026_2164_MOESM7_ESM.pdf]
